# The antimicrobial peptide EM86 loaded to gamma-irradiated sodium alginate/polyvinyl alcohol electrospun nanofibrous dressing treated multidrug-resistant *Pseudomonas aeruginosa* wound infections in BALB/c mice

**DOI:** 10.3389/fbioe.2026.1776154

**Published:** 2026-04-07

**Authors:** Samar Essam Metwally, Hala Abd Allah Farrag, Hassan Ahmed Abd El-Rehim, Rehab Nabil Shamma, Amal Emad Ali, Omneya Mohamed Helmy

**Affiliations:** 1 Post Graduate Program, Faculty of Pharmacy, Cairo University, Cairo, Egypt; 2 Department of Drug Radiation Research, National Center for Radiation Research and Technology (NCRRT), Egyptian Atomic Energy Authority (EAEA), Cairo, Egypt; 3 Polymer Chemistry Department, National Center for Radiation Research and Technology (NCRRT), Egyptian Atomic Energy Authority (EAEA), Cairo, Egypt; 4 Department of Pharmaceutics and Industrial Pharmacy, Faculty of Pharmacy, Cairo University, Cairo, Egypt; 5 Department of Microbiology and Immunology, Faculty of Pharmacy Cairo University, Cairo, Egypt

**Keywords:** anti-Gram-negative, antimicrobial peptides, computer-aided design, electrospun nanofibers, gamma-irradiation, *Pseudomonas aeruginosa*, wound dressings

## Abstract

**Background:**

Treating infected wounds is challenging, and conventional dressings delay healing. Resistance to the last-resort antibiotics is on the rise. Antimicrobial peptides (AMPs) offer advantages over traditional antibiotics against multidrug-resistant (MDR) bacterial strains. We aimed to use a computer-aided approach to design an anti-Gram-negative AMP with reduced mammalian cell toxicity and to functionalize an electrospun nanofibrous dressing with the AMP for *in vitro* and *vivo* evaluation.

**Methods:**

Colistin and polymyxin sequences were modified to generate analogs using a template-based approach. The membrane activity was predicted using the collection of antimicrobial peptide database. Three shortlisted sequences were synthesized, and their minimum inhibitory concentrations (MICs) and minimum bactericidal concentrations (MBCs) against MDR Gram-negative bacterial isolates were determined. The time-kill kinetics of the potent candidate against *Pseudomonas aeruginosa* SM016 were analyzed, and toxicity to human skin fibroblasts (HSF) was determined by the MTT assay. A sodium alginate (SA)/polyvinyl alcohol (PVA) nanofibrous wound dressing was synthesized by electrospinning. The produced scaffolds were gamma-irradiated and examined using scanning electron microscopy (SEM). The AMP was loaded onto the scaffold by physical adsorption, followed by *in vitro* and *in vivo* testing against *Pseudomonas aeruginosa* SM016 in open infected wounds in BALB/c mice.

**Results:**

EM86 exhibited bactericidal activity against all tested isolates. EM86 killed *Pseudomonas aeruginosa* SM016 within 60 min, with morphological changes indicative of cell death confirmed by SEM after 30 min of treatment. EM86 was safe to HSF(IC50 > 300 μg/mL). The polymeric mixture of 1:12.3 SA/PVA, crosslinked with 1.25% glutaraldehyde, electrospun at 30 kV, 0.045 mm/min, and 12.5 cm, produced nanofibrous dressings with a mean diameter of 238 ± 78 nm and a swelling capacity of 510%. EM86-loading was confirmed by Fourier-transform infrared spectroscopy. Fibers loaded with 35 ± 18 μg EM86 significantly reduced *P. aeruginosa* SM016 counts (P < 0.001) in open-wound infected BALB/c mice, compared to the untreated mice, after 4 days of a once-daily treatment.

**Conclusion:**

Using computer-aided approaches, we designed EM86 with a bactericidal activity against MDR Gram-negative microorganisms and reduced toxicity to HSF compared to colistin. EM86-functionalized SA/PVA nanofibrous dressings offer a better treatment alternative for colistin-resistant infections in open wounds.

## 1 Introduction

Skin and soft tissue infections (SSTIs) range from superficial, uncomplicated infections to complicated infections that deeply extend into subcutaneous tissues. Most complicated SSTIs are encountered in patients with comorbidities, such as diabetes mellitus, heart failure, chronic kidney disease, neutropenia, and cancer. SSTIs rank as the fourth-highest contributor to global disability (Karimkhani et al., 2017). Patient visits for SSTIs in the US are on the rise, with an average of 48 cases per 1,000 person-years of observation (PYO) during 2005–2009, rising to 77.5 per 1,000 PYO during 2010–2020 (Miller et al., 2015; Vella et al., 2024). Most isolated pathogens in SSTIs are *Enterococci*, *Escherichia coli*, and *Pseudomonas aeruginosa* (Kofteridis et al., 2012; Puca et al., 2021). *Pseudomonas aeruginosa*, an opportunistic pathogen, poses a significant threat to individuals with compromised immune systems, leading to substantial morbidity and mortality rates of 32% (Reynolds and Kollef, 2021). Conventional treatments for SSTIs involve managing underlying infection with antibiotics and protecting the wound area from environmental pollutants to promote healing (Gizaw et al., 2018).

Antimicrobial resistant (AMR) infections are a global healthcare concern threatening humanity (Barnes et al., 2017), with an expected annual death of two million people attributed to AMR, in 2050 (Naghavi et al., 2024). *Pseudomonas aeruginosa* is included in the 2024 World Health Organization updated high-priority list of multidrug-resistant (MDR) bacterial strains (Jesudason, 2024). The world is approaching a post-antibiotic era, and even well-known last-resort antibiotics may fail against bacterial infections. With few antibiotics in the pipeline, new antimicrobial agents are needed (AL Tall et al., 2019).

Antimicrobial peptides (AMPs) are often small proteins consisting of fewer than 100 amino acids, encoded in the genome and produced by all forms of life, from prokaryotes to humans (Fjell et al., 2021; Hancock and Diamond, 2000). AMPs are produced either by ribosomal translation of mRNA or by non-ribosomal peptide synthesis, exhibiting structural and functional diversity that makes them difficult to classify, except for a broad classification based on their secondary structure (Wang, 2019). Altering even a single residue in the AMP can affect its overall net charge, hydrophobicity, molecular weight, and other criteria, leading to drastic changes in activity (Mishra et al., 2018). Most of the AMPs reported so far are classified into four groups according to their secondary structure: α-helical, β-sheets, extended, and cyclic (Nguyen et al., 2011). The Food and Drug Administration has approved several cyclic peptides over the past few years, including polymyxin B, dalbavancin, and oritavancin (Rabanal and Cajal, 2017). AMPs that exhibit α-helical and β-sheet secondary structures exert their antimicrobial activity by disrupting membrane integrity. Extended peptides are characterized by the abundance of specific amino acids and the lack of secondary structure. They exert their activity by penetrating membranes and interacting with intracellular targets, hindering the synthesis of macromolecules (proteins, DNA, and RNA), or targeting metabolic enzymes (Mishra et al., 2018). In polymyxins, the positively charged 2, 4-diaminobutyric acid residues (Dab) are electrostatically attracted to the negatively charged phosphate group of lipid A of the outer membrane of Gram-negative bacteria, followed by the displacement of Ca^2+^ and Mg^2+^ from the phosphate groups, resulting in an increased membrane permeability and cell lysis. Furthermore, the insertion of the hydrophobic fatty acyl side chain of polymyxins into the membranes facilitates disruption and induces toxicity to mammalian cells (Rhouma et al., 2016; Poirel et al., 2017). Following electrostatic interactions, monomeric, random-coiled cationic peptides in aqueous solution cross the outer bacterial membrane of Gram-negative bacteria, reaching the inner membrane, whose hydrophobicity induces the peptide’s secondary structure. This permits a deeper membrane insertion (Zelezetsky et al., 2005; Mant et al., 2019). AMPs disrupt the cell membrane, resulting in pore formation and the efflux of essential ions and nutrients. Different models of pore formation have been proposed, including toroidal pore, barrel-stave, and carpet models (Felgueiras and Amorim, 2017).

Resistance to AMPs is unlikely due to their mode of action, which involves attacking multiple low-affinity targets instead of one high-affinity target (Mahlapuu et al., 2016). The selectivity of AMPs for bacterial over eukaryotic membranes results from differences in their membrane composition (Kumar et al., 2018). Polymyxin B is a cyclic heptapeptide with a tripeptide side chain acylated at the N-terminus with a fatty acid side chain. Polymyxin B and colistin (Polymyxin E) share the same sequence except for the amino acid residue at position 6, where the D-phenylalanine in polymyxin B is substituted by D-leucine in colistin (Rhouma et al., 2016). Polymyxin B exhibits antibacterial activity against MDR Gram-negative bacteria and is used to treat urinary tract infections and meningitis caused by *Pseudomonas aeruginosa* and *Haemophilus influenzae*, respectively (Rabanal and Cajal, 2017). Colistin is a last-line drug treatment for Gram-negative bacterial infections. Tragically, colistin-resistant isolates of *Pseudomonas aeruginosa*, *Acinetobacter baumannii*, and members of Enterobacteriaceae, such as *Escherichia coli*, *Salmonella* spp., and *Klebsiella* spp., have been reported (Aghapour et al., 2019).

Physical and chemical instability, as well as proteolytic degradation, have led to the failure of this class of antimicrobials in clinical trials at some point (Zhao et al., 2016; Pachon-Ibanez et al., 2017; Wa et al., 2018). As a result, the rational design of new AMPs with improved activity and reduced toxicity is urgently needed (Porto et al., 2017). Conventional drug design and discovery methods are cost-intensive and time-consuming (Rudrapal et al., 2020). The rapid development of computer algorithms, hardware, and software has revolutionized computer-aided drug discovery and development, drastically reducing time and costs (Selvaraj et al., 2022). AMP design can begin either *de novo* (without a seed sequence) or by modifying known peptide sequences to generate optimized analogs. The design process is guided by specific parameters based on the chosen methodology. Structure prediction of the candidate sequences is performed, followed by an in-depth functional and structural analysis (Cardoso et al., 2020). IL15.3, a modified sequence of QL17 peptide isolated from *Crocodylus siamensis* hemoglobin hydrolysate, has an improved antimicrobial activity against both Gram-negative and Gram-positive bacterial strains and a decreased toxicity to eukaryotic cells (Sosiangdi et al., 2023). SP15D, a short anti-Gram-negative AMP, was *de novo* designed by a database-specific prediction algorithm available at (https://dbaasp.org/prediction), acts similarly to a cell-penetrating peptide at high concentrations; however, at its minimum inhibitory concentration (MIC), it acts differently by penetrating the cytoplasmic membrane and acting on intracellular targets, without inducing any membrane disruptions (Vishnepolsky et al., 2019).

Traditional cotton gauze dressings are often used for wound coverage; however, they do more harm than good. They adhere to the wound bed and injure the newly formed tissues during removal. Thus hindering the healing process and prolonging the treatment period in patients with an underlying health problem (Gizaw et al., 2018). Nanofibers are commonly used in biomedicine due to their unique characteristics, including a high surface area-to-volume ratio, porosity, and customizable drug-delivery properties (Gizaw et al., 2018). Nanofibrous mats have a significant potential for use as wound dressings; they are biologically inert and do not adhere to the wound bed, allowing easy removal without damaging newly formed tissues (Gizaw et al., 2018). They are produced using various techniques, including electrospinning (top-down), self-assembly (bottom-up), phase separation, and template-based methods (Samatham and Kim, 2006).

Electrospun nanofibrous mats have recently attracted significant attention; they are produced from high-molecular-weight polymers (natural or synthetic) (Gizaw et al., 2018). The technique involves applying a high voltage between the needle tip and the collector of the electrospinning device, generating electric forces that oppose the surface tension forces of the polymer solution in the needle. When they reach a critical threshold, these repulsive forces distort the surface tension of the polymer solution at the needle tip, resulting in its ejection as a Taylor cone (Sharma et al., 2015). The selection of the polymer affects the dressing’s ability to absorb exudates. Sodium alginate (SA)-based dressings absorb more fluids and swell, maintaining a humid environment that facilitates healing and prevents wound bed dryness (Gizaw et al., 2018). Although SA has high conductivity, surface tension, and viscosity that hinder spinning (Taemeh et al., 2020), it can be used in conjunction with other synthetic polymers, such as polyvinyl alcohol (PVA), which has favorable mechanical properties and flexibility. PVA interacts with SA through hydrogen bonding, thereby decreasing its conductivity, viscosity, and surface tension while improving its electrospinning potential (Mokhena et al., 2020). Furthermore, electrospun dressings can be functionalized with active biomolecules to treat infected wounds (Gizaw et al., 2018). Different techniques are used to produce drug-loaded nanofibrous wound dressings, including blending the drug with the polymer mixture before electrospinning (Duque Sánchez et al., 2016), loading the drug by physical adsorption onto the nanofibrous scaffolds (Kim and Yoo, 2010), and immobilizing drugs on nanofibrous scaffolds via chemical bonds (Yang et al., 2015). Several studies highlighted the superiority of electropun-based wound dressings in the management of infected wounds. Chitosan/Royal jelly/PVA electrospun nanofibrous dressings significantly accelerated healing of *P. aeruginosa*-infected burn wounds, with faster wound contraction, tissue repair, re-epithelialization, and collagen synthesis (Ashrafi et al., 2014). PVA/chitosan nanofiber wound dressings with incorporated N, N, N-trimethyl chitosan capped gold-silver nanoparticles were effective in the treatment of MDR *P. aeruginosa* infected wounds in 99.34% of the treated mice, within 12 days (Malik et al., 2024). The application of PVA-based nanofibrous dressings enriched with pupae oil and Prussian blue nanoparticles resulted in enhanced healing of infected wounds (Kumar et al., 2025). The PseuPha1 and RuSa1 phages, which lysed multiple clinical MDR strains of *P. aeruginosa* and *S. aureus*, were incorporated into PVA-Eudragit by electrospinning to produce nanofibers that successfully treated MDR-infected wounds in diabetic mice without inducing toxicity to mouse fibroblast cell lines (NIH3T3) (Suchithra et al., 2025). In this study, we aimed to design anti-Gram-negative AMPs using computer-aided approaches, load the most potent candidate onto an electrospun nanofibrous SA/PVA dressing, and test the peptide-loaded dressings *in vivo* in a murine infection model.

## 2 Results

### 2.1 Designing colistin analogs

A total of 358 AMPs with anti-Gram-negative activity, 40 with anti-Gram-negative and antibiofilm activities, and 21 with wound healing activity were retrieved from the antimicrobial peptide database (APD3) during the target activity search in 2019 (Supplementary Tables S1–S3). Multiple sequence alignment of the retrieved sequences, within each subgroup, revealed no conserved amino acid residues (Supplementary Figures S1 and S2). Eighty-four possible sequence modifications of colistin and polymyxin were generated by altering the type and position of amino acids, and adding a WWW motif to their N-terminus (Supplementary Table S4). All sequences were predicted to have membrane-active antimicrobial properties, as indicated by the collection of antimicrobial peptides database (CAMPR3) (http://www.camp.bicnirrh.res.in/predict/), where each sequence had a threshold ranging from 0.5 to 1, and peptides are classified as AMPs if their threshold exceeds 0.5.

The core segment hydrophobicity (CSH) of three AMPs, EM02: WWWKAKKKAAKKG, EM60: WWWKFKKKGAKKG, and EM84: WWWKAKKKFGKKG, ranged from 0.4 to 2. A hydrophobicity scale, categorizing amino acid residues by their retention time in reverse-phase high-performance liquid chromatography (RP-HPLC), was used to calculate the peptides’ core-segment hydrophobicity by summing up the hydrophobicity values of their amino acid residues and dividing them by their number, excluding arginine (R) and lysine (K) (Liu and Deber, 1998). EM02, a colistin analog, had a positive Wimley-White whole residue hydrophobicity value, obtained from the APD3 (https://aps.unmc.edu/prediction), indicating hydrophilicity, while EM60 and EM84 were more hydrophobic (Supplementary Table S4).

**FIGURE 1:**
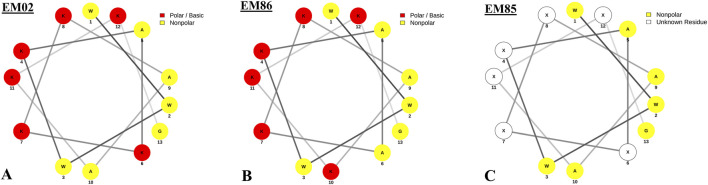
Helical wheel diagrams of EM02, EM86, and EM85: **(A)** EM02: WWWKAKKKAAKKG, with four residues on the same hydrophobic surface; **(B)** EM86: WWWKAAKKAKKKG, with an exchange of K6 and A10 positions, resulting in five residues on the same hydrophobic surface and **(C)** EM85: Replacing K in EM02 with Dab represented here as X. The red letters in the peptide sequences denote the hydrophobic residues on the same surface. The polar/basic residues are presented in red and the nonpolar residues in yellow. This was generated using the online tool (http://lbqp.unb.br/NetWheels/).

**FIGURE 2:**
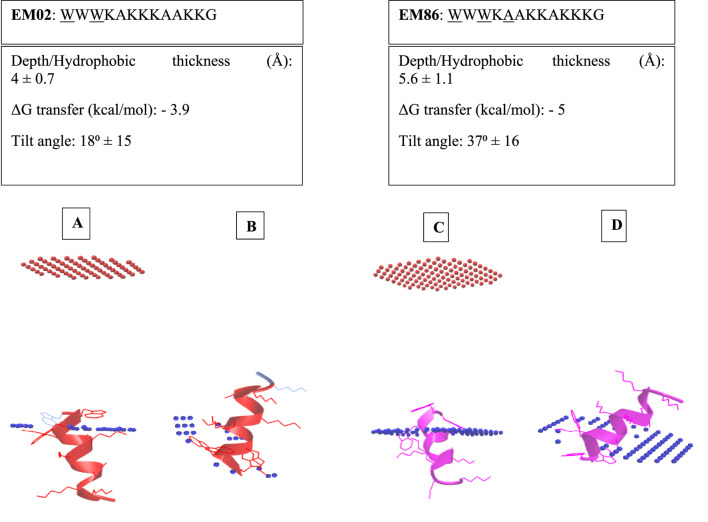
EM02 and EM86 Peptide interaction with the inner membrane of Gram-negative bacteria: **(A)** EM02 interaction with the inner membrane of Gram-negative bacteria; **(B)** EM02 interaction with the inner membrane of Gram-negative bacteria at a tilt angle 18° ± 15, with bacterial membrane damage presented as discontinued dotted blue line; **(C)** EM86 interaction with inner membrane of Gram-negative bacteria and **(D)** EM86 interaction with inner membrane of Gram-negative bacteria at a tilt angle of 37° ± 16 producing a visible damage in the inner membrane. An increase in the tilt angle from 18° ± 15 to 37° ± 16, the number of embedded residues (2–3), and the penetration depth (4 ± 0.7 to 5.6 ± 1.1 Å depth) upon altering the position of K6 and A10 of EM02 in EM86, highlights a more profound penetration of EM86 into the bacterial membrane. The inner membrane of Gram-negative bacteria is presented as a blue dotted line, while the outer membrane is presented as a red one. The embedded residues are underlined. PEP-FOLD was used to predict the 3D structure of EM02 and EM86, and the PPM online server was used to illustrate the peptide’s interaction with the inner membrane of Gram-negative bacteria.

EM02 was selected for further structural modifications; EM85: WWW(Dab)A(Dab)(Dab)(Dab)AA(Dab)(Dab)G, and EM86: WWWKAAKKAKKKG were generated; their net charge and molecular weight were obtained from the APD3 (https://aps.unmc.edu/prediction) (Table 1). The spatial arrangement of the amino acids of EM02, EM86, and EM85, generated using the online tool (http://lbqp.unb.br/NetWheels/), is shown in Figures 1A–C. EM02 had 4 residues on the same hydrophobic surface; exchanging the K6 and A10 positions yielded 5 residues on that surface in EM86. The 3D fold structures of EM02 and EM86 were predicted by PEP-FOLD 3 (https://mobyle.rpbs.univ-paris-diderot.fr/cgi-bin/portal.py#forms::PEP-FOLD3), and their predicted interactions with the Gram-negative bacterial membrane were illustrated by the PPM online server (https://opm.phar.umich.edu/ppm_server3_cgopm) (Figure 2). EM02 interacted with the inner membrane of Gram-negative bacteria at a tilt angle of 18° ± 15, increasing to 37° ± 16 with EM86. In addition, the number of embedded residues increased from 2 in EM02 to 3 in EM86, and the penetration depth increased from 4 ± 0.7 to 5.6 ± 1.1 Å. All this resulted in better visualization of EM86 penetration into the bacterial membrane (Figure 2).

**TABLE 1:** Sequence, length, net charge, calculated core-segment hydrophobicity, Wimley-White whole residue hydrophobicity, and molecular weight of the designed colistin analogs: EM02, EM85, and EM86.

Coded sequence	Sequence	Length (number of amino acids)	Net charge	Core-segment hydrophobicity	The Wimley-White whole-residue hydrophobicity of the peptide	Molecular weight
EM02	WWWKAKKKAAKKG	13	6	1.69	0.91 kcal/mol	1615.99
EM85	WWW(Dab)A(Dab) (Dab) (Dab)AA(Dab) (Dab)G	13	6	−	−	1447.67
EM86	WWWKAAKKAKKKG	13	6	1.69	0.91 kal/mol	1615.99

The red letters in the peptide sequences denote the hydrophobic residues on the same surface, Dab denotes 2,4-diaminobutyric acid. The core-segment hydrophobicity and Wimley-White whole residue hydrophobicity of EM85 were not calculated.

### 2.2 EM86 has a bactericidal activity against the tested Gram-negative bacterial isolates

The MIC and minimum bactericidal concentration (MBC) of freshly prepared solutions of EM02, EM85, EM86, and colistin were determined, using the broth microdilution method (BMD), against MDR *Pseudomonas aeruginosa* (n = 3)*, Klebsiella pneumoniae* (n = 1)*, and Acinetobacter baumannii* (n = 1) clinical isolates (Supplementary Table S5). EM86 exhibited bactericidal activity against all tested isolates, with the lowest MIC detected against the *Pseudomonas aeruginosa* SM016 wound isolate.

The MIC and MBC determinations were repeated using the original AMP solutions after storage for 2 weeks at − 20 °C against the *Pseudomonas aeruginosa* SM016 isolate (Table 2). An increase in the MIC and MBC values of EM86 was reported, and complete loss of activity of EM02 and EM85 was observed when using 2-week-old solutions stored at − 20 °C, compared to freshly prepared peptide solutions. Thus, the EM86 peptide was selected for further investigations.

**TABLE 2:** The minimum inhibitory concentration (MIC) and minimum bactericidal concentration (MBC) of EM02, EM85, EM86, and colistin against *Pseudomonas aeruginosa* SM016 clinical isolate.

Peptide solution	MIC (µg/mL)				MBC (µg/mL)			
	EM02	EM85	EM86	Colistin	EM02	EM85	EM86	Colistin
Freshly prepared	81 ± 60	84 ± 54	4 ± 2	2	97 ± 62	104 ± 48	8 ± 2	2
2-week-old solutions	>128	>128	16 ± 8	2	>128	>128	16 ± 8	2

### 2.3 EM86 displayed comparable time-killing kinetics to colistin

**FIGURE 3:**
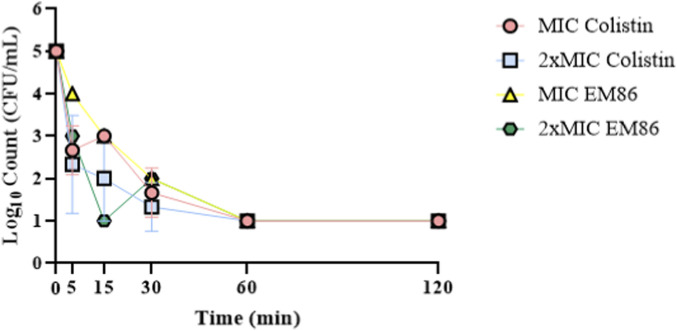
Time-killing kinetics curves of EM86 and colistin: *Pseudomonas aeruginosa* SM016 at a cell count of 5 × 10^5^ CFU/mL was treated with either 1× MIC or 2× MIC of EM86 or colistin and incubated at 37 °C. A 50 µL aliquot, taken at 5, 15, 30, 60, and 120 min time points, was subjected to viable count on Muller-Hinton agar plates. An untreated control was included. The experiment was performed in triplicate. The Log_10_ CFU/mL was plotted against time, and the limit of bacterial detection was 10 CFU/mL.

The time-kill kinetics of 1× MIC and 2× MIC of EM86 and colistin against *Pseudomonas aeruginosa* SM016 over 120 min were determined. All tested concentrations of EM86 and colistin killed *Pseudomonas aeruginosa* SM016 within 60 min (Figure 3). However, an increase in bacterial counts was recorded after 15 min and 30 min of treatment with 1× MIC colistin and 2× MIC EM86, respectively.

### 2.4 EM86-treated *Pseudomonas aeruginosa* displayed morphological changes of bacterial cell death

**FIGURE 4:**
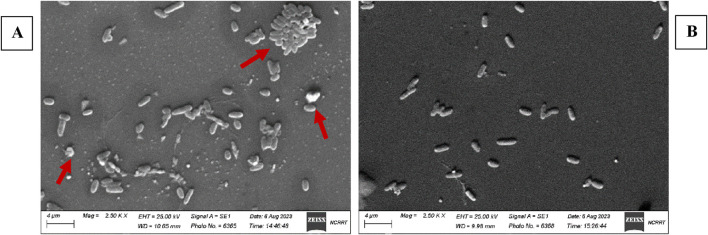
Scanning electron microscopic examination of EM86-treated *Pseudomonas aeruginosa* SM016 versus untreated bacteria: **(A)** EM86-treated *Pseudomonas aeruginosa* SM016 at a magnification power of ×25,000 showing bacterial cell clustering, distorted integrity, ballooning, and cell rupture; **(B)** untreated *Pseudomonas aeruginosa* SM016 control at a magnification power of ×25,000 showing normal rod-shaped morphology and distribution.

Scanning electron microscopic examination (SEM) of EM86-treated *Pseudomonas aeruginosa* SM016 revealed abnormal bacterial cell distribution, clustering, deformity, ballooning, and rupture with the release of cytoplasmic content (Figure 4A). However, the untreated *Pseudomonas aeruginosa* SM016 control sample showed intact bacterial cells with normal integrity and distribution (Figure 4B).

### 2.5 EM86 is safe to human skin fibroblasts

**FIGURE 5:**
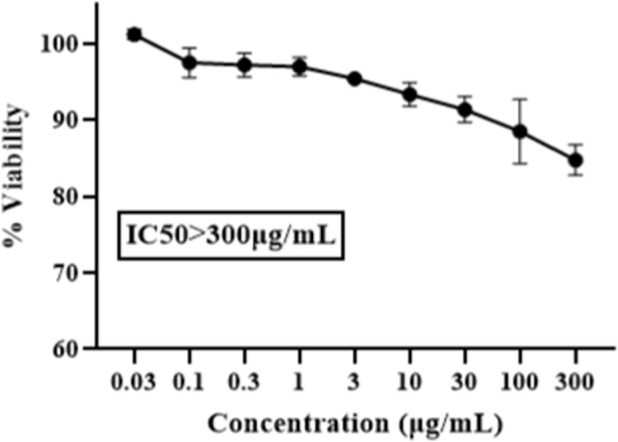
Cytotoxicity of EM86 on Human skin fibroblasts using MTT assay: the experiment was performed in triplicate, and the percentage viability was the mean of three replicates, with error bars representing the standard deviation. An IC50 > 300 μg/mL was reported.

The cytotoxicity of EM86 on human skin fibroblasts (HSF) was evaluated using the MTT assay. The tested concentrations ranged from 0.03 to 300 μg/mL, with the highest concentration showing 85% viability, indicating an IC50 greater than 300 μg/mL (Figure 5).

### 2.6 Successful production of a nanofibrous dressing by electrospinning

#### 2.6.1 The sodium alginate/polyvinyl alcohol ratio affects the produced fibers

The electrospinning process was optimized by testing one variable at a time. Polymeric SA/PVA mixtures with different SA and PVA concentrations, and different running parameters were tested. The effects of adding glutaraldehyde to the electrospinning mixture and gamma-irradiating the produced scaffolds were also tested. Preliminary studies were performed using different SA/PVA formulations, flow rate (Q), and voltage (V) (Supplementary Table S6). SA/PVA reaction mixtures of 1:6.5, 1:10, 1:12.3, 1:15.3, and 1:20 were electrospun, and the produced fibers were given scores from 0 to 3. The scoring system was as follows: electrospraying and no fiber production (score 0), low-density fibers and dried jets (score 1), moderate-density fibers (score 2), and high-density fibers (score 3). The 1:12.3 and 1:20 formulations successfully produced fibers with a score of 3 and were chosen for further experiments (Supplementary Table S6).

#### 2.6.2 Changing the running parameters affects the produced fibers

**FIGURE 6:**
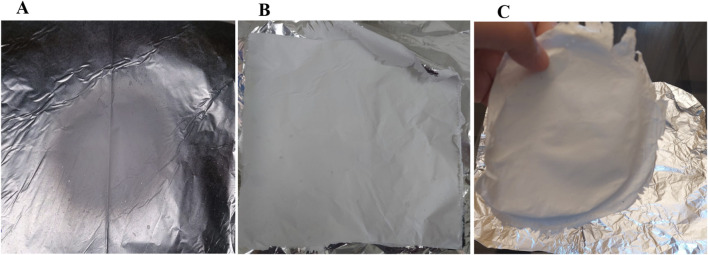
The effect of optimizing the flow rate and voltage on the density of the produced electrospun fibers: **(A)** Low-density fibers that could not be separated from the collector produced using the 1:12.3 SA/PVA ratio, a flow rate of 0.016 mm/min and voltage of 18 kV; **(B,C)** denser fibers that were easily separated from the collector produced using the 1:12.3 SA/PVA ratio, a flow rate of 0.045 mm/min and 30 kV. Both runs were operated for the same time interval.

Fibers were prepared using the selected formulations under different running parameters: Q, V, and distance to the collector (X). The mean diameter of the produced fibers was determined by SEM (Table 3). The fibers prepared using the 1:12.3 SA/PVA ratio, V of 30 kV, Q of 0.045 mm/min, and 12.5 cm from the collector had the highest fiber density, the smallest mean diameter of 238 ± 78 nm, and were easily separated from the collector. Decreasing both Q and V for the same formulation resulted in a reduced fiber density (Figure 6).

**TABLE 3:** The mean diameter of the fibers in the produced electrospun scaffolds from coded runs 1–10.

Coded Run	SA:PVA ratio	Flow rate Q (mm/min)	Voltage (kV)	Fiber mean diameter ± SD
1	1:20	0.055	30	437 ± 148 nm
2		0.045	30	425 ± 55 nm
3		0.035	30	306 ± 114 nm
4	1:12.3	0.045	30	238 ± 78 nm
5		0.035	30	321 ± 65 nm
6		0.045	28	406 ± 115 nm
7		0.045	29	510 ± 126 nm
8		0.018	25	459 ± 320 nm
9		0.016	25	416 ± 125 nm
10		0.016	18	336 ± 148 nm

#### 2.6.3 Crosslinking using glutaraldehyde rendered the scaffolds water-insoluble

Upon testing the solubility of the prepared scaffolds in water, the produced fibers were readily soluble upon first contact with an aqueous solution. Supplementing the electrospun polymer mixture with 1.25% or 2% glutaraldehyde rendered them water-insoluble, and gamma-irradiation sterilized the resulting scaffolds. The percentage of glutaraldehyde used affected the final density of electrospun fibers and the ease of detachment from the collector; a lower concentration (1.25%) produced denser fibers that were more easily separated from the collector.

#### 2.6.4 All the produced scaffolds are within the nanoscale range

**FIGURE 7:**
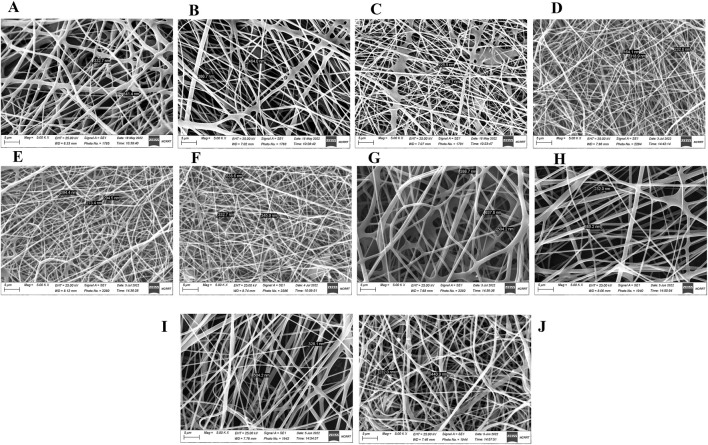
Scanning electron microscopic examination of SA/PVA scaffolds using different running parameters at 5 µm magnitude: **(A)** Scaffold 1, **(B)** Scaffold 2, **(C)** Scaffold 3, **(D)** Scaffold 4 with the smallest fiber mean diameter of 238 ± 78 nm, **(E)** Scaffold 5, **(F)** Scaffold 6, **(G)** Scaffold 7 with the largest mean diameter of 510 ± 126 nm, **(H)** Scaffold 8, **(I)** Scaffold 9, and **(J)** Scaffold 10. All scaffolds were produced by operating the runs for a fixed time interval.

Scanning electron microscopy examination of the irradiated fibers from optimization runs 1–10 (Table 3) is shown in Figure 7. Fibers in all tested scaffolds were within the nanoscale range, without beading, except for scaffolds 6 and 10, which showed beaded fibers. Fibers of non-uniform diameter were found in the prepared scaffolds 1, 2, 8, and 9. Scaffold 3 showed botches of unspun polymer, and scaffold 7 had the largest diameter. Although the 1:20 SA/PVA formulation, electrospun at a flow rate of 0.035 mm/min and an applied voltage of 30 kV (scaffold 3), had a small mean diameter and a high fiber productivity score, it showed botches of unspun polymer. The 1:12.3 SA/PVA formulation, electrospun using a flow rate of 0.045 mm/min, an applied voltage of 30 kV, and 12.5 cm from the collector (scaffold 4), produced fibers with the smallest mean diameter, along with a high productivity, and a marked stability in the cone jet (Table 3; Figure 7).

#### 2.6.5 Changing the final sodium alginate content affects the fibers’ swelling capacity

**FIGURE 8:**
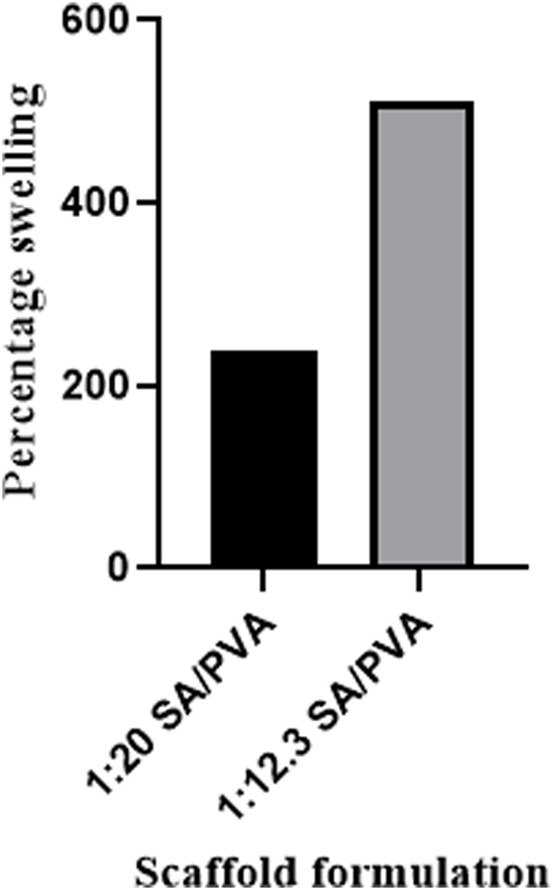
Percentage swelling of the prepared sodium alginate/polyvinyl alcohol (SA/PVA) scaffolds containing different sodium alginate concentrations: The 1:20 SA/PVA formulation with 2.82% SA resulted in 239% swelling, while increasing the SA concentration in the 1:12.3 formulation to 4.2% increases the percentage swelling to 510%.

The scaffold’s ability to absorb water was determined by measuring the percentage swelling after overnight incubation in distilled water at room temperature. Scaffold 4 (1:12.3 SA/PVA) showed 510% swelling, while scaffold 3 (1:20 SA/PVA) showed 239% swelling (Figure 8).

### 2.7 Successful loading of colistin and EM86 onto sodium alginate/polyvinyl alcohol electrospun nanofibers by physical adsorption

**FIGURE 9:**
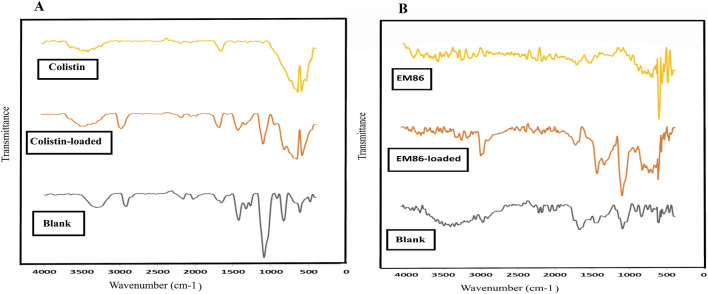
Fourier Transform Infrared spectroscopy charts of colistin and EM86-loaded fibers compared to unloaded blank fibers: **(A)** Colistin-loaded fibers with a broader peak in the range of 3,300–3,500 cm^−1^, corresponding to (OH) hydroxyl groups, and a more prominent peak at 1,650 cm^−1^, corresponding to amide C=O, compared to blank confirmed colistin-loading through physical adsorption technique that involved hydrogen bond formation; **(B)** EM86-loaded-fibers displayed a change in the shape and intensity of the peaks at 3,000 cm^−1^ and 1,650 cm^−1^ compared to blank fibers confirming EM86 loading.

The amount of EM86 and colistin loaded onto gamma-irradiated SA/PVA electrospun nanofibrous wound dressings by physical adsorption was determined using the Bradford assay. An initial peptide concentration of 250 μg/mL resulted in an effective adsorption of 72 µg ± 15 of colistin and 40 µg ± 16 of EM86. Fourier Transform Infrared spectroscopy (FT-IR) was used to confirm loading. The FT-IR results for peptide-loaded and unloaded (blank) scaffolds revealed the characteristic SA and PVA bands, with peak intensities proportional to their blending ratios (Caykara and Demirci, 2006; Kharazmi et al., 2015). Glutaraldehyde crosslinking was confirmed by the CH2- rocking vibration band at 916 cm^−1^ (Jadbabaei et al., 2021). Colistin and EM86 showed a characteristic amide band at 1,650 cm^−1^, with a relatively small peak, attributed to the low peptide concentration (250 μg/mL). Colistin-loaded fibers showed a broader peak at 3,300–3,500 cm^−1^, corresponding to (OH) hydroxyl groups, and a more prominent peak at 1,650 cm^-1^, corresponding to amide C=O, compared to blank, confirming physical adsorption via hydrogen bonding. EM86-loaded fibers displayed a change in the shape and intensity of the peaks at 3,000 cm^-1^ and 1,650 cm^-1^ compared to blank fibers, confirming loading (Figure 9).

### 2.8 EM86-loaded nanofibers had a contact killing effect on *Pseudomonas aeruginosa* SM016

**FIGURE 10:**
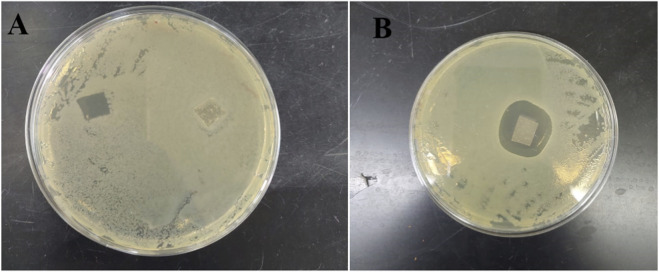
Antibacterial activity of gamma-irradiated SA/PVA electrospun peptide-loaded fibers against *Pseudomonas aeruginosa* SM016: **(A)** Fibers loaded with 45 ± 14 EM86 showed a contact killing effect against *Pseudomonas aeruginosa* SM016; **(B)** fibers loaded with 70 ± 15 colistin displayed a killing effect against *Pseudomonas aeruginosa* SM016.

Gamma-irradiated electrospun SA/PVA nanofibrous dressings, loaded with 45 ± 14 µg EM86 and 70 ± 15 µg colistin, were effective against *Pseudomonas aeruginosa* SM016 (Figure 10).

### 2.9 EM86-loaded SA/PVA electrospun nanofibrous dressings eradicated *Pseudomonas aeruginosa* SM016 in open infected wounds in BALB/c mice

**FIGURE 11:**
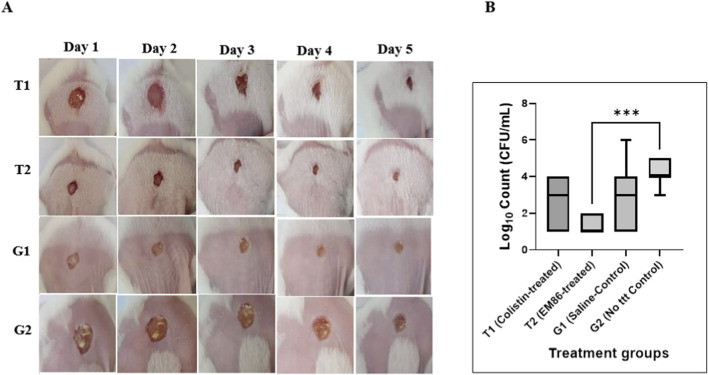
Treatment of *Pseudomonas aeruginosa* SM016 open infected wounds in BALB/c mice with colistin-loaded and EM86-loaded electrospun fibers: **(A)** Representative skin images of *Pseudomonas aeruginosa*-infected wounds in the groups of BALB/c mice receiving different treatments. Images were captured throughout the experiment, spanning from day 1 to day 5. The groups were as follows: (T1) treated with colistin-loaded electrospun fibers, (T2) treated with EM86-loaded fibers, (G1) receiving saline-soaked fibers, and (G2) untreated control group. Day 1 images were taken before application of the target dressings in the designated treatment groups, and day 5 images were captured 24 h after the last treatment; **(B)** A 1 cm^2^ wound area excised on day 5 was homogenized in 2 mL PBS, serially diluted, and spotted on cetrimide agar for viable count. Bacterial log_10_ counts from the different treated groups were plotted, and the detection limit was 10 CFU/mL. Results were analyzed using one-way ANOVA followed by Tukey’s multiple comparisons test, where a significant reduction was recorded in the EM86-treated group (T2) compared to the untreated control group (G2) (*p-*value < 0.001). Error bars represent the standard deviation. *** denotes a *p-*value < 0.001.

Gamma-irradiated SA/PVA electrospun nanofibrous wound dressings loaded with either colistin or EM86 were used to treat *Pseudomonas aeruginosa* SM016 in open infected wounds in BALB/c mice. The dressings used were loaded with either 74 ± 15 µg of colistin or 35 ± 18 µg of EM86. Control groups included a saline-soaked dressing-treated group and an infected-untreated group. The presence of infected wounds was visually confirmed before commencing the treatment protocol in all tested groups. Daily observation of wound progression for each mouse across the various treatment groups was performed (Figure 11A). On day 5, the viable bacterial count in the wound area was assessed (Figure 11B).

Regarding wound-healing progression, colistin-loaded (T1) and EM86-loaded (T2) fibers showed enhanced wound healing, evidenced by wound closure and normal hair regrowth. However, saline-soaked fibers (G1) showed delayed wound healing, and the untreated control (G2) showed increased pus formation until the day of sacrifice (Figure 11A). Wounds in G1 showed better healing than those in G2, with less pus formation by the third day of treatment. The EM86-treated group showed a significant reduction in *Pseudomonas aeruginosa* counts compared to the no-treatment control (*p-*value *<* 0.001; Figure 11B).

## 3 Discussion

Peptide design is revolutionizing drug discovery through computer-aided approaches for the development of AMPs. Specialized AMP databases predict different physical and chemical properties, and machine learning algorithms provide insights into AMP membrane interactions. Computational tools effectively predict the peptide secondary structure and its target interactions (Delaunay and Ha-Duong, 2022). Selecting the appropriate wound dressing is crucial for enhanced healing. Gauze dressings, made of cotton, rayon, and polyester, tend to dry and adhere to wounds, resulting in discomfort and injury to the newly formed tissues upon removal (Borda et al., 2016). In this study, we used computer-based approaches to design anti-Gram-negative AMPs. The shortlisted candidates were synthesized and tested against MDR clinical Gram-negative isolates *in vitro*. The potent candidate was loaded onto an electrospun nanofibrous SA/PVA wound dressing and tested *in vivo* against MDR *P. aeruginosa* in open infected wounds in BALB/c mice.

For designing AMPs, the two parent cyclic peptides, colistin (**KTKKKLLKKT)** and polymyxin (**KTKKKFLKKT**), were selected for sequence modifications. The selection of amino acids for incorporation into the AMP sequence is critical to maintain the core-segment hydrophobicity within the target range of 0.4–2. This ensures membrane insertion and restricts peptide activity to bacterial cells (Liu and Deber, 1998; Shao et al., 2019a; Stark et al., 2002). In the current study, we adopted a sequential approach for sequence modification. First, a triplet tryptophan residue (WWW motif) was inserted at the N-terminus of the parent AMPs. Tryptophan imparts the required hydrophobicity to the peptide sequence, thereby enhancing its ability to disrupt membranes (Liu and Deber, 1998). The WWW motif stabilizes the aliphatic–aromatic and aromatic–aromatic interactions, featuring a π configuration with W2 as the horizontal bar, and W1 and W3 as the two legs (Zarena et al., 2017). In addition, peptides with a WWW motif do not oligomerize due to the tryptophan’s bulky, polarized nature, which prevents self-assembly and minimizes aggregation at the hydrophobic surfaces of monomeric random-coiled AMPs (Mant et al., 2019; Schmidtchen et al., 2009). This allows AMPs to cross the outer membrane of Gram-negative bacteria and reach the inner membrane, whose hydrophobicity induces the AMP to adopt an α-helical conformation, which operates via a carpet model mechanism (Zelezetsky et al., 2005; Mant et al., 2019). Thereby enhancing the antibacterial activity of AMPs and selectivity for bacterial over eukaryotic membranes (Shao et al., 2019a; Zarena et al., 2017; Schmidtchen et al., 2009; Shao et al., 2019b). Furthermore, Trp-rich peptides interact with nucleic acids, leading to cell cycle arrest (Shagaghi et al., 2017).

Given that a specific range of hydrophobicity is required for the designed analogs, and a triplet Trp was already inserted at the N-terminus of all analogs, the further use of highly hydrophobic amino acids, identified by the RT-HPLC scale, specifically phenylalanine (F), tryptophan (W), and leucine (L), was excluded (Liu and Deber, 1998). Thus, alanine (A), which has a midrange hydrophobicity, and isoleucine (I), which is the fourth amino acid on the Liu and Deber hydrophobicity scale based on retention time (Liu and Deber, 1998), were used to substitute for F and L in the designed analogs. Our choice of using alanine aligns with previous studies indicating that peptides containing four leucine residues were toxic to mammalian cells, causing 40%–80% hemolysis due to their high hydrophobicity; however, peptides containing four alanine residues were non-toxic up to 320 µM (Yin et al., 2012).

Amino acids that affect the peptide’s ability to adopt an α-helical structure were excluded from use in sequence modification. These include proline (P), methionine (M), and cysteine (C). Proline enhances the flexibility of peptides; proline-rich peptides act by penetrating membranes without destroying them (Di Stasi et al., 2025). Methionine is prone to oxidation, which affects the peptide structure. A non-oxidized methionine supports an α-helical conformation, and upon oxidation, a β-structure is more favorable (Radchenko et al., 2016). Cysteine forms disulfide bridges supporting a β-sheet structure (Felgueiras and Amorim, 2017). The amino acids that form intra-helical hydrogen bonds between the primary and side chains were excluded as well. These include serine (S), threonine (T), tyrosine (Y), glutamine (Q), and asparagine (N) (Ballesteros et al., 2000; Lee et al., 2018; Roy et al., 2011). Serine and threonine form intra-helical hydrogen bonding between their polar side chains, resulting in a small bending angle in the helix, which affects transmembrane interaction (Ballesteros et al., 2000). The phenolic hydroxyl group of tyrosine forms intra-helical hydrogen bonding with the amide backbone, resulting in stacking and self-assembly into cross-β-like fibers (Lee et al., 2018). Both glutamine and asparagine have an additional amide group in their side chains. The presence of even one Q or N in the peptide sequence results in the formation of chains of hydrogen bonding between their side chains, affecting the secondary structure (Roy et al., 2011). A total of six lysine (K) residues were retained in our designed analogs, as lysine maintains the peptide’s cationic properties and enhances its solubility (Szpak, 2011; Wimley and White, 1996). The use of extra positively charged amino acids, such as arginine (R) and histidine (H), or negatively charged amino acids, such as aspartic acid (D) and glutamic acid (E), was excluded from sequence modification to maintain an unchanged net charge. Glycine (G), an integral component of collagen and the most abundant amino acid in collagen triple helices, was selected for sequence modification (Szpak, 2011). The charge distribution of lysine into 3 residues at each terminal, rather than clustering 6 lysine residues at one terminal, minimizes the peptide’s self-aggregation, due to lysine charge repulsion (Yin et al., 2012). Interrupting the continuous nonpolar surface of the peptide by lysine minimizes the peptide’s self-association among the hydrophobic surfaces of monomeric random-coiled AMPs (Mant et al., 2019). In an aqueous environment, monomeric random-coiled AMPs can traverse the outer membrane of Gram-negative bacteria. The interaction between the peptide’s hydrophobic residues and the inner membrane hydrophobic region induces the peptide’s α-helical conformation, facilitating deeper membrane-insertion and subsequent disruption (Zelezetsky et al., 2005; Mant et al., 2019). This supports the chosen arrangement of hydrophilic and hydrophobic residues within the designed analogs, the importance of a discontinuous nonpolar surface, and the charge distribution of lysine.

In our study, sequence modifications yielded 84 possible analogs. Three of which, namely, EM02, EM60, and EM84, had their calculated CSH within the target range (0.4–2). However, only EM02 was hydrophilic, with a positive Wimley-White whole-residue hydrophobicity value (Wimley and White, 1996). Thus, EM02 was selected and further modified by interchanging the positions of the 6th and 10th residues, yielding EM86. Substituting all lysine residues in EM02 with Dab (a shorter version of lysine) yielded EM85. EM02, EM85, and EM86 were suspected to be membrane-active peptides, having CAMPR3 scores exceeding 0.5. A peptide with 6 Dabs on the polar face was previously reported to have the least α-helical structure in aqueous solution (6%) with most of the α-helical structure induced in contact with the inner bacterial membrane (94%), and to typically align parallel to the membrane surface, acting via a carpet model mechanism (Zelezetsky et al., 2005; Mant et al., 2019; Galdiero et al., 2015). However, lysine’s longer side chains can insert more deeply into the bacterial lipid membrane while maintaining its positively charged groups attached to the negatively charged lipid head groups, a phenomenon known as the snorkeling effect (Strandberg and Killian, 2003). The activity of EM85 was similar to that of EM02 against all the tested *Pseudomonas aeruginosa* and *Klebsiella pneumonia* isolates; however, EM85 was less effective against the tested *Acinetobacter baumanni* isolate. This is in contrast with a previous study that reported that replacing all lysine residues with Dab in the BSI-9 peptide resulted in a 4-fold increase in activity (Thomsen et al., 2020).

Given that our designed peptides possess a net charge similar to that of colistin, we anticipated a comparable level of electrostatic interaction with negatively charged bacterial membranes (Poirel et al., 2017; Schmidtchen et al., 2009; Kohn et al., 2018). In our study, PEP-FOLD was used to predict the 3D α-helical conformation of EM02 and EM86. The 3D structure was employed in PPM computation to simulate the peptide’s interaction with the inner bacterial membrane. The inner membrane insertion in Gram-negative bacteria was illustrated by transmembrane secondary structure segments, with 2 and 3 embedded amino acid residues in EM02 and EM86, respectively. The repositioning of amino acids within the peptide affects the depth of penetration into the inner bacterial membrane (Sosiangdi et al., 2023). Interchanging K6 and A10 of EM02 in EM86 resulted in an increase in the tilt angle from 18° ± 15 to 37° ± 16, an increase in the number of embedded residues from 2 to 3, and an increase in the penetration depth from 4 ± 0.7 to 5.6 ± 1.1 Å. These predictions were confirmed by the lower MIC values for EM86 against the tested Gram-negative bacterial strains than for EM02, except for the tested *Acinetobacter baumannii* isolate.

EM86 showed bactericidal activity comparable to that of colistin against all tested strains with MBC/MIC ratios ≤ 4 (Neethu et al., 2018). The killing kinetics of colistin and EM86 were investigated over 120 min; both peptides achieved maximum killing of *P. aeruginosa* SM016 within 60 min at their 1 × MIC and 2 × MIC. A gradual, consistent reduction in bacterial log_10_ count was observed with 2 × MIC colistin. However, a decrease in bacterial log_10_ count observed after 5 min of treatment with 1 × MIC was followed by an increase at 15 min, then a gradual, consistent reduction. A previous study reported concentration- and time-dependent killing of colistin against *P*. *aeruginosa* PAO1 in its planktonic and biofilm states (Hengzhuang et al., 2011). Using 2 × MIC EM86 resulted in a gradual reduction in the bacterial log_10_ count within the first 15 min of treatment, followed by bacterial regrowth at 30 min, then a sustained reduction. However, the 1 × MIC of EM86 resulted in a consistent reduction in bacterial log_10_ count, with maximum killing at 60 min of treatment. This suggests that EM86 may act via a different mechanism at different concentrations. Using single-cell kinetics and microfluidics-enabled imaging, Zhang et al. reported distinct modes of action for ampicillin and ciprofloxacin at different concentrations against *Escherichia coli* (Zhang et al., 2023). PuroA, a synthetic peptide rich in tryptophan, penetrates the membranes of *Candida albicans* cells without disrupting their integrity, reaching the nucleic acids and binding to them, first causing cell cycle arrest and CFU drop within 10 min before inducing any disruptions into the membrane bilayers. However, killing by pore formation occurs after 40–45 min, achieving complete killing at 60 min (Shagaghi et al., 2017). Another study reported bactericidal activity of 2-fold MIC of AS-hepc3_(41–71)_ and AS-hepc3_(48–56)_ peptides within 30 min and 60 min, respectively, against *Pseudomonas aeruginosa* PAO1 (Zhu et al., 2021). Further investigations regarding the mode of action of EM86 at different concentrations are needed. SEM examination of EM86-treated and untreated *Pseudomonas aeruginosa* SM016 after 30 min at 2 × MIC confirmed the time-kill-kinetics assay results. The cells exposed to the peptide began to lose structural integrity, balloon, burst, and release their cellular contents. This aligns with a previous study showing that treatment of *Pseudomonas aeruginosa* PAO1 with 3 × MIC of the peptides AS-hepc3_(41–71)_ or AS-hepc3_(48–56)_ for 30 min caused morphological changes detected by SEM (Zhu et al., 2021). Comparable results obtained with SEM revealed cell agglutination and clustering of *Escherichia coli* after a 4-h treatment with an eosinophil cationic protein (ECP) (Pulido et al., 2012).

In our study, the decreased activity of a 2-week-old EM86 peptide solution against *P. aeruginosa* SM016 compared with colistin indicates lower peptide stability in solution. The hydrolytic degradation of proteins, along with the cleavage of the amino acid-peptide backbone into fragments, is a typical process that occurs within a timeframe of 2 weeks or less (Pirota et al., 2025). The sulfate salt form of colistin enhanced its stability; salt coupling to the peptides’ N-terminal basic functional group or to the basic residues of side chains forms a neutral salt that stabilizes the peptide and prevents degradation during storage and in solution, decreasing the rate of peptide-backbone cleavage (Laidler and Farkas, 2017). Recent studies highlighted the role of Cu^2+^ and Zn^2+^ metal-ion coupling to the N-terminus of peptide in protecting the peptide-backbone from hydrolytic degradation for approximately 2 weeks in solution. Peptide stability conferred by metal ions is not only attributed to the chemical instability of free peptides, but to structural conformational changes of the peptide induced by the metal ion as well (Pirota et al., 2025). However, Cu^2+^ and Zn^2+^ ions can also contribute to peptide hydrolysis in some cases (Wezynfeld et al., 2016). Enhancing the stability on our designed peptides in solution through salt formation or metal-ion coupling, needs further investigation.

EM86 demonstrated bactericidal activity against *P. aeruginosa* SM016, without inducing cytotoxicity to HSF (IC50 > 300 μg/mL). This aligns with previous research that emphasized the role of Trp-end tags in improving the selectivity of AMPs for Gram-negative phospholipid membranes over eukaryotic membranes, as the incorporation of Trp-tags into cholesterol-rich eukaryotic membranes requires substantial energy (Schmidtchen et al., 2009). A colistin sulfate concentration of 34 μg/mL is the highest safe concentration on HSF proliferation and viability (Quiñones-Vico et al., 2024). Other studies reported an IC50 of 5,900 μg/mL for colistin methane on HSFs (Damour et al., 1992). Our findings confirm that rational peptide design can successfully produce effective AMPs with reduced toxicity.

During fiber preparation, the SA solution alone is nearly impossible to electrospin due to its polyelectrolyte nature, contributing to its high conductivity and lack of chain entanglement. In addition to gelation at low concentrations, the high surface tension of SA contributes to its low spinnability (Taemeh et al., 2020). The hydrophilic polymer (PVA) is used as a carrier to improve the electrospinning of SA. Both SA and PVA interact via hydrogen bonding, thereby reducing the strong inter- and intramolecular SA networks (Shalumon et al., 2011). Thus, the SA solution’s viscosity, surface tension, and conductivity are reduced, and its spinnability is improved (Mokhena et al., 2020).

Our findings support previous studies on the effect of varying flow rates at a constant voltage on the produced fibers. Lowering the flow rate at a constant voltage increases the frequency of receded jets, which are replaced by new cone jets, producing fibers with a wide range of diameters (Zargham et al., 2012). Regardless of the applied voltage, an excessive reduction in flow rate results in low-density fibers, with a narrower fiber distribution, making separation from the collector nearly impossible (Zargham et al., 2012). On the other hand, exceeding the optimal flow rate at a constant voltage results in unspun droplets (Haider et al., 2018), producing integrated fibers with larger mean diameters and more beading. This is attributed to insufficient time for solvent evaporation (Matthews et al., 2002), reduced time for the polymer to reach the collector, and dry up (Ziyadi et al., 2021). Fibers are only produced when the voltage exceeds a threshold (Bagbi et al., 2019). A voltage between 12.5 and 24 kV produced sodium alginate/polyethylene oxide (SA/PEO) nanofibers (Hu et al., 2015). In our study, varying the applied voltage at a constant flow rate resulted in changes in the fibers’ mean diameter: the lowest mean diameter was obtained at 30 kV, a higher mean diameter at 29 kV, and beaded fibers at 28 kV. The effect of varying the applied voltage on fiber diameter is controversial; some studies reported the production of thinner fibers with increasing the voltage at a constant flow rate (Beac et al., 2009), which may be attributed to increased jet instability and stretching (Sener et al., 2011). Other studies reported the production of fibers with larger diameters at higher voltages and constant flow rates (Zhang et al., 2005).

Polymer concentration affects the solution viscosity and influences the morphology and diameter of the produced fibers (Pillay et al., 2013). Increasing the polymer solution concentration increases its viscosity and enhances polymer chain entanglement, thereby counteracting the surface tension and promoting the production of uniform, beadless electrospun nanofibers (Haider et al., 2018). Low polymer concentration leads to electrospraying, and as the polymer solution concentration increases, fiber morphologies evolve from beads with primitive fibers to beaded fibers, then to uniform beadless fibers, and finally to globular fibers. Beaded fibers are produced at a critical chain entanglement concentration (one entanglement/chain), while uniform beadless fibers necessitate a concentration double that of the critical entanglement concentration (2.5 entanglements/chain) (Shenoy et al., 2005). Higher concentrations increase fiber diameter and solution cohesiveness, potentially leading to drying at the needle tip, particularly when the running parameters are incompatible with the formulation, as observed with the (1:15.3) SA/PVA formulation. Some of our SA/PVA formulations (1:6.5 and 1:10) failed electrospinning; their low concentration and viscosity led to polymer chain fragmentation due to competing electric-field and surface-tension forces. (Haider et al., 2018). In contrast, the 1:12.3 SA/PVA formulation produced more uniform beadless fibers with a smaller mean diameter than those from the 1:20 SA/PVA formulation, using the same running parameters. Solution conductivity affects the electrospinning process, impacting Taylor cone formation and fiber diameter (Haider et al., 2018). When the polymer droplet reaches a critical size, its surface charge increases, and the repulsive forces of surface charge counteract and overcome the forces of surface tension, resulting in the formation of a Taylor cone and the production of thinner fibers (Sun et al., 2014). However, an excessive increase in conductivity hinders spinning (Haider et al., 2018). In our study, the 1:12.3 SA/PVA scaffolds containing 4.2% SA exhibited a higher percentage of swelling than the 1:20 SA/PVA scaffolds containing 2.82% SA, due to the higher SA content in the former. SA contributes to the dressing’s swelling by absorbing more water, thereby absorbing wound exudates, preventing wound bed dehydration, facilitating easy removal without sticking, protecting newly formed tissues, and promoting better healing (Coşkun et al., 2014).

Glutaraldehyde is commonly used to crosslink polymers containing hydroxyl groups (Senthil et al., 2024). It forms a covalent bond between the OH groups of starch and PVA, acting as a bridge between them, reducing their aqueous solubility and improving their tensile strength (Musa and Hameed, 2021). In our study, the percentage of glutaraldehyde used to crosslink SA and PVA affected fiber density and the ease of separating the produced fibers from the collector. A high glutaraldehyde concentration (2%) resulted in less dense fibers. This result is consistent with previous studies suggesting that it may be due to a decline in elongation at break, which increases polymer chain stiffness and resistance to stretch (Musa and Hameed, 2021; Chigondo et al., 2013). Using 1.25% glutaraldehyde resulted in better crosslinking of SA/PVA, as reported previously (Chigondo et al., 2013). Dextrose-crosslinked glutaraldehyde hydrogels, prepared with 2.5% glutaraldehyde, are a potential and effective drug-delivery system for wound-healing applications (Senthil et al., 2024). Gamma-irradiation of the produced scaffolds ionizes the polymeric chains via a free-radical mechanism, leading to crosslinking and enhancing the mechanical strength (Naikwadi et al., 2022). In our study, gamma-irradiation at 25 kGy was used to sterilize the water-insoluble glutaraldehyde-containing SA/PVA electrospun scaffolds and to increase their mechanical strength; however, the effect of gamma-irradiation on increasing fiber crosslinking was not studied. The easy detachment of the electrospun scaffolds from the collector confirmed their mechanical strength.

Colistin and EM86 were individually loaded to gamma-irradiated SA/PVA electrospun nanofibrous dressings, with a higher amount of colistin-loaded than EM86, likely due to the sulfate salt form of colistin used. Physical adsorption of colistin and EM86 to the dressings by hydrogen bonding between the peptides’ -NH2 group and the–OH of PVA was evidenced by shifts in the C=O stretching of the primary amide (1,650–1,690) in FT-IR. Colistin-loaded fibers inhibited *Pseudomonas aeruginosa* SM016 with a distinct inhibition zone, indicating colistin diffusion, whereas EM86-loaded fibers exhibited a contact-killing effect. The larger inhibition zone diameter produced by the colistin-loaded fiber can be attributed to the greater amount of colistin loaded (70 µg) compared to EM86 (45 µg). The salting out and diffusion of colistin from its loaded fiber may be attributed to the sulfate salt form used. Salt influences protein-polymer interactions; a previous study reported an increase in the adsorption of transferrin at a nylon interface under high-salt concentrations, attributed to electrostatic shielding, justifying the higher amount of colistin-loaded in our study, and the enhanced desorption in high-salt environments due to structural alterations of transferrin induced by the surface, justifying the increased colistin diffusion from its loaded fibers (Moringo et al., 2019).

Application of an EM86-loaded dressing for 4 days resulted in a significant reduction in *P. aeruginosa* SM016 counts in open-wound infections in BALB/c mice (*p*-value < 0.001), with normal hair regrowth in 71% of mice. Applying a colistin-loaded dressing for 4 days resulted in a non-significant reduction in *P*. *aeruginosa* SM016 counts (*p*-value = 0.0573). This may be due to discrepancies in the MIC values of colistin against *P. aeruginosa* SM016, as determined by the BMD (2 μg/mL, susceptible) and Vitek-2 (≥16 μg/mL, resistant). Discrepancies in the colistin sensitivity results between Vitek-2 and BMD have long been reported (Ananda et al., 2024). The use of saline-soaked fibers for 4 days resulted in a non-significant reduction in *P. aeruginosa* SM016 counts (*p*-value = 0.2995), indicating that SA/PVA dressings can assist in reducing infection in open wounds without drug incorporation. This aligns with studies that highlighted how SA and PVA blends enhance the physical, mechanical, and biological properties of wound healing (Saraiva et al., 2023). The control (untreated) group showed a reduction in bacterial counts compared to the count used to establish the infection. However, none of the mice cleared the infection, as *P. aeruginosa* infection can affect repair processes, leading to chronic wounds (Rufflin and Brochiero, 2019). The decrease in bacterial counts of this group may be attributed to the immune system’s response to infection (Sweere et al., 2019). Wound healing is a complex process involving several stages that extend from a few hours to 1 year from the onset of injury. The hemostasis stage, during which platelets are activated and blood clots are formed, occurs a few hours after injury to stop bleeding. An inflammation stage then follows, lasting up to 3 days. Afterwards, a proliferation stage extends up to 21 days, followed by re-epithelialization and remodeling that can take up to 1 year (Landén et al., 2016). EM86 reduced inflammation, as evidenced by a visual decrease in redness and heat in the infected wounds of EM86-treated groups compared with the control groups. However, extensive research on the effects of EM86 on cellular stages of wound healing is still required.

## 4 Materials and methods

### 4.1 Peptides

#### 4.1.1 Mining the AMP database (ADP3), database filtering, and designing analogs

The APD3 was used to search for AMPs with anti-Gram-negative, antibiofilm, and wound-healing properties (https://aps.unmc.edu/AP/; accessed in 2019). Clustal Omega, from the European Bioinformatics Institute, was used for multiple sequence alignment of the anti-Gram-negative AMPs, the anti-Gram-negative and antibiofilm AMPs, and the AMPs with wound healing activities (https://www.ebi.ac.uk/jdispatcher/msa/clustalo). Two AMPs with anti-Gram-negative and antibiofilm activities, colistin and polymyxin, were selected for sequence modification. The sequences were modified to achieve a helical conformation, broaden their activity spectrum, and reduce toxicity while maintaining the hydrophobicity between the first and second thresholds (Sener et al., 2011).

A sequential design approach was adopted. First, a WWW motif was inserted as an N-terminus tag to all sequences (Supplementary Table S4), followed by altering the type and position of amino acids in the parent AMPs. The antimicrobial peptide calculator and predictor at APD3 was used to calculate: the peptide hydrophobicity, the ability to form α helices, the number of hydrophobic residues on the same surface, percentage of each amino acid, the Boman index, the Wimley-White whole residue hydrophobicity, the molecular weight, the molecular formula, and the net charge for each modified sequence (https://aps.unmc.edu/prediction). CSH was calculated according to the method of Liu and Deber (1998). The membrane activity of the modified sequences was predicted using machine learning algorithms, including support vector machine (SVM), random forest (RF), artificial neural network (ANN), and discriminant analysis (DA), using the CAMPR3 database (http://www.camp.bicnirrh.res.in/predict/). Peptides are expected to exhibit antimicrobial activity if their calculated score exceeds 0.5.

The modified sequences were shortlisted based on the core-segment hydrophobicity and the ability to form an alpha helix. According to APD3, peptides with higher hydrophobicity typically have more negative Wimley-White values (Wimley and White, 1996). The selected analog, EM02, was further modified. Substituting lysine residues in EM02 with Dab and interchanging K6 and A10 of EM02 produced two additional sequences, EM85 and EM86, respectively. Helical wheel projection was used to predict the spatial arrangement of the amino acids in EM02, EM85, and EM86 (http://lbqp.unb.br/NetWheels/) (Sosiangdi et al., 2023). PEP-FOLD 3 was used to predict the 3D fold structures of EM02 and EM86 (https://mobyle.rpbs.univ-paris-diderot.fr/cgi-bin/portal.py#forms::PEP-FOLD3), which were further used to predict the peptide-positioning in membrane (PPM), and illustrate the peptide interaction with the bacterial membrane (https://opm.phar.umich.edu/ppm_server3_cgopm) (Sosiangdi et al., 2023).

#### 4.1.2 Peptide synthesis and purification

EM02, EM85, and EM86 were synthesized and analyzed by China Peptides Co., Ltd (China). The purity of the peptides was confirmed to be greater than 95% by analytical reverse-phase high-performance liquid chromatography (RP-HPLC). Colistin sulfate (20,500 IU/mg) was provided by VACCERA, Egypt. All used peptides were dissolved in deionized water before use.

### 4.2 Bacterial strains

Multidrug-resistant *Pseudomonas aeruginosa* wound isolates SM012, SM014, and SM016, *Acinetobacter baumanni complex* wound isolate SM008, and *Klebsiella pneumoniae* wound isolate SM022 from the culture collection of the Department of Microbiology and Immunology, Faculty of Pharmacy, Cairo University, were used. They were identified by the Vitek-2 compact system model no. VK2C24071, Biomerieux, France, using the Gram-negative ID card (Vitek2 GN21341), and antimicrobial susceptibility testing was determined using the Gram-negative susceptibility card for non-fermenters (Vitek 2 AST-N222), and the Gram-negative susceptibility card for Enterobacteriaceae (Vitek 2 ASTN 73). Cultures were grown on Muller-Hinton agar plates (MHA, Conda, India) and incubated overnight at 37 °C. For long-term storage, isolates were stored in Muller-Hinton broth (MHB, Conda, India) containing 20% glycerol (Piochem, 99% PH Eur, BP) at − 80 °C (Islam, 2020).

### 4.3 Testing the antimicrobial activity of the designed peptides

#### 4.3.1 Determination of the minimum inhibitory concentration and minimum bactericidal concentration of peptides using the broth microdilution method

Antimicrobial susceptibility testing for the designed analogs and their parent peptide, colistin, was performed according to the CLSI 2020 guidelines (CLSI, 2020). Stock solutions of colistin sulfate, EM02, EM85, and EM86 were prepared at a final concentration of 1 mg/mL in sterile deionized water. Fresh colonies were suspended in saline to prepare an inoculum of 1.5 × 10^8^ CFU/mL (0.5 MacFarland standard), then diluted 1:75. A volume of 25 μL of the tested peptide dilution, 25 μL of the diluted bacterial suspension, and 50 μL MHB were added to the wells of a round-bottom 96-well plate to achieve a final bacterial concentration of 5 × 10^5^ CFU/mL. Positive and negative control wells were included. The plates were incubated for 18–24 h at 37 °C. The MIC was determined for freshly prepared and 2-week-old peptide solutions stored at − 20 °C. The lowest peptide concentration that inhibited growth was considered the MIC. The experiments were repeated three times independently.

The MBC was determined by the BMD method according to the CLSI guidelines (CLSI, 1999). Aliquots of 50 μL, taken from the wells showing absence of growth in the MIC experiment, were streaked onto Brain Heart Infusion (BHI, LabM, USA) agar plates and incubated for 24 h at 35 °C. MBC is the lowest peptide concentration that results in complete bacterial eradication. The experiments were repeated three times independently.

#### 4.3.2 Analysis of the time-killing kinetics of EM86 and colistin

A time-kill assay was used to evaluate the bactericidal kinetics of EM86 and colistin against *Pseudomonas aeruginosa* SM016. Fresh colonies were suspended in saline to prepare an inoculum of 1.5 × 10^8^ CFU/mL (0.5 MacFarland standard), then diluted 1:75. A 150 µL volume of the adjusted inoculum was added to 300 μL MHB, and 150 µL of either 1× MIC or 2× MIC of the tested peptide was added. The treated samples were incubated at 37 °C for 2 h. An untreated control without the peptide was prepared. Aliquots of 50 µL were taken at different time intervals (5, 15, 30, 60, and 120 min), and the viable count was determined (Zhu et al., 2021). The experiment was performed in triplicate.

#### 4.3.3 Examination of EM86-treated *Pseudomonas aeruginosa* SM016 by the scanning electron microscope

*Pseudomonas aeruginosa* SM016 was grown to the logarithmic phase, centrifuged, and the pellets collected and washed 3 times with Phosphate-Buffered Saline (PBS, pH 7.4). The pellets were then resuspended in PBS to a concentration of 5 × 10^8^ CFU/mL, followed by treatment with 2 × MIC of EM86 and incubation at 37 °C for 30 min. A no-treatment control was included. Bacterial pellets were collected by spinning down and washed 3 times with PBS. This was followed by fixation with 2% glutaraldehyde, dehydration in serial grades of ethanol (30, 50, 70, 100), gold coating, and examination by SEM (ZEISS EVO15, UK), according to the manufacturer’s instructions (Zhu et al., 2021).

### 4.4 Evaluation of the cytotoxicity of EM86 to human skin fibroblasts

Human Skin Fibroblast cells (R-SO19843)-1 were obtained from Nawah Scientific Inc. (Cairo, Egypt). Cells were grown in Dulbecco’s Modified Eagle Medium (DMEM) supplemented with 100 mg/mL streptomycin, 100 units/mL penicillin, and 10% heat-inactivated fetal bovine serum, maintained in a humidified environment with 5% (v/v) CO_2_ at 37 °C. Cell viability was assessed using the 3-(4,5-dimethylthiazol-2-yl)-2,5-diphenyltetrazolium bromide (MTT) assay. A total of 100 µL aliquots of a cell suspension, 5 × 10^3^ cells, were plated in 96-well plates and incubated in complete media for 24 h at 37 °C in a 5% CO_2_ atmosphere. Cells were treated with aliquots of 100 µL of media containing varying EM86 concentrations (0.03–300) µg/mL for 48 h. Following treatment, the supernatant was removed, and 20 µL of MTT solution (1 mg/mL) was added to each well, followed by incubation for 4 h at 37 °C. The formed formazan crystals were dissolved in 100 µL DMSO, and the absorbance was measured at 570 nm using a multi-well plate reader (BMGLABTECH® FLUOstar Omega, Germany) (Mosmann, 1983; Ahmed et al., 2022).

### 4.5 Preparation and characterization of the electrospun SA/PVA dressing

Electrospun-based SA scaffolds were fabricated by optimizing several formulation and process variables, such as SA:PVA ratio (1:6.5–1:20), flow rate (Q) (0.015–0.075 mm/min), voltage (V) (18–30 kV), and distance to the collector (X) (fixed at 12.5 cm) and testing one variable at a time (Supplementary Table S6). PVA (m.wt. approx. 115000, Loba Chemie, India) polymer solutions (13% and 15% w/v) were prepared by dissolving PVA in deionized water at 80 °C with magnetic stirring until fully dissolved (2 h) (Musa and Hameed, 2021; Kusumastuti et al., 2016). Solutions of 2% and 5% w/v SA (high-molecular-weight, Thomas Baker Pvt. Ltd., India) were prepared in deionized water. Formulas with different SA:PVA ratios were prepared by mixing the required volumes of each polymer under magnetic stirring, followed by sonication for 10 min at 170 W (Jadbabaei et al., 2021). The polymer mixture was electrospun using the NEU nanofiber electrospinning unit (Kato Tech CO., Ltd., Japan), according to the manufacturer’s instructions.

The solubility of the produced fibers in water was tested by immersing a 5 cm^2^ scaffold in 10 mL of distilled water. The effect of incorporating glutaraldehyde into the electrospinning polymer solution on fiber solubility was tested. Different concentrations (1.25% or 2%) of glutaraldehyde (25%, MERCK-Schuchardt, Germany) were added to SA/PVA mixtures, stirred for 30 min with a magnetic stirrer, and sonicated for 10 min at 170 W before electrospinning (Jadbabaei et al., 2021; Musa and Hameed, 2021). The glutaraldehyde-containing polymeric solution was then electrospun under the optimized running conditions. The solubility of the produced scaffolds was tested in water. Glutaraldehyde-containing scaffolds were subjected to gamma-irradiation at 25–50 kGy with a dose rate of 0.905 kGy/h, using a Cobalt-60 (Co 60) Gamma cell 40 source (Canada Co Ltd., Canada), located at NCRRT, to ensure crosslinking and to sterilize the produced mats (Lee et al., 2021).

Morphological characterization of the produced SA/PVA dressings was performed using SEM (ZEISS EVO15, UK), according to the manufacturer’s instructions. To test the ability of the produced scaffolds to absorb fluid exudates. A swelling test was performed. Briefly, the scaffold’s dry weight was determined ( $\left.W0\right)$ , and then incubated overnight in 10 mL of distilled water. Excess fluid was eliminated by patting with a dry tissue. The sample weight was redetermined ( $\left.Wt\right)$ , and the change in weight due to swelling was calculated using the following equation: $\%\;Swelling=\left(\frac{Wt-W0}{W0}\right)\times 100$ (Jadbabaei et al., 2021; Balaji et al., 2016).

### 4.6 Loading peptides to SA/PVA electrospun nanofibrous wound dressing

The gamma-irradiated SA/PVA scaffold was loaded with colistin or EM86 by physical adsorption. The electrospun nanofibers were sectioned into pieces of 1 cm^2^, placed into the wells of a flat bottom 96-well plate, rinsed with sterile distilled, stabilized for 30 min in PBS (pH 7.4) at 37 °C, to ensure the elimination of any residual glutaraldehyde, transferred to the wells of a new plate filled with 300 µL of a 250 μg/mL of the peptide solution (EM86 or colistin), and incubated for 1 h at 37 °C. The amount loaded was calculated using the Bradford assay (Balaji et al., 2016). Bovine serum albumin (BSA, Sigma-Aldrich®, USA) was used as a control for plotting the standard curve, which was employed for the determination of EM86 and colistin concentrations, in wells, by direct reading through their corresponding absorbances (Kielkopf et al., 2020), and the quantity loaded was calculated from the equation:

$$Q=\frac{\left(Ci-Cf\right)\;V}{\text{AT}}$$

Q: quantity of adsorbed peptide (µg), Ci: initial peptide conc. (µg/mL), Cf: final peptide conc. (µg/mL), V: vol. of solution (mL), A: area of scaffold (cm^2^), T: the thickness of scaffold (cm). Cf was determined from the calibration curve (Esfahani and Ghiyasi, 2020).

#### 4.6.1 Examination of peptide-loaded electrospun SA/PVA nanofibrous dressings by Fourier Transform Infrared spectroscopy

Fourier Transform Infrared Spectroscopy analysis was performed to confirm peptide loading. Colistin- and EM86-loaded fibers were analyzed by FT-IR spectroscopy (Model No. VERTEX 70, BRUKER), according to the manufacturer’s instructions. Unloaded blank fibers and peptide solutions (250 μg/mL) were used as controls. Samples were examined using operating wavelengths spanning from 6,000 to 80 cm^−1^.

### 4.7 Testing the antibacterial activity of loaded nanofibers

An inoculum of 1 × 10^7^ CFU/mL of *Pseudomonas aeruginosa* SM016 was surface-inoculated on MHA plates, and the peptide-loaded dressing was applied to the surface. Nanofibers loaded with 70 ± 15 µg of colistin and 45 ± 14 µg of EM86 were used individually; unloaded fibers acted as a negative control. Plates were incubated at 37 °C and examined after 24 and 48 h (Liu et al., 2021). The experiment was performed in triplicate.

### 4.8 Evaluating the activity of peptide-loaded electrospun SA/PVA nanofibrous dressings in a murine skin infection model

Female BALB/c mice (n = 28), aged 6–8 weeks, weighing 20–25 g, purchased from Theodor Bilharz Research Institute, Giza, Egypt, were included in the study. Mice were kept in polycarbonate cages with husk bedding. Temperature and humidity were maintained at 22 ± 2 °C and 60% ± 10%, respectively, under a 12-h light/dark cycle. Food and water were *ad libitum*. The mice were allowed to acclimatize to experimental conditions for 1 week. All procedures complied with the National Research Council’s Guide for the Care and Use of Laboratory Animals (National Research Council, 2011) and were approved by the Research Ethics Committee of the Faculty of Pharmacy, Cairo University, Cairo, Egypt [Approval no: MI (2899)]. Mice were weighed and monitored regularly throughout the experiment.

The backs of the mice were shaved 1 day prior to infection (day − 1). On day 0, mice were anesthetized using intraperitoneal injection of ketamine (90 mg/kg), the skin was rubbed with povidone-iodine, and a 1 cm^2^ full-thickness wound that involved the epidermis and dermis was created on the back of each mouse using a sharp curved surgical scissor (Yang et al., 2019). Mice were then infected by inoculating 1 × 10^6^ CFU of *Pseudomonas aeruginosa* SM016 in 40 μL sterile PBS into each open wound.

Mice were randomly assigned to 4 treatment groups, each containing 7 mice: group 1 (T1) received colistin-loaded dressings, group 2 (T2) received EM86-loaded dressings, group 3 (G1) received saline-soaked fibers, and group 4 (G2) was untreated. After 24 h of infection (day 1), each group was given its designated wound dressings. All mice received a once-daily treatment for 4 days (Yang et al., 2019; Mohamed et al., 2016; Nour et al., 2020). The peptide-loaded dressings were freshly prepared, and the amount loaded was calculated before application to the infected wound. Twenty-four hours following the last treatment (day 5), mice received an anesthetic overdose (5% isoflurane), and cervical dislocation was performed. A 1 cm^2^ wound area was excised and homogenized in 2 mL PBS (Daihan, Korea). The homogenate was serially diluted, and aliquots were spotted onto cetrimide agar (Conda, India) for viable counting. The mice carcasses were stored at − 20 °C until disposal by incineration (Mohamed et al., 2016; Nour et al., 2020).

### 4.9 Statistical analysis

Experiments were performed in triplicate, and mean values with standard deviations were calculated and presented in graphs with error bars. Statistical analysis was conducted using GraphPad Prism 9, employing a one-way ANOVA followed by Tukey’s multiple comparisons test for analyzing the animal model results.

## 5 Conclusion

Using computer-aided approaches, we designed the AMP EM86, a colistin analog with bactericidal activity against MDR Gram-negative microorganisms and reduced toxicity to HSF compared with colistin. We successfully loaded EM86 onto SA/PVA electrospun nanofiber dressing by physical adsorption. The EM86-loaded dressings effectively treated MDR *P. aeruginosa* infections in open wounds of BALB/c mice and protected the wound from injury during daily dressing changes.

Further investigations are needed to determine the mode of action of EM86 at various concentrations, the potential for resistance development, and its safety for parenteral administration. Additionally, future studies should focus on improving EM86 stability and on assessing the long-term effects of EM86-loaded dressings on wound healing. Despite the high costs of peptide and fiber production, our research offers hope for treating colistin-resistant infections in open wounds.

## Data Availability

The original contributions presented in the study are included in the article/Supplementary Material, further inquiries can be directed to the corresponding author/s.

## References

[B1] Aghapour Z. Gholizadeh P. Ganbarov K. Bialvaei A. Z. Mahmood S. S. Tanomand A. (2019). Molecular mechanisms related to colistin resistance in Enterobacteriaceae. Infect. Drug Resist. 12, 965–975. 10.2147/IDR.S199844 31190901 PMC6519339

[B2] Ahmed A. H. H. Mohamed M. F. A. Allam R. M. Nafady A. Mohamed S. K. Gouda A. E. (2022). Design, synthesis, and molecular docking of novel pyrazole-chalcone analogs of lonazolac as 5-LOX, iNOS and tubulin polymerization inhibitors with potential anticancer and anti-inflammatory activities. Bioorg Chem. 129, 106171. 10.1016/j.bioorg.2022.106171 36166898

[B3] AL Tall Y. Abualhaijaa A. Alsaggar M. Almaaytah A. Masadeh M. Alzoubi K. H. (2019). Design and characterization of a new hybrid peptide from LL-37 and BMAP-27. Infect. Drug Resist. 12, 1035–1045. 10.2147/IDR.S199473 31118709 PMC6503343

[B4] Ananda T. Vandana K. E. Mukhopadhyay C. **(** 2024). Comparative evaluation of Vitek®2 and broth microdilution method for colistin susceptibility testing of Gram-negative isolates from intensive care unit in a tertiary care hospital, Indian J. Med. Microbiol. 48, 100559. 10.1016/j.ijmmb.2024.100559 38447856

[B5] Ashrafi B. Chehelcheraghi F. Rashidipour M. Hadavand S. Beiranvand B. Taherikalani M. (2014). Electrospun nanofibrous biocomposite of royal Jelly/Chitosan/Polyvinyl alcohol (RJ/CS/PVA) gel as a biological dressing for P. aeruginosa-infected burn wound. Appl. Biochem. Biotechnol. 196, 3162–3183. 10.1007/s12010-023-04701-9 37632660

[B6] Bagbi Y. Pandey A. Solanki P. R. (2019). Chapter 10 - electrospun nanofibrous filtration membranes for heavy metals and dye removal. Elsevier, 275–288. 10.1016/B978-0-12-813926-4.00015-X

[B7] Balaji A. Jaganathan S. K. Ismail A. F. Rajasekar R. (2016). Fabrication and hemocompatibility assessment of novel polyurethane-based bio-nanofibrous dressing loaded with honey and Carica papaya extract for the management of burn injuries. Int. J. Nanomedicine 11, 4339–4355. 10.2147/IJN.S112265 27621626 PMC5015880

[B8] Ballesteros J. A. Deupi X. Olivella M. Haaksma E. Pardo L. (2000). Serine and threonine residues Bend α-Helices in the χ1=g− conformation. Biophysical J. 79, 2754–2760. 10.1016/S0006-3495(00)76514-3 11053148 PMC1301156

[B9] Barnes M. D. Winkler M. L. Taracila M. A. Page M. G. Desarbre E. Kreiswirth B. N. (2017). Klebsiella pneumoniae Carbapenemase-2 (KPC-2), substitutions at ambler position Asp179, and resistance to ceftazidime-avibactam: unique antibiotic-resistant phenotypes emerge from β-Lactamase protein engineering. Mbio 8, e00528-00517. 10.1128/mBio.00528-17 29089425 PMC5666153

[B10] Beachley V. Wen X. (2009). Effect of electrospinning parameters on the nanofiber diameter and length. J. Mater. Sci. Eng. C 29, 663–668. 10.1016/j.msec.2008.10.037 21461344 PMC3065832

[B11] Borda L. J. Macquhae F. E. Kirsner R. S. (2016). Wound dressings: a comprehensive review. Curr. Derm. Rep. 5, 287–297. 10.1007/s13671-016-0162-5

[B12] Cardoso M. H. Orozco R. Q. Rezende S. B. Rodrigues G. Oshiro K. G. N. Cândido E. S. (2020). Computer-aided design of antimicrobial peptides: are we generating effective drug candidates? Front. Microbiol. 10, 3097. 10.3389/fmicb.2019.03097 32038544 PMC6987251

[B13] Caykara T. Demirci S. (2006). Preparation and characterization of blend films of poly(vinyl alcohol) and sodium alginate. J. Macromol. Sci. Part A Pure Appl. Chem. 43, 1113–1121. 10.1080/10601320600740389

[B14] Chigondo F. Shoko P. Nyamunda B. C. UpenyuGuyo Moyo M. (2013). Maize stalk as reinforcement in natural rubber composites. Int. J. Sci. Tech. Res. 2, 263–271.

[B15] CLSI (2020). Performance standards for antimicrobial susceptibility testing. 30th ed., 40. Wayne, PA: Clinical and Laboratory Standard Institute, 1558–6502.

[B16] CLSI (1999). Methods for determining bactericidal activity of antimicrobial agents. Approved guideline, 19, 18.

[B17] Coşkun G. Karaca E. Ozyurtlu M. Ozbek S. Yermezler A. Cavuşoğlu I. (2014). Histological evaluation of wound healing performance of electrospun poly(vinyl alcohol)/sodium alginate as wound dressing in vivo. Biomed. Mater Eng. 24, 1527–1536. 10.3233/BME-130956 24642979

[B18] Damour O. Zhi Hua S. Lasne F. Villain M. Rousselle P. Collombel C. (1992). Cytotoxicity evaluation of antiseptics and antibiotics on cultured human fibroblasts and keratinocytes. Burns 18, 479–485. 10.1016/0305-4179(92)90180-3 1489497

[B19] Delaunay M. Ha-Duong T. (2022). Computational tools and strategies to develop peptide-based inhibitors of protein-protein interactions. Methods Mol. Biol. 2405, 205–230. 10.1007/978-1-0716-1855-4_11 35298816

[B20] Di Stasi A. Capolla S. Morici M. Bozzer S. Berger M. Pacor S. (2025). Mechanistic divergence and differential antibacterial potency of the proline-rich antimicrobial peptide B7-005 across ESKAPE + E pathogens. Probiotics Antimicro. Prot. 18, 1170–1186. 10.1007/s12602-025-10568-5 40471535 PMC12999614

[B21] Duque Sánchez L. Brack N. Postma A. Pigram P. J. Meagher L. (2016). Surface modification of electrospun fibers for biomedical applications: a focus on radical polymerization methods. Biomaterials 106, 24–45. 10.1016/j.biomaterials.2016.08.011 27543920

[B22] Esfahani H. Ghiyasi Y. (2020). Effect of HA nanoparticles on adsorption of vitamin D3 on super-hydrophobic PA6 nanofibrous scaffold. Rev. Matéria 25, 1. 10.1590/S1517-707620200001.0927

[B23] Felgueiras H. P. Amorim M. T. (2017). Functionalization of electrospun polymeric wound dressings with antimicrobial peptides. Colloids Surf. B Biointerfaces 156, 133–148. 10.1016/j.colsurfb.2017.05.001 28527357

[B24] Fjell C. D. Hiss J. A. Hancock R. E. Schneider G. (2021). Designing antimicrobial peptides: form follows function. Nat. Rev. Drug Discov. 11, 37–51. 10.1038/nrd3591 22173434

[B25] Galdiero S. Falanga A. Berisio R. Grieco P. Morelli G. Galdiero M. (2015). Antimicrobial peptides as an opportunity against bacterial diseases. Curr. Med. Chem. 22, 1665–1677. 10.2174/0929867322666150311145632 25760092

[B26] Gizaw M. Thompson J. Faglie A. Lee S. Y. Neuenschwander P. Chou S. F. (2018). Electrospun fibers as a dressing material for drug and biological agent delivery in wound healing applications. J. Bioeng. 5, 9. 10.3390/bioengineering5010009 29382065 PMC5874875

[B27] Haider A. Haider S. Kang I.-K. (2018). A comprehensive review summarizing the effect of electrospinning parameters and potential applications of nanofibers in biomedical and biotechnology. Arab. J. Chem. 11, 1165–1188. 10.1016/j.arabjc.2015.11.015

[B28] Hancock R. E. W. Diamond G. (2000). The role of cationic antimicrobial peptides in innate host defences. Trends Microbiol. 8, 402–410. 10.1016/S0966-842X(00)01823-0 10989307

[B29] Hengzhuang W. Wu H. Ciofu O. Song Z. Høiby N. (2011). Pharmacokinetics/pharmacodynamics of colistin and imipenem on mucoid and nonmucoid Pseudomonas aeruginosa biofilms. Antimicrob. Agents Chemother. 55, 4469–4474. 10.1128/AAC.00126-11 21670181 PMC3165294

[B30] Hu C. Gong R. H. Zhou F. L. (2015). Electrospun sodium alginate/polyethylene oxide fibers and nano-coated yarns. Int. J. Polym. Sci. 2015, 1–12. 10.1155/2015/126041

[B31] Islam P. **(** 2020 **)**. Creating bacterial glycerol stocks for long-term storage. 10.17504/protocols.io.bf5qjq5w

[B32] Jadbabaei S. Kolahdoozan M. Naeimib F. Dehaghanic H. E. (2021). Preparation and characterization of sodium alginate–PVA polymeric scaffolds by electrospinning method for skin tissue engineering applications. RSC Adv. 11, 30674–30688. 10.1039/d1ra04176b 35479869 PMC9041156

[B33] Jesudason T. (2024). WHO publishes updated list of bacterial priority pathogens. Lancet Microbe 5, 100940. 10.1016/j.lanmic.2024.07.003 39079540

[B34] Karimkhani C. Dellavalle R. P. Coffeng L. E. Flohr C. Hay R. J. Langan S. M. (2017). Global skin disease morbidity and mortality: an update from the global burden of disease study 2013. JAMA Dermatol 153, 406–412. 10.1001/jamadermatol.2016.5538 28249066 PMC5817488

[B35] Kharazmi A. Faraji N. Mat Hussin R. Saion E. Yunus W. M. M. Behzad K. (2015). Structural, optical, opto-thermal and thermal properties of ZnS–PVA nanofluids synthesized through a radiolytic approachBeilstein. J. Nanotechnol. 6, 529–536. 10.3762/bjnano.6.55 25821695 PMC4362026

[B36] Kielkopf C. L. Bauer W. Urbatsch I. L. (2020). Bradford assay for determining protein concentration. Cold Spring Harb. Protoc. 2020, 102269. 10.1101/pdb.prot102269 32238597

[B37] Kim H. S. Yoo H. S. (2010). MMPs-responsive release of DNA from electrospun nanofibrous matrix for local gene therapy: in vitro and in vivo evaluation. J. Control. Release 145, 264–271. 10.1016/j.jconrel.2010.03.006 20347898

[B38] Kofteridis D. P. Valachis A. Koutsounaki E. Maraki S. Mavrogeni E. Economidou F. N. (2012). Skin and soft tissue infections in patients with solid tumours. Sci. World J. 2012, 804518. 10.1100/2012/804518 22448140 PMC3289964

[B39] Kohn E. M. Shirley D. J. Arotsky L. Picciano A. M. Ridgway Z. Urban M. W. (2018). Role of cationic side chains in the antimicrobial activity of C18G. Molecules 23, 329. 10.3390/molecules23020329 29401708 PMC6017431

[B40] Kumar P. Kizhakkedathu J. N. Straus S. K. (2018). Antimicrobial peptides: diversity, mechanism of action and strategies to improve the activity and biocompatibility *in vivo* . Biomolecules 8, 4. 10.3390/biom8010004 29351202 PMC5871973

[B41] Kumar M. Arya D. K. Almujri S. S. Chidambaram K. Pandey P. Kumar A. (2025). Mulberry silk worm pupae oil and prussian blue nanoparticle enriched multi-faceted polyvinyl alcohol nanofiber for infectious full thickness skin wound healing. Tissue Eng. Regen. Med. 22, 1119–1140. 10.1007/s13770-025-00751-8 40974525 PMC12640422

[B42] Kusumastuti Y. Putri N. R. E. Dary A. R. (2016). Electrospinning optimization and characterization of Chitosan/Alginate/polyvinyl alcohol nanofibers. ASTRJ. AIP Conf. Proc. 1755, 150007-1–150007-6. 10.1063/1.4958580

[B43] Laidler P. Farkas I. (2017). Hydrochloride salt of peptide and its use in combination with other peptides for immunotherapy. U.S. Patent and Trademark Office, U.S. Patent No. 9,657,061 B2.

[B44] Landén N. X. Li D. Ståhle M. (2016). Transition from inflammation to proliferation: a critical step during wound healing. Cell. Mol. Life Sci. 73, 3861–3885. 10.1007/s00018-016-2268-0 27180275 PMC5021733

[B45] Lee J. Ju M. Hyun Cho O. Kim Y. Nam K. T. Lee J. (2018). Tyrosine-Rich peptides as a platform for assembly and material synthesis. Adv. Sci. (Weinh) 6, 1801255. 10.1002/advs.201801255 30828522 PMC6382316

[B46] Lee J.-G. Jeong J.-O. Jeong S.-I. Park J.-S. (2021). Radiation-based crosslinking technique for enhanced thermal and mechanical properties of HDPE/EVA/PU blends. Polymers 13, 2832. 10.3390/Polym13162832 34451369 PMC8401421

[B47] Liu L.-P. Deber C. M. (1998). Guidelines for membrane protein engineering derived from de novo designed model peptides. Biopolymers 47, 41–62. 10.1002/(SICI)1097-0282(1998)47:1<41::AID-BIP6>3.0.CO;2-X 9692326

[B48] Liu Q. Ouyang W.-C. Zhou X.-H. Jin T. Wu Z.-W. (2021). Antibacterial activity and drug loading of moxifloxacin-loaded poly(vinyl)/chitosan electrospun nanofibers. Front. Mater. 8, 643428. 10.3389/fmats.2021.643428

[B49] Mahlapuu M. Hakansson J. Ringstad L. Bjorn C. (2016). Antimicrobial peptides: an emerging category of therapeutic agents. Front. Cell. Infect. Microbiol. 6, 194. 10.3389/fcimb.2016.00194 28083516 PMC5186781

[B50] Malik A. Singh C. Tiwari P. Verma D. Mehata A. Vikas (2024). Nanofibers of N,N,N-trimethyl chitosan capped bimetallic nanoparticles: preparation, characterization, wound dressing and in vivo treatment of MDR microbial infection and tracking by optical and photoacoustic imaging. Int. J. Biol. Macromol. 263, 130154. 10.1016/j.ijbiomac.2024.130154 38354928

[B51] Mant C. T. Jiang Z. Gera L. Davis T. Nelson K. L. Bevers S. (2019). De novo designed amphipathic α-helical antimicrobial peptides incorporating Dab and Dap residues on the polar face to treat the gram-negative pathogen, *Acinetobacter baumannii* . J. Med. Chem. 62, 3354–3366. 10.1021/acs.jmedchem.8b01785 30848594 PMC6886721

[B52] Matthews J. A. Wnek G. E. Simpson D. G. Bowlin G. L. (2002). Electrospinning of collagen nanofibers. J. Biol. Macromol. 3, 232–238. 10.1021/bm015533u 11888306

[B53] Miller L. G. Eisenberg D. F. Liu H. Chang C. L. Wang Y. Luthra R. (2015). Incidence of skin and soft tissue infections in ambulatory and inpatient settings, 2005–2010. BMC Infect. Dis. 15, 362. 10.1186/s12879-015-1071-0 26293161 PMC4546168

[B54] Mishra A. K. Choi J. Moon E. Baek K. H. (2018). Tryptophan-rich and proline-rich antimicrobial peptides. Molecules 23, 815. 10.3390/molecules23040815 29614844 PMC6017362

[B55] Mohamed M. F. Abdelkhalek A. Seleem M. N. (2016). Evaluation of short synthetic antimicrobial peptides for treatment of drug-resistant and intracellular Staphylococcus aureus. Sci. Rep. 6, 29707. 10.1038/srep29707 27405275 PMC4942614

[B56] Mokhena T. C. Mochane M. J. Mtibe A. John M. J. Sadiku E. R. Sefadi J. S. (2020). Electrospun alginate nanofibers toward various applications: a review. J. Mater. 13, 934. 10.3390/ma13040934 32093142 PMC7078630

[B57] Moringo N. A. Bishop L. D. C. Shen H. Misiura A. Carrejo N. C. Baiyasi R. (2019). A mechanistic examination of salting out in protein-polymer membrane interactions. Proc. Natl. Acad. Sci. U. S. A. 116, 22938–22945. 10.1073/pnas.1909860116 31659038 PMC6859367

[B58] Mosmann T. (1983). Rapid colorimetric assay for cellular growth and survival: application to proliferation and cytotoxicity assays. J. Immunol. Methods. 65, 55–63. 10.1016/0022-1759(83)90303-4 6606682

[B59] Musa B. H. Hameed N. J. (2021). Effect of crosslinking agent (glutaraldehyde) on the mechanical properties of (PVA/Starch) blend and (PVA/PEG) binary blend films. J. Phys. Conf. Ser. 1795, 012064. 10.1088/1742-6596/1795/1/012064

[B60] Naghavi M. Vollset S. Ikuta K. Swetschinski L. Gray A. Wool E. (2024). Global burden of bacterial antimicrobial resistance 1990–2021: a systematic analysis with forecasts to 2050. Lancet 404, 1199–1226. 10.1016/S0140-6736(24)01867-1 39299261 PMC11718157

[B61] Naikwadi A. T. Sharma B. K. Bhatt K. D. Mahanwar P. A. (2022). Gamma radiation processed polymeric materials for high performance applications: a review. Front. Chem. 10, 837111. 10.3389/fchem.2022.837111 35360545 PMC8964295

[B62] National Research Council (2011). Guide for the care and use of laboratory animals. 8th ed. Washington, DC: The National Academies Press. Available online at: https://grants.nih.gov/grants/olaw/guidefor-the-care-and-use-of-laboratory-animals.pdf (Accessed December 30, 2022).

[B63] Neethu S. Midhun S. J. Radhakrishnan E. K. Jyothis M. (2018). Green synthesized silver nanoparticles by marine endophytic fungus Penicillium polonicum and its antibacterial efficacy against biofilm forming, multidrug-resistant Acinetobacter baumanii. Microb. Pathog. 116, 263–272. 10.1016/j.micpath.2018.01.033 29366864

[B64] Nguyen L. T. Haney E. F. Vogel H. J. (2011). The expanding scope of antimicrobial peptide structures and their modes of action. Trends Biotechnol. 29, 464–472. 10.1016/j.tibtech.2011.05.001 21680034

[B65] Nour E.-D. Elhosseiny N. M. El-Gendy M. A. Mahmoud A. A. Hussein M. M. M. Attia A. S. (2020). A rapid lysostaphin production approach and a convenient novel lysostaphin loaded nano-emulgel; as a sustainable low-cost methicillin-resistant *Staphylococcus aureus* combating platform. Biomol 10, 435. 10.3390/biom10030435 PMC7175171 32178236

[B66] Pachon-Ibanez M. E. Smani Y. Pachon J. Sanchez-Cespedes J. (2017). Perspectives for clinical use of engineered human host defense antimicrobial peptides. FEMS Microbiol. Rev. 41, 323–342. 10.1093/femsre/fux012 28521337 PMC5435762

[B67] Pillay V. Dott C. Choonara Y. E. Tyagi C. Tomar L. Kumar P. (2013). A review of the effect of processing variables on the fabrication of electrospun nanofibers for drug delivery applications. J. Nanomater. 2013, 789289. 10.1155/2013/789289

[B68] Pirota V. Monzani E. Dell’Acqua S. Bacchella C. (2025). Role of copper and zinc ions in the hydrolytic degradation of neurodegeneration-related peptides. Molecules 30, 363. 10.3390/molecules30020363 39860233 PMC11767661

[B69] Poirel L. Jayol A. Nordmann P. (2017). Polymyxins: antibacterial activity susceptibility testing, and resistance mechanisms encoded by plasmids or chromosomes. Clin.Microbiol. Rev. 30, 557–596. 10.1128/CMR.00064-16 28275006 PMC5355641

[B70] Porto W. F. Pires A. S. Franco O. L. (2017). Computational tools for exploring sequence databases as a resource for antimicrobial peptides. Biotechnol. Adv. 35, 337–349. 10.1016/j.biotechadv.2017.02.001 28216008

[B71] Puca V. Marulli R. Z. Grande R. Vitale I. Niro A. Molinaro G. (2021). Microbial species isolated from infected wounds and antimicrobial resistance analysis: data emerging from a three-years retrospective study. J. Antibiot. 10, 1162. 10.3390/antibiotics10101162 34680743 PMC8532735

[B72] Pulido D. Moussaoui M. Andreu D. Nogués M. V. Torrent M. Boix E. (2012). Antimicrobial action and cell agglutination by the eosinophil cationic protein are modulated by the cell wall lipopolysaccharide structure. Antimicrob. Agents Chemother. 56, 2378–2385. 10.1128/AAC.06107-11 22330910 PMC3346588

[B73] Quiñones-Vico M. I. Fernández-González A. Ubago-Rodríguez A. Moll K. Norrby-Teglund A. Svensson M. (2024). Antibiotics against pseudomonas aeruginosa on human skin cell lines: determination of the highest non-cytotoxic concentrations with antibiofilm capacity for wound healing strategies. Pharmaceutics 16, 117. 10.3390/pharmaceutics16010117 38258128 PMC10818945

[B74] Rabanal F. Cajal Y. (2017). Recent advances and perspectives in the design and development of polymyxins. Nat. Prod. Rep. 34, 886–908. 10.1039/c7np00023e 28628170

[B75] Radchenko D. S. Kattge S. Kara S. Ulrich A. S. Afonin S. (2016). Does a methionine-to-norleucine substitution in PGLa influence peptide-membrane interactions? Biochimica Biophysica Acta 1858, 2019–2027. 10.1016/j.bbamem.2016.06.002 27267703

[B76] Reynolds D. Kollef M. (2021). The epidemiology and pathogenesis and treatment of *Pseudomonas aeruginosa* infections: an update. Drugs 81, 2117–2131. 10.1007/s40265-021-01635-6 34743315 PMC8572145

[B77] Rhouma M. Beaudry F. Thériault W. Letellier A. (2016). Colistin in pig production: chemistry, mechanism of antibacterial action, microbial resistance emergence, and one health perspectives. Front. Microbiol. 7, 1789. 10.3389/fmicb.2016.01789 27891118 PMC5104958

[B78] Roy D. Dannenberg J. J. (2011). The effects of regularly spaced glutamine substitutions on alpha-helical peptide structures: a DFT/ONIOM study. Chem. Phys. Lett. 512, 255–257. 10.1016/j.cplett.2011.07.024 21927063 PMC3171806

[B79] Rudrapal M. Khairnar S. J. Jadhav A. G. (2020). Drug Repurposing (DR): an emerging approach in drug discovery. Drug Repurposing—Hypothesis Mol. Asp. Ther. Appl. 10.5772/intechopen.93193

[B80] Rufflin M. Brochiero E. (2019). Repair process impairment in epithelial tissues: major features and potential therapeutic avenues. Front. Cell. Infect. Microbiol. 9, 182. 10.3389/fcimb.2019.00182 31214514 PMC6554286

[B81] Samatham R. Kim K. J. (2006). Electric current as a control variable in the electrospinning process. Polym. Eng. Sci. 46, 954–959. 10.1002/pen.20565

[B82] Saraiva M. M. Campelo M. D. S. Câmara Neto J. F. Lima A. B. N. Silva G. A. Dias A. T. F. F. (2023). Alginate/polyvinyl alcohol films for wound healing: advantages and challenges. J. Biomed. Mater. Res. B Appl. Biomater. 111, 220–233. 10.1002/jbm.b.35146 35959858

[B83] Schmidtchen A. Pasupuleti M. Mörgelin M. Davoudi M. Alenfall J. Chalupka A. (2009). Boosting antimicrobial peptides by hydrophobic oligopeptide end tags. J. Biol. Chem. 284 (26), 17584–17594. 10.1074/jbc.M109.011650 19398550 PMC2719397

[B84] Selvaraj C. Chandra I. Singh S. K. (2022). Artificial intelligence and machine learning approaches for drug design: challenges and opportunities for the pharmaceutical industries. Mol. Divers. 1, 1–21. 10.1007/s11030-021-10326-z 34686947 PMC8536481

[B85] Sener A. G. Altay A. S. Altay F. (2011). “Effect of voltage on morphology of electrospun nanofibers,” in ELECO 2011 - 7th international conference on electrical and electronics engineering, I-324. Available online at: https://www.researchgate.net/publication/261423441 (Accessed July 23, 2020).

[B86] Senthil K. Kalpana R. Kumar V. (2024). Effect of dextrose cross-linked glutaraldehyde hydrogel on wound healing activity. J. Pharm. Bioallied Sci. 16, S1195–S1197. 10.4103/jpbs.jpbs_531_23 38882750 PMC11174273

[B87] Shagaghi N. Bhave M. Palombo E. A. Clayton A. H. A. (2017). Revealing the sequence of interactions of PuroA peptide with Candida albicans cells by live-cell imaging. Sci. Rep. 7, 43542. 10.1038/srep43542 28252014 PMC5333355

[B88] Shalumon K. T. Anulekha K. H. Nair S. V. Nair S. V. Chennazhi K. P. Jayakumar R. (2011). Sodium alginate/poly(vinyl alcohol)/nano ZnO composite nanofibers for antibacterial wound dressings. Int. J. Biol. Macromol. 49, 247–254. 10.1016/j.ijbiomac.2011.04.005 21635916

[B89] Shao C. Li W. Lai Z. Akhtar M. U. Dong N. Shan A. (2019a). Effect of terminal arrangement of tryptophan on biological activity of symmetric α-helix forming peptides. Chem. Biol. Drug Des. 94, 2051–2063. 10.1111/cbdd.13608 31442359

[B90] Shao C. Li W. Tan P. Shan A. Dou X. Ma D. (2019b). Symmetrical modification of minimized dermaseptins to extend the spectrum of antimicrobials with endotoxin neutralization potency. Int. J. Mol. Sci. 20, 1417. 10.3390/ijms20061417 30897850 PMC6470953

[B91] Sharma J. Lizu M. Stewart M. Zygula K. Lu Y. Chauhan R. (2015). Multifunctional nanofibers towards active biomedical therapeutics. Polym. J. 7, 186–219. 10.3390/polym7020186

[B92] Shenoy S. L. Bates W. D. Frisch H. L. Wnek G. E. (2005). Role of chain entanglements on fiber formation during electrospinning of polymer solutions: good solvent, non-specific polymer-polymer interaction limit. Polym. J. 46, 3372–3384. 10.1016/j.polymer.2005.03.011

[B93] Sosiangdi S. Taemaitree L. Tankrathok A. Daduang S. Boonlue S. Klaynongsruang S. (2023). Rational design and characterization of cell-selective antimicrobial peptides based on a bioactive peptide from *Crocodylus siamensis* hemoglobin. Sci. Rep. 13, 16096. 10.1038/s41598-023-43274-9 37752188 PMC10522709

[B94] Stark M. Liu L. P. Deber C. M. (2002). Cationic hydrophobic peptides with antimicrobial activity. Antimicrob. Agents Chemother. 46, 3585–3590. 10.1128/AAC.46.11.3585-3590.2002 12384369 PMC128737

[B95] Strandberg E. Killian J. A. (2003). Snorkeling of lysine side chains in transmembrane helices: how easy can it get? FEBS Lett. 544, 69–73. 10.1016/s0014-5793(03)00475-7 12782292

[B96] Suchithra K. V. Hameed A. Surya S. Mahammad S. Arun A. B. (2025). Dual phage-incorporated electrospun polyvinyl alcohol-eudragit nanofiber matrix for rapid healing of diabetic wound infected by Pseudomonas aeruginosa and Staphylococcus aureus. Drug Deliv. Transl. Res. 15, 1092–1108. 10.1007/s13346-024-01660-4 38980574

[B97] Sun B. Long Y. Z. Zhang H. D. Li M. M. Duvail J. L. Jiang X. Y. (2014). Advances in three-dimensional nanofibrous macrostructures via electrospinning. Prog. Polym. Sci. 39, 862–890. 10.1016/j.progpolymsci.2013.06.002

[B98] Sweere J. M. Ishak H. Sunkari V. Bach M. S. Manasherob R. Yadava K. (2019). The immune response to chronic *Pseudomonas aeruginosa* wound infection in immunocompetent mice. J. Adv. Wound Care 9, 35–47. 10.1089/wound.2019.1039 31903297 PMC6940591

[B99] Szpak P. (2011). Fish bone chemistry and ultrastructure: implications for taphonomy and stable isotope analysis. J. Archaeol. Sci. 38, 3358–3372. 10.1016/j.jas.2011.07.022

[B100] Taemeh M. A. Shiravandi A. Korayem M. A. Daemi H. (2020). Fabrication challenges and trends in biomedical applications of alginate electrospun nanofibers. Carbohydr. Polym. 228, 115419. 10.1016/j.carbpol.2019.115419 31635749

[B101] Thomsen T. T. Mendel H. C. Al-Mansour W. Oddo A. Løbner-Olesen A. Hansen P. R. (2020). Analogues of a cyclic antimicrobial peptide with a flexible linker show promising activity against *Pseudomonas aeruginosa* and Staphylococcus aureus. Antibiotics (Basel) 9, 366. 10.3390/antibiotics907036 32629881 PMC7399811

[B102] Vella V. Derreumaux D. Aris E. Pellegrini M. Contorni M. Scherbakov M. (2024). The incidence of skin and soft tissue infections in the United States and associated healthcare utilization between 2010 and 2020. Open Forum Infect. Dis. 11, ofae267. 10.1093/ofid/ofae267 38835497 PMC11146672

[B103] Vishnepolsky B. Zaalishvili G. Karapetian M. Nasrashvili T. Kuljanishvili N. Gabrielian A. (2019). De Novo Design and in vitro testing of antimicrobial peptides against Gram-negative bacteria. Pharmaceuticals (Basel) 12, 82. 10.3390/ph12020082 31163671 PMC6631481

[B104] Waghu F. H. Joseph S. Ghawali S. Martis E. A. Madan T. Venkatesh K. V. (2018). Designing antibacterial peptides with enhanced killing kinetics. Front. Microbiol. 9, 325. 10.3389/fmicb.2018.00325 29527201 PMC5829097

[B105] Wang G. (2019). The antimicrobial peptide database provides a platform for decoding the design principles of naturally occurring antimicrobial peptides. Protein Sci. 29, 8–18. 10.1002/pro.3702 31361941 PMC6933855

[B106] Wezynfeld N. E. Fra˛czyk T. Bal W. (2016). Metal assisted peptide bond hydrolysis: chemistry, biotechnology and toxicological implications. Coord. Chem. Rev. 327–328, 166–187. 10.1016/j.ccr.2016.02.009 327

[B107] Wimley W. C. White S. H. (1996). Experimentally determined hydrophobicity scale for proteins at membrane interfaces. Nat. Struct. Biol. 3 (10), 842–848. 10.1038/nsb1096-842 8836100

[B108] Yang T. Yang H. Zhen S. J. Huang C. Z. (2015). Hydrogen-bond-mediated in situ fabrication of AgNPs/Agar/PAN electrospun nanofibers as reproducible substrates. ACS Appl. Mater. Interfaces. 7, 1586–1594. 10.1021/am507010q 25546719

[B109] Yang M. Zhang C. Hansen S. A. Mitchell W. J. Zhang M. Z. Zhang S. (2019). Antimicrobial efficacy and toxicity of novel CAMPs against P. aeruginosa infection in a murine skin wound infection model. BMC Microbiol. 19, 293. 10.1186/s12866-019-1657-6 31842727 PMC6915932

[B110] Yin L. M. Edwards M. A. Li J. Yip C. M. Deber C. M. (2012). Roles of hydrophobicity and charge distribution of cationic antimicrobial peptides in peptide-membrane interactions. J. Biol. Chem. 287, 7738–7745. 10.1074/jbc.M111.303602 22253439 PMC3293554

[B111] Zarena D. Mishra B. Lushnikova T. Wang F. Wang G. (2017). The π configuration of the WWW motif of a short Trp-rich peptide is critical for targeting bacterial membranes, disrupting preformed biofilms, and killing methicillin-resistant staphylococcus aureus. Biochemistry 56, 4039–4043. 10.1021/acs.biochem.7b00456 28731688 PMC5603908

[B112] Zargham S. Bazgir S. Tavakoli A. Rashidi A. S. Damerchely R. (2012). The effect of flow rate on morphology and deposition area of electrospun nylon 6 nanofiber. JEFF 7, 42–49. 10.1177/155892501200700414

[B113] Zelezetsky I. Pacor S. Pag U. Papo N. Shai Y. Sahl H. G. (2005). Controlled alteration of the shape and conformational stability of α-helical cell-lytic peptides: effect on mode of action and cell specificity. Biochem. J. 390, 177–188. 10.1042/BJ20042138 15836439 PMC1184573

[B114] Zhang C. Yuan X. Wu L. Han Y. Sheng J. (2005). Study on the morphology of electrospun poly(vinyl alcohol) mats. Eur. Polym. J. 41, 423–432. 10.1016/j.eurpolymj.2004.10.027

[B115] Zhang Y. Kepiro I. Ryadnov M. G. Pagliara S. (2023). Single cell killing kinetics differentiate phenotypic bacterial responses to different antibacterial classes. Microbiol. Spectr. 11, e03667-22. 10.1128/spectrum.03667-22 36651776 PMC9927147

[B116] Zhao Y. Zhang M. Qiu S. Wang J. Peng J. Zhao P. (2016). Antimicrobial activity and stability of the D-amino acid substituted derivatives of antimicrobial peptide polybia-MPI. AMB Expr. 6, 122. 10.1186/s13568-016-0295-8 27900727 PMC5128008

[B117] Zhu D. Chen F. Chen Y.-C. Peng H. Wang K.-J. (2021). The long-term effect of a nine amino-acid antimicrobial peptide as-hepc3(48-56) against *Pseudomonas aeruginosa* with no detectable resistance. Front. Cell. Infect. Microbiol. 11, 752637. 10.3389/fcimb.2021.752637 34676176 PMC8523948

[B118] Ziyadi H. Baghali M. Bagherianfar M. Mehrali F. Majidi R. F. (2021). *A*n investigation of factors affecting the electrospinning of poly (vinyl alcohol)/kefiran composite nanofibers. Adv. Compos. Mater. 4, 768–779. 10.1007/s42114-021-00230-3 33748671 PMC7958938

