# Supplementary material for: The antimicrobial peptide EM86 loaded to gamma-irradiated sodium alginate/polyvinyl alcohol electrospun nanofibrous dressing treated multidrug-resistant Pseudomonas aeruginosa wound infections in BALB/c mice
Source: Front Bioeng Biotechnol. 2026 Apr 7;14:1776154. doi: 10.3389/fbioe.2026.1776154 (PMC13095823; doi:10.3389/fbioe.2026.1776154)
Supplement: Supplementary file 1 [file Table1.docx]

Supplementary Table S1. The sequences, net charge and hydrophobicity percentage of the 358 AMPs with anti-Gram-negative activity

|  | **Peptide name / Class** | **Source** | **Sequence** | **Activity** | **length** | **Net charge** | **Hydrophobicity** |
| --- | --- | --- | --- | --- | --- | --- | --- |
| 1 | Microcin C7 (MccC7, Microcin C/MccC, microcin C51/MccC51, MccC7/C51; class 1 microcins, bacteriocins, Gram-negative bacteria, prokaryotes. Others: MccA; XXamp; BBPe, WXS) | *Escherichia coli* | MRTGNAD | Anti-Gram negative | 7 | 1 | 28% |
| 2 | Colistin (Polymyxin E1 and E2; lactam, XXD; XXL; XXJ; UCSB1a; lipopeptides; nonribosomally synthesized peptide antibiotic; bacteria; BBL; BBMm, prokaryotes) | Paenibacillus polymyxa var. colistinus; Also known as *Bacillus polymyxa* | KTKKKLLKKT | Anti-Gram negative & antibiofilm | 10 | 6 | 20% |
| 3 | Champacyclin (bacteria; prokaryotes. XXC; XXD; UCBB1a) | marine sediments of the Gotland Deep (Baltic Sea), *Streptomyces champavatii* | KIIFLIAI | Anti-Gram negative & antifungal | 8 | 1 | 87% |
| 4 | Balteatide (frogs, amphibians, animals; XXA; UCLL1c) | the skin secretion, wild Peruvian purple-sided leaf frogs, *Phyllomedusa baltea*; South America | LRPAILVRIK | Anti-Gram negative & antifungal | 10 | 3 | 60% |
| 5 | Lacrain (myriapods, arthropods, invertebrates, animals; UCLL1a) | Brazilian centipede, *Scolopendra viridicornis* | RYPAVGYT | Anti-Gram negative | 8 | 1 | 25% |
| 6 | Inverso-CysHHC10 (synthetic; XXA) | artificial | CKRWWKWIRW | Anti-Gram negative | 10 | 5 | 60% |
| 7 | Tet110 (synthetic) | artificial | RWRWWRWRR | Anti-Gram negative | 9 | 5 | 44% |
| 8 | Polymyxin B (aerosporin; lactam, XXD; XXL; XXJ; UCSB1a; lipopeptides; nonribosomally synthesized peptide antibiotic; bacteria; BBL; BBMm, prokaryotes; Variants: Polymyxin B, a mixture) | *Bacillus aerosporus Greer* | KTKKKFLKKT | Anti-Gram negative, antibiofilm & antifungal | 10 | 6 | 20% |
| 9 | Apidaecin IA (Pro-rich; insects, arthropods, invertebrates, animals; BBL; VIHNN) | honeybee, *Apis mellifera*L. | GNNRPVYIPQPRPPHPRI | Anti-Gram negative | 18 | 4 | 16% |
| 10 | Apidaecin IB (Pro-rich; insects, arthropods, invertebrates, animals; Derivatives: Api88; Api137; BBribo) | honeybee, *Apis mellifera*L | GNNRPVYIPQPRPPHPRL | Anti-Gram negative | 18 | 4 | 16% |
| 11 | BACTENECIN 5 (BtBac5; bac5, bac 5, bac-5; Pro-rich; bovine cathelicidin, cattle, ruminant, mammals; animals; BBribo) | Bovine neutrophils,*Bos taurus* | RFRPPIRRPPIRPPFYPPFRPPIRPPIFPPIRPPFRPPLGPFP | Anti-Gram negative | 43 | 9 | 27% |
| 12 | BACTENECIN 7 (bac-7, bac 7; bac7; Pro-rich; bovine cathelicidin, ruminant, mammals; animals; BBL, SeqAR, BBPP; Derivatives: Bac7(1-35); Bac7(1-16), BBribo) | Bovine neutrophils,*Bos taurus* | RRIRPRPPRLPRPRPRPLPFPRPGPRPIPRPLPFPRPGPRPIPRPLPFPRPGPRPIPRPL | Anti-Gram negative | 60 | 17 | 20% |
| 13 | Bombinin-like peptide 1 (XXA, BLP-1, UCLL1c; toad, amphibians, animals) | oriental fire bellied toad/frog, *Bombina orientalis*, Asia | GIGASILSAGKSALKGLAKGLAEHFAN | Anti-Gram negative | 27 | 3 | 48% |
| 14 | Bombinin-like peptide 2 (XXA, BLP-2, UCLL1c; toad, amphibians, animals) | Oriental fire bellied toad/frog, *Bombina orientalis*, Asia | GIGSAILSAGKSALKGLAKGLAEHFAN | Anti-Gram negative | 27 | 4 | 48% |
| 15 | Bombinin-like peptide 3 (XXA, BLP-3, XXA, UCLL1c; toad, amphibians, animals) | Oriental fire bellied toad/frog, *Bombina orientalis*, Asia | GIGAAILSAGKSALKGLAKGLAEHF | Anti-Gram negative | 27 | 3 | 52% |
| 16 | Bombinin-like peptide 4 (XXA, BLP-4, UCLL1c; toad, amphibians, animals) | Oriental fire bellied toad/frog, *Bombina orientalis*, Asia | GIGAAILSAGKSIIKGLANGLAEHF | Anti-Gram negative | 25 | 2 | 52% |
| 17 | RANATUERIN 3 (Ranatuerin-3, XXU; 1S=S, UCSS1a; frog, amphibians, animals) | North American bull frog, *Rana catesbeiana* | GFLDIINKLGKTFAGHMLDKIKCTIGTCPPSP | Anti-Gram negative | 32 | 3 | 40% |
| 18 | RANATUERIN 4 (Ranatuerin-4, frog, amphibians, animals; XXU; 1S=S, UCSS1a) | North American bull frog, *Rana catesbeiana* | FLPFIARLAAKVFPSIICSVTKKC | Anti-Gram negative | 24 | 4 | 62% |
| 19 | GLK-19 (synthetic; Derivatives: GLR-19, ZZH) | Database-aided de novo design using freqently occurring amino acids | GLKKLLGKLLKKLGKLLLK | Anti-Gram negative | 19 | 7 | 47% |
| 20 | Formaecin 1 (glycopeptide; XXG, Pro-rich; insects, ants, BBPP, arthropods, invertebrates, animals) | Red bulldog ant *Myrmecia gulosa* | GRPNPVNNKPTPHPRL | Anti-Gram negative | 16 | 3 | 12% |
| 21 | Formaecin 2 (Pro-rich; insects, ants, BBPP, arthropods, invertebrates, animals) | Red bulldog ant *Myrmecia gulosa* | GRPNPVNTKPTPYPRL | Anti-Gram negative | 16 | 3 | 12% |
| 22 | hBD-26 (human beta-defensin 26, hBD26; UCSS1a; primates, mammals, animals; 3S=S) | *Homo sapiens* | WYVKKCLNDVGICKKKCKPEEMHVKNGWAMCGKGRDCCVPAD | Anti-Gram negative | 42 | 4 | 42% |
| 23 | hBD-27 (human beta-defensin 27, hBD27; UCSS1a; primates, mammals, animals; 3S=S) | *Homo sapiens* | QLKKCWNNYVQGHCRKICRVNEVPEALCENGRYCCLNIKELEAC | Anti-Gram negative | 44 | 2 | 43% |
| 24 | Hepcidin (UCSS1a; fish, animals; 4S=S) | White bass (*Morone chrysops)* X Striped bass (*M. saxatilis)* | GCRFCCNCCPNMSGCGVCCRF | Anti-Gram negative | 21 | 2 | 57% |
| 25 | Apidaecin II (Pro-rich; insects, arthropods, invertebrates, animals) | honeybee, *Apis mellifera* | GNNRPIYIPQPRPPHPRL | Anti-Gram negative | 18 | 3 | 16% |
| 26 | Heliocin (glycopeptide; XXG; XXQ; Pro-rich; insects,arthropods, invertebrates, animals) | owlet moth, *Heliothis virescens* | QRFIHPTYRPPPQPRRPVIMRA | Anti-Gram negative | 22 | 5 | 27% |
| 27 | Japonicin-1 (Japonicin-1Ja; frog, amphibians, animals; XXU; 1S=S, UCSS1a) | Japanese reddish frog, *Rana japonica,* Asia | FFPIGVFCKIFKTC | Anti-Gram negative | 14 | 2 | 64% |
| 28 | Metalnikowin I (Pro-rich; insects, arthropods, invertebrates, animals; BBribo; VIHNN) | Green shield bug *Palomena prasina* | VDKPDYRPRPRPPNM | Anti-Gram negative | 15 | 2 | 13% |
| 29 | Metalnikowin IIA (Pro-rich; insects, arthropods, invertebrates, animals) | Green shield bug *Palomena prasina* | VDKPDYRPRPWPRPN | Anti-Gram negative | 15 | 2 | 13% |
| 30 | Metalnikowin IIB (Pro-rich; insects, arthropods, invertebrates, animals) | Green shield bug*Palomena prasina* | VDKPDYRPRPWPRNMI | Anti-Gram negative | 16 | 2 | 25% |
| 31 | Metalnikowin III (Pro-rich; insects, arthropods, invertebrates, animals) | Green shield bug *Palomena prasina* | VDKPDYRPRPWPRPNM | Anti-Gram negative | 16 | 2 | 18% |
| 32 | Ponericin G7 (antss, insects, arthropods, invertebrates, animals) | Pachycondyla goeldii | GLVDVLGKVGGLIKKLLPG | Anti-Gram negative, Antifungal | 19 | 2 | 47% |
| 33 | Salmocidin 2A (fish, trout, animals) | Rainbow trout *Oncorhynchus mykiss* | SGFVLKGYTKTSQ | Anti-Gram negative | 13 | 2 | 23% |
| 34 | Salmocidin 2B (fish, trout, animals) | Rainbow trout *Oncorhynchus mykiss* | AGFVLKGYTKTSQ | Anti-Gram negative | 13 | 2 | 30% |
| 35 | RANATUERIN 6 (XXA; UCLL1c; frog, amphibians, animals; ZZHa_t) | *Rana catesbeiana,* North America | FISAIASMLGKFL | Anti-Gram negative, Antiviral, Anti-HIV, | 13 | 2 | 69% |
| 36 | RANATUERIN 7 (UCLL1; frog, amphibians, animals) | *Rana catesbeiana,* North America | FLSAIASMLGKFL |  | 13 | 1 | 69% |
| 37 | RANATUERIN 8 (UCLL1; frog, amphibians, animals) | *Rana catesbeiana,* North America | FISAIASFLGKFL |  | 13 | 1 | 69% |
| 38 | RANATUERIN 9 (UCLL1; frog, amphibians, animals; ZZH) | *Rana catesbeiana,* North America | FLFPLITSFLSKVL | Anti-Gram negative, Antiviral, Anti-HIV | 14 | 1 | 64% |
| 39 | Acaloleptin A1 (insects, arthropods, invertebrates, animals) | Udo longicorn beetle *Acalolepta luxuriosa* | SLQPGAPNVNNKDQPWQVSPHISRDDSGNTRTDINVQRHGENNDFEAGWSKVVRGPNKAKPTWHIGGTHRW | Anti-Gram negative | 71 | 2 | 23% |
| 40 | Coleoptericin (insects, arthropods, invertebrates, animals) | *Zophobas atratus* | SLQGGAPNFPQPSQQNGGWQVSPDLGRDDKGNTRGQIEIQNKGKDHDFNAGWGKVIRGPNKAKPTWHVGGTYRR | Anti-Gram negative | 74 | 5 | 21% |
| 41 | XT-2 (UCLL1a; frog, amphibians, animals) | Diploid clawed frog *Silurana tropicalis,* (formerly) Xenopus tropicalis , Africa | GCWSTVLGGLKKFAKGGLEAIVNPK | Anti-Gram negative | 25 | 3 | 44% |
| 42 | XT-4 (UCLL1; frog, amphibians, animals) | Diploid clawed frog*Silurana tropicalis, (formerly) Xenopus tropicalis*, Africa | GVFLDALKKFAKGGMNAVLNPK | Anti-Gram negative | 22 | 3 | 50% |
| 43 | Cyclic L27-11 (protegrin-1-mimetic; synthetic; XXD; XXC; UCBB1a; BBomp) | peptide library screen | TWLKKRRWKKAKPP | Anti-Gram negative | 14 | 7 | 28% |
| 44 | Brevinin-1SA (frog, amphibians, animals; XXU; 1S=S, UCSS1a) | Southern Leopard Frog *Rana sphenocephala,* North America | FLPAIVGAAGQFLPKIFCAISKKC | Anti-Gram negative, Antifungal | 24 | 3 | 62% |
| 45 | Brevinin-1SB (frog, amphibians, animals; XXU; 1S=S, UCSS1a) | Southern Leopard Frog *Rana sphenocephala,* North America | FLPAIVGAAGKFLPKIFCAISKKC | Anti-Gram negative, Antifungal | 24 | 4 | 62% |
| 46 | Brevinin-1SC (frog, amphibians, animals; XXU; 1S=S, UCSS1a) | Southern Leopard Frog *Rana sphenocephala,* North America | FFPIVAGVAGQVLKKIYCTISKKC | Anti-Gram negative, Antifungal | 24 | 4 | 54% |
| 47 | Microcin J25 (MccJ25, formerly microcin 25, Mcc25; Gly-rich; lactam; a class 2 lasso peptide; class 1 microcins, bacteriocins, Gram-negative bacteria, prokaryotes; XXJ; UCSB1a; BBpol; revisited) | *Escherichia coli AY25* | GGAGHVPEYFVGIGTPISFYG | Anti-Gram negative | 21 | -1 | 33% |
| 48 | Adepantin-1 (Automatically Designed Peptide antibacterial 1; synthetic) | computational design | GIGKHVGKALKGLKGLLKGLGES | Anti-Gram negative | 23 | 4 | 34% |
| 49 | Human Calcitermin ( primates, mammals, animals; BBII) | airways, *Homo sapiens* | VAIALKAAHYHTHKE | Anti-Gram negative, Antifungal | 15 | 1 | 46% |
| 50 | Shepherin I (Gly-rich; His-rich; plants) | roots, shepherd's purse, *Capsella bursa-pastoris* | GYGGHGGHGGHGGHGGHGGHGHGGGGHG | Anti-Gram negative, Antifungal | 28 | 8 | 0% |
| 51 | Shepherin II (Gly-rich; His-rich; plants) | roots, shepherd's purse, *Capsella bursa-pastoris* | GYHGGHGGHGGGYNGGGGHGGHGGGYNGGGHHGGGGHG | Anti-Gram negative, Antiviral, Antifungal, Anti-HIV | 38 | 8 | 0% |
| 52 | Ocellatin-F1 (Fallaxin, XXA; UCLL1c; frog, amphibians, animals; Derivatives: FA12) | west Indian mountain chick frog, *Leptodactylus fallax*. Asia | GVVDILKGAAKDIAGHLASKVMNKL | Anti-Gram negative, Antifungal | 25 | 3 | 52% |
| 53 | Ocellatin-1 (XXA, UCLL1c; frog, amphibians, animals) | S. American frog skin, *Leptodactylus ocellatus* | GVVDILKGAGKDLLAHLVGKISEKV | Anti-Gram negative, Hemolytic | 25 | 2 | 48% |
| 54 | Ocellatin-2 (XXA, UCLL1c; frog, amphibians, animals) | Skin, *Leptodactylus ocellatus*, South America | GVLDIFKDAAKQILAHAAEKQI | Anti-Gram negative, Hemolytic | 22 | 1 | 54% |
| 55 | Ocellatin-3 (XXA; UCLL1c; frog, amphibians, animals) | South American frog *Leptodactylus ocellatus* | GVLDILKNAAKNILAHAAEQI | Anti-Gram negative, Hemolytic | 21 | 1 | 57% |
| 56 | Nigrocin-2GRa (frog, amphibians, animals; XXU; 1S=S, UCSS1a) | *Rana graham*i, Asia | GLLSGILGAGKHIVCGLSGLC | Anti-Gram negative | 21 | 1 | 52% |
| 57 | Nigrocin-2GRc (frog, amphibians, animals; XXU; 1S=S, UCSS1a) | *Rana grahami*, Asia | GLLSGILGAGKNIVCGLSGLC | Anti-Gram negative | 21 | 1 | 52% |
| 58 | KR-12 (KR12, a minimal AMP from parent cathelicidin, UCLL1; synthetic; XXA) | natural fragment of LL-37 discovered by NMR | KRIVQRIKDFLR | Anti-Gram negative | 12 | 5 | 41% |
| 59 | Palustrin-1b (frog, amphibians, animals; XXU; 1S=S, UCSS1a) | North America, *Rana palustris* | ALFSILRGLKKLGNMGQAFVNCKIYKKC | Anti-Gram negative | 28 | 6 | 50% |
| 60 | Palustrin-1c (frog, amphibians, animals; XXU; 1S=S, UCSS1a) | North America, *Rana palustris* | ALSILRGLEKLAKMGIALTNCKATKKC | Anti-Gram negative | 27 | 5 | 51% |
| 61 | Palustrin-1d ( frog, amphibians, animals; XXU; 1S=S, UCSS1a) | North America, *Rana palustris* | ALSILKGLEKLAKMGIALTNCKATKKC | Anti-Gram negative | 27 | 5 | 51% |
| 62 | Palustrin-2b (UCLL1a; XXU; 1S=S, frog, amphibians, animals) | North America, *Rana palustris* | GFFSTVKNLATNVAGTVIDTLKCKVTGGCRS | Anti-Gram negative | 31 | 3 | 41% |
| 63 | Palustrin-2c (UCLL1a; XXU; 1S=S, frog, amphibians, animals) | North America, *Rana palustris* | GFLSTVKNLATNVAGTVIDTLKCKVTGGCRS | Anti-Gram negative | 31 | 3 | 41% |
| 64 | Palustrin-3a (frog, amphibians, animals; XXU; 1S=S, UCSS1a) | North America, *Rana palustris* | GIFPKIIGKGIKTGIVNGIKSLVKGVGMKVFKAGLNNIGNTGCNEDEC | Anti-Gram negative | 48 | 4 | 39% |
| 65 | Palustrin-3b (frog, amphibians, animals; XXU; 1S=S, UCSS1a) | North America, *Rana palustris* | GIFPKIIGKGIKTGIVNGIKSLVKGVGMKVFKAGLSNIGNTGCNEDEC | Anti-Gram negative | 48 | 4 | 39% |
| 66 | Ranatuerin-2PLa (frog, amphibians, animals; XXU; 1S=S, UCSS1a) | pickerel frog, *Rana palustris*, North America | GIMDTVKNVAKNLAGQLLDKLKCKITAC | Anti-Gram negative | 28 | 3 | 50% |
| 67 | Ranatuerin-2PLb (frog, amphibians, animals; XXU; 1S=S, UCSS1a) | pickerel frog, *Rana palustris*, North America | GIMDTVKNAAKDLAGQLLDKLKCRITGC | Anti-Gram negative | 28 | 2 | 46% |
| 68 | Ranatuerin-2PLc (frog, amphibians, animals; XXU; 1S=S, UCSS1a) | pickerel frog, *Rana palustris*, North America | GLLDTIKNTAKNLAVGLLDKIKCKMTGC | Anti-Gram negative, Antifungal | 28 | 3 | 46% |
| 69 | Ranatuerin-2PLd (frog, amphibians, animals; XXU; 1S=S, UCSS1a) | pickerel frog, *Rana palustris*, North America | GIMDSVKNVAKNIAGQLLDKLKCKITGC | Anti-Gram negative | 28 | 3 | 46% |
| 70 | Ranatuerin-2PLe (frog, amphibians, animals; XXU; 1S=S, UCSS1a) | pickerel frog, *Rana palustris*, North America | GIMDSVKNAAKNLAGQLLDTIKCKITAC | Anti-Gram negative | 28 | 2 | 50% |
| 71 | Ranatuerin-2PLf (frog, amphibians, animals; XXU; 1S=S, UCSS1a) | pickerel frog, *Rana palustris*, North America | GIMDTVKNAAKDLAGQLDKLKCRITGC | Anti-Gram negative, Antifungal | 27 | 2 | 44% |
| 72 | Esculentin-2P (frog, amphibians, animals; ZZHa_t; XXU; 1S=S, UCSS1a) | North American frog, *Rana pipiens* | GFSSIFRGVAKFASKGLGKDLARLGVNLVACKISKQC | Anti-Gram negative, Antiviral, Anti-HIV, | 37 | 6 | 48% |
| 73 | A3-APO (A3apo; ARV-1501; Pro-rich, synthetic; Dimer derivative: Chex1; Chex-Arg20; ARV-1502) | engineered | RPDKPRPYLPRPRPPRPVR | Anti-Gram negative | 19 | 6 | 10% |
| 74 | Chicken AvBD4 (AvBD-4, chicken avian beta defensin 4; ; 3S=S; OLD gallinacin 7, Gal 7, Gal7, Gal-7, avian beta-defensin, UCSS1a; birds, animals; JJsn) | *Gallus gallus domestic* | RYHMQCGYRGTFCTPGKCPYGNAYLGLCRPKYSCCRWL | Anti-Gram negative | 38 | 6 | 34% |
| 75 | Chicken AvBD5 (AvBD-5, chicken avian beta defensin 5; 3S=S; OLD gallinacin 9, Gal 9, Gal9, Gal-9; UCSS1a; birds, animals) | *Gallus gallus domestic* | GLPQDCERRGGFCSHKSCPPGIGRIGLCSKEDFCCRSRWYS | Anti-Gram negative, Antiviral | 41 | 3 | 31% |
| 76 | Chicken LEAP-2 (cLEAP-2, GgLEAP2; CjLEAP-2; birds, animals; BBMm) | chicken, *Gallus gallus* or Japanese quail*, Coturnix japonica* | MTPFWRGVSLRPVGASCRDNSECITMLCRKNRCFLRTASE | Anti-Gram negative | 40 | 4 | 42% |
| 77 | Caenopore-5 (SPP-5; saposin-like protein family, roundworms, nematode, invertebrate, animals; 3S=S; UCSS1a) | ntestine, *Caenorhabditis elegans* | RSALSCQMCELVVKKYEGSADKDANVIKKDFDAECKKLFHTIPFGTRECDHYVNSKVDPIIHELEGGTAPKDVCTKLNECP | Anti-Gram negative | 81 | -2 | 37% |
| 78 | Hepcidin TH2-3 (4S=S, UCSS1a; fish, animals) | tilapia, *Oreochromis mossambicus* | QSHLSLCRWCCNCCRSNKGC | Anti-Gram negative, Anticancer | 20 | 3 | 45% |
| 79 | Caerulein precursor-related fragment Ea (CPRF-Ea, UCLL1; frog, amphibians, animals) | Edible frog, the common water frog, green frog, *Rana esculenta*, Europe | GLGSILGKILNVAGKVGKTIGKVADAVGNKE | Anti-Gram negative | 31 | 3 | 41% |
| 80 | Caerulein precursor-related fragment Eb (CPRF-Eb, UCLL1; frog, amphibians, animals) | Edible frog, the common water frog, green frog, *Rana esculenta*, Europe | GLGSFLKNAIKIAGKVGSTIGKVADAIGNKE | Anti-Gram negative | 31 | 3 | 41% |
| 81 | Caerulein precursor-related fragment Ec (CPRF-Ec, UCLL1; frog, amphibians, animals) | Edible frog, the common water frog, green frog, *Rana esculenta*, Europe | GLGSFFKNAIKIAGKVGSTIGKVADAIGNKE | Anti-Gram negative | 31 | 3 | 41% |
| 82 | Ranatuerin-2G (UCLL1a; XXU; 1S=S, frog, amphibians, animals) | *Rana grylio*, North America | GLLLDTLKGAAKDIAGIALEKLKCKITGCKP | Anti-Gram negative | 31 | 3 | 48% |
| 83 | Brevinin-2BYb (frog, amphibians, animals; XXU; 1S=S, UCSS1a) | *Rana boylii*, North America | GIMDSVKGLAKNLAGKLLDSLKCKITGC | Anti-Gram negative | 28 | 3 | 46% |
| 84 | Pilosulin 5 (Myr b III)(antss, insects, arthropods, invertebrates, animals) | *Myrmecia pilosula* (venom, Australia) | DVKGMKKAIKGILDCVIEKGYDKLAAKLKKVIQQLWE | Anti-Gram negative, Antifungal | 37 | 4 | 45% |
| 85 | Dermaseptin-L1 (Dermaseptin L1, DRS-L1; UCLL1; frog, amphibians, animals) | *Hylomantis lemur* (Hylidae: Phyllomedusinae) | GLWSKIKEAAKAAGKAALNAVTGLVNQGDQPS | Anti-Gram negative, Antifungal, Anticancer | 32 | 2 | 43% |
| 86 | kalata B13 (kB13; cyclotides; plants; XXC; 3S=S, UCBB1b; DXWZ) | African plant *Oldenlandia affinis DC* (Rubiaceae) | GLPVCGETCFGGTCNTPGCACDPWPVCTRD | Anti-Gram negative | 30 | -2 | 40% |
| 87 | Magainin-AM2 (UCLL1a; frog, amphibians, animals; Magainin-AM1: MIC>200 uM inactive) | the octoploid frog *Xenopus amieti* (Pipidae) | GVSKILHSAGKFGKAFLGEINKS | Anti-Gram negative | 23 | 4 | 39% |
| 88 | Tricyclon A (cyclotides; plants; XXC, 3S=S, UCBB1b; ZZHp) | Australian flowers, V. tricolor | GGTIFDCGESCFLGTCYTKGCSCGEWKLCYGTN | Anti-Gram negative, Antiviral, Antifungal, Anti-HIV | 33 | -1 | 36% |
| 89 | Microcin E492 (MccE492, class 2b microcins, the siderophore-mocrocin family; bacteriocin, Gram-negative bacteria, prokaryotes; BBPP; BBII, XXB; XXG, BBBh2o; Variants: u-Mcc492) | *Klebsiella pneumoniae RYC492* | GETDPNTQLLNDLGNNMAWGAALGAPGGLGSAALGAAGGALQTVGQGLIDHGPVNVFIPVLIGPSWNGSGSGYNSATSSSGSGS | Anti-Gram negative, Anticancer | 84 | -4 | 35% |
| 90 | B. pascuorum apidaecin (Pro-rich; insects, arthropods, invertebrates, animals) | *Bombus pascuorum* (Hymenoptera, Apoidea) | GNRPVYIPPPRPPHPRL | Anti-Gram negative | 17 | 4 | 17% |
| 91 | Microcin B17 (MccB17, Gly-rich, class 1 microcins, bacteriocins, Gram-negative bacteria, prokaryotes; BBren) | *Escherichia coli* | VGIGGGGGGGGGGSCGGQGGGCGGCSNGCSGGNGGSGGSGSHI | Anti-Gram negative | 43 | 0 | 16% |
| 92 | Microcin V (MccV, (old name) Colicin V, ColV; class 2a microcins, bacteriocins, Gram-negative bacteria, prokaryotes, BBMm) | *Escherichia coli* | ASGRDIAMAIGTLSGQFVAGGIGAAAGGVAGGAIYDYASTHKPNPAMSPSGLGGTIKQKPEGIPSEAWNYAAGRLCNWSPNNLSDVCL | Anti-Gram negative | 88 | 0 | 39% |
| 93 | Microcin L (MccL, class 2a microcins, bacteriocins, Gram-negative bacteria, prokaryotes, BBMm) | *Escherichia coli* LR05 | GDVNWVDVGKTVATNGAGVIGGAFGAGLCGPVCAGAFAVGSSAAVAALYDAAGNSNSAKQKPEGLPPEAWNYAEGRMCNWSPNNLSDVCL | Anti-Gram negative | 90 | -3 | 45% |
| 94 | Microcin M (MccM, class 2b microcins,the siderophore-microcin family, bacteriocins, Gram-negative bacteria, prokaryotes; BBPP, BBII, XXB) | *Escherichia coli* MC4100, *Escherichia coli Nissle* 1917, *Escherichia coli* CA46, *Escherichia coli* CA58, *Escherichia coli* MC4100(pMM75) | DGNDGQAELIAIGSLAGTFISPGFGSIAGAYIGDKVHSWATTATVSPSMSPSGIGLSSQFGSGRGTSSASSSAGSGS | Anti-Gram negative | 77 | -2 | 32% |
| 95 | Microcin H47 (MccH47, class 2b microcins, bacteriocins, the siderophore-microcin family, Gram-negative bacteria, prokaryotes, BBPP; BBII; XXB) | *Escherichia coli* H47, *Escherichia coli Nissle* 1917, *Escherichia* coli CA46, *Escherichia coli* CA58, *Escherichia coli* MC4100(pMM1.15) | GGAPATSANAAGAAAIVGALAGIPGGPLGVVVGAVSAGLTTGIGSTVGSGSASSSAGGGS | Anti-Gram negative | 60 | 0 | 43% |
| 96 | Microcin I47 (MccI47, class 2b microcins, bacteriocins, Gram-negative bacteria, prokaryotes; XXB) | *Escherichia coli* H47 | MNLNGLPASTNVIDLRGKDMGTYIDANGACWAPDTPSIIMYPGGSGPSYSMSSSTSSANSGS | Anti-Gram negative | 62 | -2 | 30% |
| 97 | Ranatuerin-2PRa (Ranatuerin 2PRa, frog, amphibians, animals; XXU; 1S=S, UCSS1a) | the Hokkaido frog, *Rana pirica*, Asia | GLMDVFKGAAKNLLASALDKIRCKVTKC | Anti-Gram negative, Antifungal | 28 | 4 | 53% |
| 98 | Temporin-1TGb (XXA; UCLL1c; frog, amphibians, animals) | Tago's brown frog, *Rana tagoi,* Japan, Asia | AVDLAKIANKVLSSLF | Anti-Gram negative | 16 | 2 | 62% |
| 99 | Palustrin-3AR (frog, amphibians, animals; ZZHa_t; XXU; 1S=S, UCSS1a) | the crawfish frog, *Rana areolata*, North America | GIFPKIIGKGIVNGIKSLAKGVGMKVFKAGLNNIGNTGCNNRDEC | Anti-Gram negative, Antiviral, Anti-HIV | 45 | 5 | 40% |
| 100 | Ranatuerin-2ARa (frog, amphibians, animals; XXU; 1S=S, UCSS1a) | the crawfish frog, *Rana areolata*, North America | GLMDTVKNAAKNLAGQLLDTIKCKMTGC | Anti-Gram negative | 28 | 2 | 46% |
| 101 | Microcin 24 (Mcc24, microcin N; OLD name: colicin 24, class 2a microcins, bacteriocin; Revisited; Gram-negative bacteria, prokaryotes, BBMm) | *E. coli* 2424 | GDPLADPNSQIVRQIMSNAAWGAAFGARGGLGGMAVGAAGGVTQTVLQGAAAHMPVNVPIPKVPMGPSWNGSKG | Anti-Gram negative | 74 | 2 | 43% |
| 102 | T07C4.4 (SPP-1; saposin-like protein, SAPLIP, 3S=S; UCSS1a; roundworm, nematoda, animals; BBMm) | *Caenorhabditis elegans* | NPANPLNLKKHHGVFCDVCKALVEGGEKVGDDDLDAWLDVNIGTLCWTMLLPLHHECEEELKKVKKELKKDIENKDSPDKACKDVDLC | Anti-Gram negative | 88 | -6 | 39% |
| 103 | Temporin-LTb (Chinese broad-folded frog, amphibians, animals, UCLL1c; XXA, BBMm) | *Hylarana latouchii* (Anura:Ranidae), China | FIITGLVRGLTKLF | Anti-Gram negative | 14 | 3 | 57% |
| 104 | AalDefD (Aedes albopictus defensin D, defensins; UCSS1a; 3S=S, insects, arthropods, invertebrate, animals, ZZP) | mosquito*, Aedes albopictus* | ATCDLLSGFGVGDSACAAHCIARGNRGGYCNSKKVCVCPI | Anti-Gram negative, Antiparasitic | 40 | 2 | 47% |
| 105 | Smd1 (S. calcitrans defensin 1; defensins; UCSS1a; insects, arthropods, invertebrate, animals; 3S=S) | mRNA in anterior midgut tissues, *Stomoxys calcitrans* | AAKPMGITCDLLSLWKVGHAACAAHCLVLGDVGGYCTKEGLCVCKE | Anti-Gram negative | 46 | 0 | 54% |
| 106 | Smd2 (S. calcitrans defensin 2; defensins; UCSS1a; 3S=S, insects, arthropods, invertebrate, animals) | mRNA in anterior midgut tissues, *Stomoxys calcitrans* | ATCDLLSMWNVNHSACAAHCLLLGKSGGRCNDDAVCVCRK | Anti-Gram negative | 40 | 1 | 52% |
| 107 | Pelovaterin (defensin-like AMP; glycine-rich, UCSS1a; 3S=S, turtles, reptiles, animals; BBBh2o) | Chinese soft-shelled turtle, Pelodiscus sinensis | DDTPSSRCGSGGWGPCLPIVDLLCIVHVTVGCSGGFGCCRIG | Anti-Gram negative | 42 | -1 | 42% |
| 108 | TEWP (OvoDB, Ovodefensin, turtle egg-white protein, the defensin-like peptide from marine turtle, reptilian defensin, UCSS1a; 3S=S, reptiles, animals; XXQ) | Red sea turtle Caretta caretta | QKKCPGRCTLKCGKHERPTLPYNCGKYICCVPVKVK | Anti-Gram negative, Antiviral | 36 | 8 | 33% |
| 109 | Casecidin 17 (cattle, ruminant, animals) | bovine colostrum | YQEPVLGPVRGPFPIIV | Anti-Gram negative | 17 | 0 | 41% |
| 110 | Casecidin 15 (cattle, ruminant, animals | bovine colostrum | YQEPVLGPVRGPFPI | Anti-Gram negative | 15 | 0 | 33% |
| 111 | Isracidin (cattle, ruminant, animals; VIHNN; Derivatives: Caseicin A; Caseicin B) | bovine colostrum | RPKHPIKHQGLPQEVLNENLLRF | Anti-Gram negative | 23 | 2 | 30% |
| 112 | Leptoglycin (UCLL1; frog, amphibians, animals; Gly-rich, Leu-rich) | the South American frog Leptodactylus pentadactylus | GLLGGLLGPLLGGGGGGGGGLL | Anti-Gram negative | 22 | 0 | 36% |
| 113 | Laticeptin (XXA; UCLL1c; frog, amphibians, animals) | South America, Leptodatylus laticeps | GVVDILKGAAKDLAGHLATKVMNKL | Anti-Gram negative | 25 | 3 | 52% |
| 114 | Brevinin-2PTc (frog, amphibians, animals; XXU; 1S=S, UCSS1a) | Hylarana picturata, Asia | GLLDSFKNAMIGIAKSAGKTALNKIACKIDKTC | Anti-Gram negative | 33 | 4 | 48% |
| 115 | Japonicin-1CDYa (frog, amphibians, animals; XXU; 1S=S, UCSS1a) | Rana chensinensis, China, Asia | FFPLALLCKVFKKC | Anti-Gram negative | 14 | 3 | 71% |
| 116 | Alyteserin-1a (XXA; UCLL1c; toad, amphibians, animals) | the European midwife toad, Alytes obstetricans | GLKDIFKAGLGSLVKGIAAHVAN | Anti-Gram negative | 23 | 3 | 52% |
| 117 | Alyteserin-1b (XXA; UCLL1c; toad, amphibians, animals) | the European midwife toad, Alytes obstetricans | GLKEIFKAGLGSLVKGIAAHVAN | Anti-Gram negative | 23 | 3 | 52% |
| 118 | Alyteserin-1c (XXA; UCLL1c; toad, amphibians, animals) | the European midwife toad, Alytes obstetricans | GLKEIFKAGLGSLVKGIAAHVAS | Anti-Gram negative | 23 | 3 | 52% |
| 119 | Calcitonin gene-related peptide (CGRP, XXA; neuropeptide; human; primates, mammals, animals) | Homo sapiens | ACDTATCVTHRLAGLLSRSGGVVKNNFVPTNVGSKAF | Anti-Gram negative, Antifungal | 37 | 4 | 43% |
| 120 | DEFB24 (beta-defensin; UCSS1a; 3S=S, rats, rodents, mammals, animals) | male reproductive tract, epididymis, Rattus norvegicus | GKNPTLQCMGNRGFCRPSCKKGEQAYFYCRTYQICCLQSHVRISLTGVEDNTNWSYEKHWPRIP | Anti-Gram negative | 64 | 5 | 31% |
| 121 | DEFB30 (beta-defensin, UCSS1a; 3S=S, rats, rodents, mammals, animals) | male reproductive tract, epididymis, Rattus norvegicus | GVNMYIRQIYDTCWKLKGHCRNVCGKKEIFHIFCGTQFLCCIERKEMPVLFVK | Anti-Gram negative | 53 | 7 | 47% |
| 122 | Ls-Stylicin1 (pacific blue shrimp, crustaceans, arthropods, invertebrate, animals; BBL; BBBh2o; ZZagg) | Litopenaeus stylirostris | SSFSPPRGPPGWGPPCVQQPCPKCPYDDYKCPTCDKFPECEECPHISIGCECGYFSCECPKPVCEPCESPIAELIKKGGYKG | Anti-Gram negative, Antifungal | 82 | -3 | 30% |
| 123 | Nigrocin-2SCa (frog, amphibians, animals; XXU; 1S=S, UCSS1a) | Odorrana schmackeri, Asia | GILSGILGAGKSLVCGLSGLC | Anti-Gram negative | 21 | 1 | 52% |
| 124 | Nigrocin-2SCc (frog, amphibians, animals; XXU; 1S=S, UCSS1a) | Odorrana schmackeri, Asia | GILSNVLGMGKKIVCGLSGLC | Anti-Gram negative | 21 | 2 | 52% |
| 125 | Nigrocin-2VB (frog, amphibians, animals; XXU; 1S=S, UCSS1a) | Odorrana versabilis, Asia | SILSGNFGVGKKIVCGLSGLC | Anti-Gram negative | 21 | 2 | 47% |
| 126 | Dermaseptin DA4 (DRS-DA4, UCLL1; frog, amphibians, animals) | Pachymedusa dacnicolor, Mexican, North America | GMWSKIKNAGKAAKAAAKAAGKAALGAVSEAM | Anti-Gram negative, Chemotactic | 32 | 5 | 56% |
| 127 | Drosophila diptericin (glycopeptide; insects, arthropods, invertebrates, animals; XXA; XXG) | fruit fly, Drosophila melanogaster | DDMTMKPTPPPQYPLNLQGGGGGGSGDGFGFAVQGHQKVWTSDNGRHEIGLNGGYGQHLGGPYGNSEPSWKVGSTYTYRFPNF | Anti-Gram negative | 83 | -1 | 20% |
| 128 | Drosophila MPAC (Maturated Pro-domain of Attain C, Pro-rich; glycopeptide; insects, arthropods, invertebrates, animals; XXQ; XXG) | fruit fly, Drosophila melanogaster | QRPYTQPLIYYPPPPTPPRIYRA | Anti-Gram negative | 23 | 3 | 17% |
| 129 | BIN1b (also named as sperm associated antigen 11E, SPAG11E, beta-defensin like; UCSS1a; rats, rodents, mammals, animals; 3S=S) | rat epididymis, *Rattus norvegicus* | GIRNTVCFMQRGHCRLFMCRSGERKGDICSDPWNRCCVSSSIKNR | Anti-Gram negative | 45 | 6 | 37% |
| 130 | Gallin (OvoDA, chicken ovodefensin, UCSS1a; 3S=S, birds, animals) | Gallus gallus | LVLKYCPKIGYCSNTCSKTQIWATSHGCKMYCCLPASWKWK | Anti-Gram negative | 41 | 6 | 43% |
| 131 | HbbetaP-1 (fish, animals; ZZP) | catfish, Ictalurus punctatus, Rafinesque | AANFGPSVFTPEVHETWQKFLNVVVAALGKQYH | Anti-Gram negative, Antiparasitic | 33 | 0 | 45% |
| 132 | Oncopeltus antibacterial peptide 4 (Pro-rich, insects, arthropods, invertebrates, animals; Derivatives: oncocin; Onc112; Onc72; XXA; BBP; BBribo) | the milkweed bug, Oncopeltus fasciatus | VDKPPYLPRPPPPRRIYNNR | Anti-Gram negative | 20 | 5 | 15% |
| 133 | Sushi peptide 1 (sushi1, S1, synthetic; BBMm, BBL; derivatives:MIRIAM) | Protein-derived peptide | GFKLKGMARISCLPNGQWSNFPPKCIRECAMVSS | Anti-Gram negative | 34 | 4 | 44% |
| 134 | Sushi peptide 3 (S3, synthetic; a homodimer; 1S=S; BBL) | Protein-derived peptide | HAEHKVKIGVEQKYGQFPQGTEVTYTCSGNYFLM | Anti-Gram negative | 34 | 0 | 29% |
| 135 | gcLEAP-2 (grass carp liver-expressed antimicrobial peptide-2; fish, animals) | Chinese grass carp, Ctenopharyngodon idella | MTPLWRIMGTKPHGAYCQNHYECSTGICRKGHCSYSQPINS | Anti-Gram negative | 41 | 3 | 29% |
| 136 | EC-hepcidin2 (E. coioides hepcidin 2; UCSS1a; ?S=S, fish, animals) | Orange-spotted liver, grouper, Epinephelus coioides | MKTFSVAVAVAVVLAFICTQESSALPVTGIEELVEPVSSDNNDNHQGLPVELRERLVNIRKKRAPTDCIPYCYPTGDGFHCGVTCRF | Anti-Gram negative, Antiviral | 87 | -2 | 42% |
| 137 | Capistruin ( lactam; a class 2 lasso peptide; class 1 microcin, bacteriocins; Gram-negative bacteria, prokaryotes; XXJ; UCSB1a; BBpol) | Burkholderia thailandensis E264 | GTPGFQTPDARVISRFGFN | Anti-Gram negative | 19 | 1 | 31% |
| 138 | Rhamp (R. haemaphysaloides AMP; UCSS1a; hard tick, Nuttalliellidae, arachnids, Chelicerata, arthropods, invertebrates, animals; 5S=S) | salivary glands, Rhipicephalus haemaphysaloides, Asia | ERILDLRKTKKSCKNGEVLGCVSGHGPPGCSENECGMGPRPKACFFDCHYGCWCTGKLYRRKRDRKCVPKHECLL | Anti-Gram negative | 75 | 8 | 33% |
| 139 | limnonectin-1Fa (frog, amphibians, animals; XXU; 1S=S, UCSS1a) | the Fujian large-headed frog, Limnonectes fujianensis, China, Asia | SFPFFPPGICKRLKRC | Anti-Gram negative | 16 | 4 | 43% |
| 140 | limnonectin-1Fb (frog, amphibians, animals; XXU; 1S=S, UCSS1a) | the Fujian large-headed frog, Limnonectes fujianensis, China, Asia | SFHVFPPWMCKSLKKC | Anti-Gram negative | 16 | 3 | 50% |
| 141 | Vejovine (scorpions, arachnids, Chelicerata, arthropods, invertebrates, animals; UCLL1a) | the scorpion venom, Vaejovis mexicanus | GIWSSIKNLASKAWNSDIGQSLRNKAAGAINKFVADKIGVTPSQAAS | Anti-Gram negative, Hemolytic | 47 | 4 | 42% |
| 142 | aCATH (Gly-rich; ayu cathelicidin; fish, animals) | highly expressed in spleen, liver, and head kidney, ayu, Plecoglossus altivelis | RMRRSKSGKGSGGSKGSGSKGSKGSKGSGSKGSGSKGGSRPGGGSSIAGGGSKGKGGTQTA | Anti-Gram negative | 61 | 14 | 6% |
| 143 | Andersonin-C1 (XXU; 1S=S, UCSS1a; frog, amphibians, animals) | Odorrana andersonii, China, Asia | TSRCIFYRRKKCS | Anti-Gram negative, Antifungal | 13 | 5 | 30% |
| 144 | Ranatuerin-2SKa (XXU; 1S=S, UCSS1a; frog, amphibians, animals) | skin, the stream brown frog, Rana sakuraii, Japan, Asia | GLLDAIKDTAQNLFANVLDKIKCKFTKC | Anti-Gram negative | 28 | 2 | 50% |
| 145 | Brevinin-2SKb (frog, amphibians, animals; XXU; 1S=S, UCSS1a) | skin, the stream brown frog, Rana sakuraii, Japan, Asia | GLFNVFKKVGKNVLKNVAGSLMDNLKCKVSGEC | Anti-Gram negative | 33 | 4 | 45% |
| 146 | ChaC7 (chassatide C7, uncyclotides; UCSS1a; 3S=S, plants) | Chassalia chartacea (or Chassalia curviflora) | IPCGESCVWIPCITAIAGCSCKNKVCYT | Anti-Gram negative, Hemolytic, Anticancer | 28 | 1 | 53% |
| 147 | ChaC8 (chassatide C8, uncyclotides; UCSS1a; 3S=S, plants) | Chassalia chartacea (or Chassalia curviflora) | AIPCGESCVWIPCISTVIGCSCSNKVCYR | Anti-Gram negative, Hemolytic, Anticancer | 29 | 1 | 51% |
| 148 | ChaC11 (chassatide C11, uncyclotides; UCSS1a; 3S=S, plants. Variants: chaC11A, XXO) | Chassalia chartacea (or Chassalia curviflora) | IPCGESCVWIPCISGMFGCSCKDKVCYS | Anti-Gram negative, Hemolytic, Anticancer | 28 | 0 | 50% |
| 149 | PGLa-SE1 (PGLa-SP1; frog, amphibians, animals; XXA; UCLL1c) | Cameroon Clawed Frog, the tetraploid frog Silurana epitropicalis SE1, and Silurana paratropicalis, Africa | GMATKAGTALGKVAKAVIGAAL | Anti-Gram negative | 22 | 4 | 59% |
| 150 | XPF-SE3 (frog, amphibians, animals; XXA; UCLL1c) | Cameroon Clawed Frog, the tetraploid frog Silurana epitropicalis SE1, Africa | GFWTTAAEGLKKFAKAGLASILNPK | Anti-Gram negative | 25 | 4 | 48% |
| 151 | Microcin S (MccS, class 2a microcin, bacteriocin, Gram-negative bacteria, prokaryotes) | Escherichia coli G3/10 | GNANSNYEGGGSRSRNTGARNSLGRNAPTHIYSDPSTVKCANAVFSGMVGGAIKGGPVGMTRGTIGGAVIGQCLSGGGNGNGGGNRAGSSNCSGSNVGGTCSR | Anti-Gram negative | 103 | 7 | 26% |
| 152 | Microcin PDI (MccPDI, class 2a microcin, bacteriocin, Gram-negative bacteria, prokaryotes) | Escherichia coli 25 | NANSNFEGGPRNDRSSGARNSLGRNAPTHIYSDPSTVKCANAVFSGMIGGAIKGGPIGMARGTIGGAVVGQCLSDHGSGNGSGNRGSSSSCSGNNVGGTCNR | Anti-Gram negative | 102 | 5 | 27% |
| 153 | LTP110 (lipid transfer protein, plants; 4S=S; UCSS1a) | rice leaves, Oryza sativa Indica Group | VSCGDVTSSIAPCLSYVMGRESSPSSSCCSGVRTLNGKASSSADRRTACSCLKNMASSFRNLNMGNAASIPSKCGVSVAFPISTSVDCSKIN | Anti-Gram negative, Antifungal | 92 | 5 | 39% |
| 154 | Astexin-1 (class 2 lasso, bacteriocin, Gram-negative bacteria, prokaryotes) | Asticcacaulis excentricus CB 48 | GLSQGVEPDIGQTYFEESRINQD | Anti-Gram negative | 23 | -4 | 21% |
| 155 | Psoriasin (S100A7; metal-binding protein, BBII; humans; primates, mammals, animals) | keratinocytes, Skin, tongue Homo sapiens | MSNTQAERSIIGMIDMFHKYTRRDDKIDKPSLLTMMKENFPNFLSACDKKGTNYLADVFEKKDKNEDKKIDFSEFLSLLGDIATDYHKQSHGAAPCSGGSQ | Anti-Gram negative, Chemotactic | 101 | -1 | 32% |
| 156 | CCL8 (MCP-2, chemokine, CC family; kinocidins; UCSS1a; 2S=S, human, primates, mammals, animals) | Homo sapiens | PDSVSIPITCCFNVINRKIPIQRLESYTRITNIQCPKEAVIFKTKRGKEVCADPKERWVRDSMKHLDQIFQNLKP | Anti-Gram negative, Chemotactic | 75 | 6 | 37% |
| 157 | CCL13 (MCP-4, chemokine, CC family; kinocidins; UCSS1a; 2S=S, human, primates, mammals, animals) | Homo sapiens | QPDALNVPSTCCFTFSSKKISLQRLKSYVITTSRCPQKAVIFRTKLGKEICADPKEKWVQNYMKHLGRKAHTLKT | Anti-Gram negative, Chemotactic | 75 | 11 | 36% |
| 158 | CCL19 (MIP-3beta, chemokine, CC family; kinocidins; human, primates, mammals, animals) | Homo sapiens | GTNDAEDCCLSVTQKPIPGYIVRNFHYLLIKDGCRVPAVVFTTLRGRQLCAPPDQPWVERIIQRLQRTSAKMKRRSS | Anti-Gram negative, Chemotactic | 77 | 7 | 37% |
| 159 | Ctenidin-1 (Gly-rich; spiders, arachnids, Chelicerata, arthropods, invertebrates, animals; UCLL1a) | Cupiennius salei | DRGYGGGRRGGGYGGGGYGGGGYGGGGGGYGGGVGGGRGGGGGLGGGRGGGGGVIDGKDDVGLGGGGYGGGLGGGQGGGGGLGGGQGGGGGLGGGRGGGGYGGGGGGYGGGKYGGGKYG | Anti-Gram negative | 119 | 5 | 7% |
| 160 | Ctenidin-2 (Gly-rich; spiders, arachnids, Chelicerata, arthropods, invertebrates, animals; UCLL1a) | Cupiennius salei | DRGYGGGRRGGGYGGGGYGGGGYGGGGGGYGGGVGGGRGGGGGLGGGRGGGGGVIDGKDDVGLGGGGYGGGLGGGQGGGGGLGGGRGGGGYGGGGGGYGGGKYGGGKYG | Anti-Gram negative | 109 | 5 | 7% |
| 161 | Ctenidin-3 (Gly-rich; spiders, arachnids, Chelicerata, arthropods, invertebrates, animals; UCLL1a) | Cupiennius salei | DRGYGGGRRGGGYGGGGYGGGGGGYGGGGGGYGGGVGGGRGGGGGLGGGRGGGGGLVDGKDDVGLGGGGYGGGLGGGQGGGGGLGGGQGGGGGLGGGRGGGGYGGGGGGYGGGKYGGGKY | Anti-Gram negative | 120 | 5 | 7% |
| 162 | Warnericin RK (bacteriocin; hemolysins; Gram-positive bacteria, prokaryotes; BBMm) | Staphylococcus warneri RK | MQFITDLIKKAVDFFKGLFGNK | Anti-Gram negative, Hemolytic | 22 | 2 | 50% |
| 163 | delta-lysin I (bacteriocins; hemolysins; Gram-positive bacteria, prokaryotes) | Staphylococcus warneri RK | MAADIISTIGDLVKLIINTVKKFQK | Anti-Gram negative, Hemolytic | 25 | 2 | 52% |
| 164 | UyCT2 (scorpions, arachnids, Chelicerata, arthropods, invertebrates, animals; XXA, UCLL1c | venom, Urodacus yaschenkoi, Australia | FWGKLWEGVKNAI | Anti-Gram negative | 13 | 2 | 53% |
| 165 | Haxy_Col1 (Coleoptericins; insects, arthropods, invertebrates, animals. More AMPs?) | ladybird beetle, Harmonia axyridis | SLQPGAPNFPIPGQEKQEGWKFDPSLTRGEDGNTLGSINIHHTGPNHEVGANWDKVIRGPGKAKPTYSIHGSWRW | Anti-Gram negative, Antifungal | 75 | 1 | 25% |
| 166 | Haxy_Col6 (Coleoptericins; insects, arthropods, invertebrates, animals) | ladybird beetle, Harmonia axyridis | SLQPGAPKLPYAWSRKQEGWKFDPSLTRGEDGNTLGSINIHHTGRNHEVGANWNKVIRGPGKAKPTYSIHGSWRW | Anti-Gram negative, Antifungal | 75 | 6 | 26% |
| 167 | Pis2 (piscidin; His-rich; fish, animals, UCLL1: Variants: GAD-2) | Atlantic cod, Gadus morhua L. | FLHHIVGLIHHGLSLFGDRAD | Anti-Gram negative, Anticancer | 21 | 3 | 47% |
| 168 | ALF-L (LALF, Limulus anti-LPS factor; LpALF; UCSS1a; 1S=S, Horseshoe Crab, arachnids, Chelicerata, arthropods, invertebrates, animals; XXI; BBS) | American Limulus polyphemus | DGIWTQLIFTLVKNLATLWQSGDFQFLDHECHYRIKPTFRRLKWKYKGKFWCPSWTSITGRATKSSRSGAVEHSVRNFVGQAKSSGLITQRQAEQFISQYN | Anti-Gram negative | 101 | 9 | 35% |
| 169 | PtALF1 (Portunus trituberculatus anti-lipopolysaccharide factor isoform 1; Crustaceans, arthropods, invertebrates, animals; BBS) | swimming crab Portunus trituberculatus | YEALVTSILGKLTGLWHNDSVDFMGHICYFRRRPKIRRFKLYHEGKFWCPGWAPFEGRCKYCVVF | Anti-Gram negative, Antifungal | 65 | 6 | 43% |
| 170 | PtALF4 (Portunus trituberculatus anti-lipopolysaccharide factor isoform 4; Crustaceans, arthropods, invertebrates, animals; BBS) | eyestalk; swimming crab Portunus trituberculatus | GGWLDIVKAIVVPAARETIKTQEITLLDHYCTLSRSPYIKSLELHYRAEVTCPGWTIIRGRGSNHRNPTNSGKDALKDFMTQAVAAGLVTKEEAAPWLN | Anti-Gram negative, Antifungal | 99 | 2 | 39% |
| 171 | PtALF5 (Portunus trituberculatus anti-lipopolysaccharide factor isoform 5; Crustaceans, arthropods, invertebrates, animals; BBS) | swimming crab Portunus trituberculatus | YVDREINLFDHYCIISRSPHISRWELKWQATVTCPGWTPVKGKVRGYSNPLSAEREATRDFVQRIVQRGLVTRDEASEWL | Anti-Gram negative | 80 | 2 | 36% |
| 172 | TAP 20N (Tracheal antimicrobial peptide S20N variant, beta defensin; UCSS1a; 3S=S, cattle, ruminant, animals) | mucosal epithelial cells, Bos taurus | NPVSCVRNKGICVPIRCPGNMKQIGTCVGRAVKCCRKK | Anti-Gram negative | 38 | 9 | 42% |
| 173 | Panitide L2 (XXQ, UCSS1a; 3S=S, plants; Others Panitide L1, Panitide L3; Panitide L4, Panitide L5, Panitide L6, Panitide L7, Panitide L8, Panitide L9, inactive) | Panicum laxum | QLPICGETCVLGGCYTPNCRCQYPICVR | Anti-Gram negative, Hemolytic | 28 | 1 | 42% |
| 174 | Alarin (neuropeptide; human, primates, mammals, animals; BBMm) | brain, Homo sapiens | APAHRSSTFPKWVTKTERGRQPLRS | Anti-Gram negative | 25 | 5 | 24% |
| 175 | NRWC (name not yet found; bacteriocin, Gram-positive bacteria, prokaryotes) | Bacillus subtilis ATCC 6633 | NRWCFAGDD | Anti-Gram negative | 9 | -1 | 44% |
| 176 | Acanthoscurrin 1 (Gly-rich; spiders, arachnids, Chelicerata, arthropods, invertebrates, animals; UCLL1a) | Acanthoscurria gomesiana | DVYKGGGGGRYGGGRYGGGGGYGGGLGGGGLGGGGLGGGKGLGGGGLGGGGLGGGGLGGGGLGGGKGLGGGGLGGGGLGGGGLGGGGLGGGKGLGGGGLGGGGLGGGRGGGYGGGGGYGGGYGGGYGGGKYKG | Anti-Gram negative, Antifungal | 133 | 8 | 12% |
| 177 | Acanthoscurrin 2 (Gly-rich; spiders, arachnids, Chelicerata, arthropods, invertebrates, animals; UCLL1a) | Acanthoscurria gomesiana | DVYKGGGGGRYGGGRYGGGGGYGGGLGGGGLGGGGLGGGKGLGGGGLGGGGLGGGGLGGGGLGGGKGLGGGGLGGGGLGGGGLGGGGLGGGKGLGGGGLGGGGLGGGRGGYGGGGYGGGYGGGYGGGKYKG | Anti-Gram negative, Antifungal | 131 | 8 | 12% |
| 178 | Pelophylaxin-2GY (XXU; 1S=S, UCSS1a; frog, amphibians, animals) | Dark-spotted frogs, Pelophylax nigromaculatus, Guiyang, Guizhou; Kunming, Yunnan, China, Asia | GLLLDTVKGAAKNVAGILLNKLKCKMTGDC | Anti-Gram negative | 30 | 3 | 50% |
| 179 | HMGN2 (high mobility group nucleosomal binding domain 2, HMG-17, HCP-1, human, primates, mammals, animals) | human mononuclear leukocyte, Homo sapiens | PKRKAEGDAKGDKAKVKDEPQRRSARLSAKPAPPKPEPKPKKAPAKKGEKVPKGKKGKADAGKEGNNPAENGDAKTDQAQKAEGAGDAK | Anti-Gram negative, Antiviral, Antifungal | 89 | 12 | 21% |
| 180 | KDAMP 19-mer (KAMP-19; keratin-derived AMPs; Gly-rich; human; primates, mammals, animals. Several other shorter derivatives were isolated: KAMP-18N, KAMP-18C, KAMP-17, KAMP-14, and KAMP-13) | corneas, eyes, Homo sapiens | RAIGGGLSSVGGGSSTIKY | Anti-Gram negative | 19 | 2 | 26% |
| 181 | NRC-9 (fish, animals; XXA) | Winter flounder Y, Pleuronectes americanus | FFRLLFHGVHHGGGYLNAA | Anti-Gram negative | 19 | 2 | 47% |
| 182 | NRC-18 (fish, animals) | Witch flounder GC3.2, Glyptocephalus cynoglossus L. | GWKKWFTKGERLSQRHFA | Anti-Gram negative | 18 | 4 | 33% |
| 183 | NRC-20 (fish, animals) | Halibut Hb18, Hippoglossus hippoglossus L. | GFLGILFHGVHHGRKKALHMNSERRS | Anti-Gram negative | 26 | 4 | 34% |
| 184 | CPF-St7 (frog, amphibians, animals; XXA; UCLL1c) | the African clawed frog Silurana tropicalis | NLLGSLLKTGLKVGSNLL | Anti-Gram negative, Antifungal, Antiparasitic, Hemolytic, | 18 | 3 | 44% |
| 185 | Bacteriocin OR-7 (class 2a bacteriocins, Gram-positive bacteria; prokaryotes) | Lactobacillus salivarius strain NRRL B-30514 | KTYYGTNGVHCTKNSLWGKVRLKNMKYDQNTTYMGRLQDILLGWATGAFGKTFH | Anti-Gram negative | 54 | 6 | 31% |
| 186 | Cliotide T7 (CT7; Cter R; cyclotides; XXC; 3S=S, UCBB1b; Fabaceae, plants) | Clitoria ternatea | GIPCGESCVFIPCTVTALLGCSCKDKVCYKN | Anti-Gram negative, Anticancer | 31 | 1 | 48% |
| 187 | Cliotide T10 (CT10; Cter B; cyclotides; XXC; 3S=S, UCBB1b; Fabaceae, plants) | Clitoria ternatea | GVPCAESCVWIPCTVTALLGCSCKDKVCYLN | Anti-Gram negative, Anticancer | 31 | 0 | 54% |
| 188 | Cliotide T19 (CT19; cyclotides; XXC; 3S=S, UCBB1b; Fabaceae, plants) | Clitoria ternatea | GSVIKCGESCLLGKCYTPGCTCSRPICKKD | Anti-Gram negative, Anticancer | 30 | 3 | 36% |
| 189 | Microcin B (MccB, thiazole/oxazole modified microcins TOMM, bacteria) | Escherichia coli | VGIGGGGGGGGGGSCGGQGGGCGGCSNGCSGGNGGSGGSGSH | Anti-Gram negative | 42 | 0 | 14% |
| 190 | Mastoparan-VT5 (insects, arthropods, invertebrates, animals) | venom gland, the social wasp, Vespa tropica | VIVKAIATLSKKLL | Anti-Gram negative, Antifungal | 14 | 3 | 64% |
| 191 | MDAP-2 (insects, arthropods, invertebrates, animals) | house fly, Musca domestica | MKFFTLLAALMALFAICNNFSMVSASRDSRPVQPRVQPPPPPPKQKPSIYDTPIRRPGGQKTMYA | Anti-Gram negative | 65 | 7 | 38% |
| 192 | EsALF-2 (E. sinensis anti-lipopolysaccharide factor 2; Crustaceans, arthropods, invertebrates, animals;1S=S; BBL) | gill (high expression); haemocytes, hepatopancreas and muscle (moderate expression), Chinese mitten crab, Eriocheir sinensis | NIFDDIFGKVTETLVDFGTTDIAGNPCNYRLSPRLIKFELYFVGLVWCPGWTTIQGESLTRSRTRVVNKAVEDFAKKAVAAGIMTQEDADPLLNA | Anti-Gram negative, Antifungal | 95 | -2 | 43% |
| 193 | Magainin-F3 (frogs, amphibians, animals; UCLL1; More AMPs?) | skin secretions, Xenopus fraseri; and Xenopus andrei, Africa | GVSKILHSAGKFGKAFLGEIMKS | Anti-Gram negative | 23 | 3 | 43% |
| 194 | miiuy croaker LEAP-2 (liver-expressed antimicrobial peptide 2, 2S=S, fish, animals; UCSS1a) | widely expressed but mainly in Liver; Miichthys miiuy | MTPLWRIMNSKPFGAYCQNNYECSTGLCRAGHCSTSHRATSETVNY | Anti-Gram negative | 46 | 2 | 32% |
| 195 | Magainin-MW1 (frogs, amphibians, animals; UCLL1a) | Xenopus muelleri, West Africa | GIGKFLHSAGKFGKAFLGEVMKS | Anti-Gram negative, Antifungal | 23 | 3 | 43% |
| 196 | XPF-SP1 (frogs, amphibians, animals; UCLL1a | skin secrections, Silurana paratropicalis, Cameroon, Africa | GFWSSALEGLKKFAKGGLEALTNPK | Anti-Gram negative | 25 | 2 | 40% |
| 197 | XPF-SP2 (frogs, amphibians, animals; UCLL1a | skin secrections, Silurana paratropicalis, Cameroon, Africa | GLASTIGSLLGKFAKGGAQAFLQPK | Anti-Gram negative | 25 | 3 | 44% |
| 198 | LSer-Cec1 (cecropins; insects, arthropods, invertebrates, animals) | the blow fly, Lucilia sericata | GWLKKIGKKIERVGQHTRDATIQTIGVAQQAANVAATLKG | Anti-Gram negative | 40 | 5 | 40% |
| 199 | LSer-Cec2 (cecropins; insects, arthropods, invertebrates, animals) | the blow fly, Lucilia sericata | GWLRDFGKRIERVGQHTRDATIQAIGVAQQAANVAATVRG | Anti-Gram negative | 40 | 3 | 42% |
| 200 | LSer-Cec4 (cecropins; insects, arthropods, invertebrates, animals) | the blow fly, Lucilia sericata | GWLKKIGKKIERVGQHTRDASIQAIGIAQQAANVAATARG | Anti-Gram negative | 40 | 5 | 42% |
| 201 | LSer-Cec5 (cecropins; insects, arthropods, invertebrates, animals) | the blow fly, Lucilia sericata | GLVKKIGKKIERVGQHTRDASIQAIGIAQQAANVAATARG | Anti-Gram negative | 40 | 5 | 42% |
| 202 | LSer-Cec6 (cecropins; insects, arthropods, invertebrates, animals) | the blow fly, Lucilia sericata | GWLKKFGKKIERVGQHTRDATIQAIGVAQQAANVAATLKG | Anti-Gram negative | 40 | 5 | 42% |
| 203 | LSer-PCecL2 (putative cecropin-like peptides; insects, arthropods, invertebrates, animals) | the blow fly, Lucilia sericata | HHHHRFGKIGHELHKGVKKVEKVTHDVNKVTSGVKKVASSIEKAKNV | Anti-Gram negative | 47 | 7 | 29% |
| 204 | LSer-PCecL3 (putative cecropin-like peptides; insects, arthropods, invertebrates, animals) | the blow fly, Lucilia sericata | HHHFGRIGHELHKGVKKVEKVTSDVNKVTNGVKQVANGIAKAKTVIEAGSIAGAVAAAAA | Anti-Gram negative | 60 | 5 | 43% |
| 205 | LSer-PCecL5 (putative cecropin-like peptides; insects, arthropods, invertebrates, animals) | the blow fly, Lucilia sericata | HHHLFGHVGHEVERSLHKVGHKLEHACHEVHKTAKKVQK | Anti-Gram negative | 39 | 3 | 30% |
| 206 | Cm38 (scorpions, arachnids, Chelicerata, arthropods, invertebrates, animals; BWQ, UCLL1a?) | scorpion venom, Centruroides margaritatus | ARDGYIVDEKGCKFACFIN | Anti-Gram negative | 19 | 0 | 47% |
| 207 | PaLEAP-2 (P. altivelis liver-expressed antimicrobial peptide 2; fish, animals; 2S=S; UCSS1a) | liver, intestine, a teleost fish, Plecoglossus altivelis | MTPLWRVMGNKPFGAYCQDHVECSTGICKGGHCITSQPIKS | Anti-Gram negative | 41 | 2 | 46% |
| 208 | DEFB118 (human beta-defensin 118, formerly ESC42, beta-defensin, primates, mammals, animals; 3S=S; UCSS1a; BBMm) | epididymis, Homo sapiens | SGEKKCWNRSGHCRKQCKDGEAVKDTCKNLRACCIPSNEDHRRVPATSPTPLSDSTPGIIDDILTVRFTTDYFEVSSKKDMVEESEAGRGTETSLPNVHHSS | Anti-Gram negative | 102 | -1 | 27% |
| 209 | Opiscorpine 1 (scorpions, arachnids, Chelicerata, arthropods, invertebrates, animals; modular design; 3S=S, UCSS1a; Derivatives: Jan-f3; Janf1; more AMPs? Opiscorpine 2; Opiscorpine 3; Opiscorpine 4) | venom, Opistophthalmus carinatus , Africa | KWFNEKSIQNKIDEKIGKNFLGGMAKAVVHKLAKNEFMCVANVDMTKSCDTHCQKASGEKGYCHGTKCKCGVPLSY | Anti-Gram negative, Antifungal | 76 | 6 | 38% |
| 210 | Ocellatin-PT4 (frog, amphibians, animals, XXA, UCLL1c; poorly active (MIC 300 uM: ocellatin-PT1; ocellatin-PT5) | Skin Secretion, dorsal, Leptodactylus pustulatus | GVFDIIKGAGKQLIAHAMGKIAEKV | Anti-Gram negative | 25 | 3 | 52% |
| 211 | Ocellatin-PT7 (frog, amphibians, animals; UCLL1a) | Skin Secretion, dorsal, Leptodactylus pustulatus | GVFDIIKGAGKQLIAHAMGKIAEKVGLNKDGN | Anti-Gram negative | 32 | 2 | 43% |
| 212 | Ocellatin-PT8 (frog, amphibians, animals; UCLL1a) | Skin Secretion, ventral, Leptodactylus pustulatus | GVFDIIKGAGKQLIARAMGKIAEKVGLNKDGN | Anti-Gram negative | 32 | 3 | 43% |
| 213 | C-terminal Filaggrin-2 derived peptide (residues 2321-2391; human, primates, mammals, animals; UCLL1a; BBN) | skin, Homo sapiens | SSRASHFQSHSSERQRHGSSQVWKHGSYGPAEYDYGHTGYGPSGGSRKSISNSHLSWSTDSTANKQLSRH | Anti-Gram negative | 70 | 4 | 14% |
| 214 | CsCCK1 (chemokine, CC family; kinocidins; UCSS1a; 3S=S, fish, animals) | (highly expressed) spleen, liver, gill, gut, kidney, muscle, brain, heart (lowly expressed), Cynoglossus semilaevis | QPQMVIDCCLSVKNKTITKHIITDYHQQSAGQGCSIEATKKLCVPADEPWVHMVMSHVDKMKKPCHGKRRNKRCVKVKKMILVTRRN | Anti-Gram negative, Chemotactic | 87 | 12 | 37% |
| 215 | CsCCL17 (chemokine, CC family; kinocidins; UCSS1a; 2S=S, fish, animals) | spleen (high), liver, heart, gill and HK (moderate), and low in muscle, brain and intestine, half-smooth tongue sole, Cynoglossus semilaevis | QGGIASCCRRHSKTQINREHLTHYYEQHRPPCPIKAVVFYVIGGARICADPNKVWTKTSKAFLDGVHYQRQHTSSKVSF | Anti-Gram negative, Antiviral, Chemotactic | 79 | 8 | 32% |
| 216 | CsCCL21 (chemokine, CC family; kinocidins; UCSS1a; 2S=S, fish, animals) | induced in kidney, spleen, and liver, tongue sole, Cynoglossus semilaevis | QEFYGNCCLGHVKPMKIKGKRIESYRMQETDGDCHISAVVFLIKKKPSHVKQKTICANPQEAWVQELMAAVDSRNPKN | Anti-Gram negative, Antiviral, Chemotactic | 78 | 5 | 37% |
| 217 | BP100 (synthetic; XXA. Other derivatives: BP66, BP77, BP81, BP105, BP125, and BP126; BP143, BP145) | Library approach | KKLFKKILKYL | Anti-Gram negative | 11 | 6 | 45% |
| 218 | Cliotide 15 (cT15, cyclotides, plants; XXC, 3S=S, UCBB1a) | the Butterfly Pea, Clitoria ternatea | GLPICGETCFKTKCYTKGCSCSYPVCKRN | Anti-Gram negative | 29 | 4 | 34% |
| 219 | Cliotide 16 (cT16, cyclotides, plants; XXC, 3S=S, UCBB1a) | the Butterfly Pea, Clitoria ternatea | GSVIGCGETCLRGRCYTPGCTCDHGICKKN | Anti-Gram negative | 30 | 2 | 33% |
| 220 | Cliotide 20 (cT20, cyclotides, plants; XXC, 3S=S, UCBB1a) | the Butterfly Pea, Clitoria ternatea | GSAIRCGESCLLGKCYTPGCTCDRPICKKN | Anti-Gram negative | 30 | 3 | 36% |
| 221 | Defb22 (Rat beta-defensin 22; 2D6 glycoprotein; E-3 epididymal fluid protein; beta-defensin 126; mammals, animals; UCSS1a) | secreted fluid, epididymis; sperm tail; Rattus norvegicus | WYVRKCANKLGTCRKTCRKGEYQTDPATGKCSIGKLCCILDLKLAGQCGGADGNQAAAGTQAAGGTRAAGGTQGTGGTGATGAAATTAAP | Anti-Gram negative, Antifungal | 90 | 7 | 36% |
| 222 | SGCock_Contig04_0915 (insects, arthropods, invertebrates, animals, UCLL1) | the American Cockroach Periplaneta americana | GTFIKQQRKQKQQRHHTSGTRKRMAK | | 26 | 9 | 15% |
| 223 | ISGCock_Contig13_4610–1 (insects, arthropods, invertebrates, animals, UCLL1) | the American Cockroach Periplaneta americana | YPCKLNLKLGKVPFHF | Anti-Gram negative | 16 | 3 | 43% |
| 224 | ISGCock_Contig10_4736–2 (insects, arthropods, invertebrates, animals, UCLL1) | the American Cockroach Periplaneta americana | GTKRGKLCRISRLAL | Anti-Gram negative, Antifungal | 15 | 5 | 40% |
| 225 | ISGCock_Contig13_3006 (insects, arthropods, invertebrates, animals, UCLL1) | the American Cockroach Periplaneta americana | LRHKVYGYCVLGP | Anti-Gram negative, Antifungal | 13 | 2 | 38% |
| 226 | ISGCock_Contig12_4176 (insects, arthropods, invertebrates, animals, UCLL1) | the American Cockroach Periplaneta americana | VGRKHSILNCIPYLKKKKIMRL | Anti-Gram negative, Antifungal | 22 | 7 | 40% |
| 227 | Kunitzin-RE (amphibians, animals, UCSS1a) | European frog, Rana esculenta | AAKIILNPKFRCKAAFC | Anti-Gram negative, Enzyme inhibitor | 17 | 4 | 64% |
| 228 | Kunitzin-OS (amphibians, animals, UCSS1a) | Chinese frog, Odorrana schmackeri | AVNIPFKVHLRCKAAFC | Anti-Gram negative, Enzyme inhibitor | 17 | 3 | 64% |
| 229 | Gm0025x00667(75–100) (a fragment of the enzyme flavonoid 3-hydroxylase, UCLL1) | soybean, Glycine max | RWRFLRKISSVHMFSVKALDDFRQL | Anti-Gram negative | 25 | 4 | 48% |
| 230 | Gm0026x00785(77–103) (a fragment of lipoate-protein ligase B, UCLL1) | soybean, Glycine max | HKMDLHWYLRTLEEVVIRALQRFQFR | Anti-Gram negative | 26 | 2 | 46% |
| 231 | Thaulin-1 (frog, amphibians, animals; UCLL1a; Other sequences reported: Thaulin-2, Thaulin-3, Thaulin-4) | skin, a Patagonian frog, Pleurodema thaul, South America | NGNLLGGLLRPVLGVVKGLTGGLGKK | Anti-Gram negative | 26 | 4 | 38% |
| 232 | Cc-LTP2 (Coffea canephora lipid transfer protein 2, plants; BWQ) | seeds, Coffea canephora Pierre | ITCQQVTAELEPCVPYLTQGIP | Anti-Gram negative, Antifungal | 22 | -2 | 40% |
| 233 | Thionin-like peptide 1 (plants; 4.5S=S; UCSS1a) | Fruits, Capsicum annuum | KEICCKVPTTPFLCTNDPQCKTLCSKVNYEDGHCFDILSKCVCMNRCVQDAKTLAAELIEEEFLKQ | Anti-Gram negative, Antifungal | 66 | -2 | 43% |
| 234 | Tridecaptin A1 (TriA1; lipopeptides; nonribosomally synthesized peptide antibiotic, Gram-positive bacteria, prokaryotes; 6XXD; UCLL1; BBW; Derivatives: Oct-TriA1; H-TriA1) | Paenibacillus terrae; old: Bacillus circulans NRRL B-30644 | VKGSWSKKFEVIA | Anti-Gram negative | 13 | 2 | 46% |
| 235 | Tridecaptin B1 (TriB1; lipopeptides; nonribosomally synthesized peptide antibiotic, Gram-positive bacteria, prokaryotes, UCLL1) | Paenibacillus polymyxa NRRL B-30507 | GKGSWSKKIEVIA | Anti-Gram negative | 13 | 2 | 38% |
| 236 | NCR247 (nodule-specific Cys-containing peptide; plants; BBMm; UCSS1a) | Medicago truncatula | RNGCIVDPRCPYQQCRRPLYCRRR | Anti-Gram negative | 24 | 6 | 29% |
| 237 | Histone H4 (mollusca/molluscs/mollusks, invertebrates, animals; UCLL1) | American cupped oysters, Crassostrea virginica | MSGRGKGGKGLGKGGAKRHRKVLRDNIQGITKPAIRRLARRGGVKRISGLIYEETRGVLKVFLENVIRDAVTYTEHAKRKTVTAMDVVYALKRQGRTLYGFGG | Anti-Gram negative | 103 | 18 | 33% |
| 238 | Histone H2B (mammals, animals; UCLL1) | calf thymus , Bos taurus | MPEPAKSAPAPKKGSKKAVTKAQKKDGKKRKRSRKESYSVYVYKVLKQVHPDTGISSKAMGIMNSFVNDIFERIAGEASRLAHYNKRSTITSREIQTAVRLLLPGELAKHAVSEGTKAVTKYTSSK | Anti-Gram negative | 126 | 18 | 30% |
| 239 | Histone H3 (mammals, animals; UCLL1) | calf thymus , Bos taurus | MARTKQTARKSTGGKAPRKQLATKAARKSAPATGGVKKPHRYRPGTVALREIRRYQKSTELLIRKLPFQRLVREIAQDFKTDLRFQSSAVMALQEACEAYLVGLFEDTNLCAIHAKRVTIMPKDIQLARRIRGERA | Anti-Gram negative | 136 | 20 | 38% |
| 240 | Histone H4 (mammals, animals; UCLL1) | calf thymus , Bos taurus | MSGRGKGGKGLGKGGAKRHRKVLRDNIQGITKPAIRRLARRGGVKRISGLIYEETRGVLKIFLENVIRDAVTYTEHARRKTVTAMDVVYALKRQGRTLYGFGG | Anti-Gram negative | 103 | 18 | 33% |
| 241 | Acipensin 6 (Ac6, bony fish, animals, UCLL1) | Leukocytes; the Russian Sturgeon, Acipenser gueldenstaedtii | ILELAGNAARDNKKTRIIPRHLQL | Anti-Gram negative | 24 | 3 | 41% |
| 242 | Peptide fraction II (Gly-rich; insects, arthropods, invertebrates, animals, UCLL1) | Hemolymph, Antheraea mylitta | GGGGGGHLVA | Anti-Gram negative | 10 | 0 | 30% |
| 243 | hBD-5 (human beta-defensin 5, hBD5; UCSS1a; primates, mammals, animals; 3S=S) | Homo sapiens | GLDFSQPFPSGEFAVCESCKLGRGKCRKECLENEKPDGNCRLNFLCCRQRI | Anti-Gram negative | 51 | 2 | 37% |
| 244 | hBD-6 (human beta-defensin 6, hBD6; UCSS1a; primates, mammals, animals; 3S=S) | Homo sapiens | FFDEKCNKLKGTCKNNCGKNEELIALCQKSLKCCRTIQPSGSIID | Anti-Gram negative, Chemotactic | 45 | 3 | 37% |
| 245 | mBD-12 (Murine beta-defensin 12, UCSS1a; mouse, mammals, animals; 3S=S) | Mus musculus | CRLGRGKCRRTCIESEKIAGWCKLNFFCCRERI | Anti-Gram negative | 33 | 6 | 45% |
| 246 | mBD-6 (Murine beta-defensin 6, UCSS1a; mouse, mammals, animals; 3S=S) | Mus musculus | CMSYGGSCQRSCNGGFRLGGHCGHPKIRCCRRK | Anti-Gram negative | 33 | 7 | 30% |
| 247 | Tet000 (synthetic) | artificial | GATPEDLNQKLS | Anti-Gram negative | 12 | -1 | 25% |
| 248 | Tet033 (synthetic) | artificial | RRRWWWRRWWRR | Anti-Gram negative | 12 | 7 | 41% |
| 249 | Tet037 (synthetic) | artificial | IVRVAVALRRIR | Anti-Gram negative | 12 | 4 | 66% |
| 250 | Tet091 (synthetic) | artificial | ILKWKWPWWPWRR | Anti-Gram negative | 13 | 4 | 53% |
| 251 | Tet099 (synthetic) | artificial | FIRWRFRWWRWRR | Anti-Gram negative | 13 | 6 | 53% |
| 252 | CHB1 (alpha-hemoglobin subunit A derived, chicken, birds, animals) | blood cells, Gallus gallus | VLSAADKNNVKGIFTKIAGHAEEYGAETLERMFTTYPPTKTY | Anti-Gram negative | 42 | 0 | 33% |
| 253 | CHB2 (alpha-hemoglobin subunit D derived AMP, chicken, birds, animals) | blood cells, Gallus gallus | LTAEDKKLIQQAWEKAASHQEEFGAEALTRMFTTYPQTKTY | Anti-Gram negative | 41 | -1 | 34% |
| 254 | SAAP fraction 2 (Surfactant-associated anionic peptides; Asp-rich, sheep, ruminant, mammals, animals; BBII) | Ovis aries | GDDDDDD | Anti-Gram negative | 7 | -6 | 0% |
| 255 | SAAP fraction 6 (Surfactant-associated anionic peptides; Asp-rich, sheep, ruminant, mammals, animals; BBII) | Ovis aries | GADDDDD | Anti-Gram negative | 7 | -5 | 14% |
| 256 | p138c (bacteriocins; Gram-positive bacteria, prokaryotes) | Bacillus subtilis CSB138 | GLEETVYIYGANMAS | Anti-Gram negative, Anti-MRSA | 15 | -2 | 40% |
| 257 | HE2alpha (human, animals) | male reproductive tract, Homo sapiens | VHISHREARGPSFRICVGFLGPRWARGCSTGN | Anti-Gram negative | 32 | 4 | 47% |
| 258 | HE2beta2 (human, animals) | male reproductive tract , Homo sapiens | GDVPPGIRNTICRMQQGICRLFFCHSGTGQQHRQRCG | Anti-Gram negative | 37 | 4 | 32% |
| 259 | BG-CATH29 (cathelicidin-Bg, frog, amphibians, animals; 1S=S, UCSS1a; Alternative form? BG-CATH25) | skin, Bufu bufo gargarizans, China, Asia | NGKKKRKKPEKLCMKPGACSVIFDASVNE | Anti-Gram negative | 29 | 5 | 34% |
| 260 | Gloverin-like peptide (BWQ; Gly-rich; insects, arthropods, invertebrates, animals) | hemolymph, muga silkworm, Antheraea assamensis, | KSGGGGWGSGGGGGGGGGWWWSGWGVDR | Anti-Gram negative | 28 | 1 | 21% |
| 261 | P10 (UCLL1, rumen microbiome, bacteria) |  | RSITRPVLVRRRWRVRPVF | Anti-Gram negative | 19 | 7 | 42% |
| 262 | P11 (UCLL1, rumen microbiome, bacteria) |  | SIKILKIYFIQGKRHWSF | Anti-Gram negative | 18 | 4 | 44% |
| 263 | P12 (UCLL1, rumen microbiome, bacteria) |  | QVRWWGRYWRRKWATCR | Anti-Gram negative | 17 | 6 | 41% |
| 264 | P15a (UCLL1, rumen microbiome, bacteria) |  | GTAWRWHYRARS | Anti-Gram negative | 12 | 3 | 33% |
| 265 | P15s (UCLL1, rumen microbiome, bacteria) |  | KFVRLKIYCRDKNKGRGISF | Anti-Gram negative | 20 | 6 | 35% |
| 266 | P1 (rumen microbiome, bacteria) |  | THRLRRWCRARGLAR | Anti-Gram negative | 15 | 6 | 4000% |
| 267 | P6 (UCLL1, rumen microbiome, bacteria) |  | LTKKTKKQKRNLVGTT | Anti-Gram negative | 16 | 6 | 18% |
| 268 | P9 (S-S?, rumen microbiome, bacteria) |  | LIRCSRTCLQYKTSRFMRW | Anti-Gram negative | 19 | 5 | 42% |
| 269 | P13 (a second P11 in the original table, #128 in Pep Library, rumen microbiome, bacteria) | | RICRTRLTRRAGNSL | Anti-Gram negative | 15 | 3 | 33% |
| 270 | P16 (UCLL1, rumen microbiome, bacteria) |  | VGVKRRLKCLLSLRS | Anti-Gram negative | 15 | 5 | 46% |
| 271 | P17 (UCLL1, rumen microbiome, bacteria) |  | RRLRTTTKLPPV | Anti-Gram negative | 12 | 4 | 25% |
| 272 | P18 (S-S?, rumen microbiome, bacteria) |  | TTAPCKCWIGLRRCFK | Anti-Gram negative | 16 | 4 | 50% |
| 273 | P19 (UCLL1, rumen microbiome, bacteria) |  | RLLLVMIGLRSKIKWHSGI | Anti-Gram negative | 19 | 4 | 52% |
| 274 | P21 (UCLL1, rumen microbiome, bacteria) |  | SRATWARVRRLGLYG | Anti-Gram negative | 15 | 4 | 40% |
| 275 | P23 (UCLL1, rumen microbiome, bacteria) |  | AVWMTRSCVIWKR | Anti-Gram negative | 13 | 3 | 61% |
| 276 | P24 (UCLL1, rumen microbiome, bacteria) |  | LLMRKLIKGYGYLFGKGKRKKR | Anti-Gram negative | 22 | 9 | 31% |
| 277 | P25 (UCLL1, rumen microbiome, bacteria) |  | MAKLLRLDKKRNKFLCFV | Anti-Gram negative | 18 | 5 | 55% |
| 278 | Tur1A (Dolphin, mammals, animals; Pro-rich; Arg-rich; UCLL1; BBribo) | *Tursiopt truncatus* | RRIRFRPPYLPRPGRRPRFPPPFPIPRIPRIP | Anti-Gram negative | 32 | 10 | 25% |
| 279 | Tur1B (Dolphin, mammals, animals; Pro-rich; UCLL1) | *Tursiopt truncatus* | RRIPFWPPNWPGPWLPPWSPPDFRIPRILRKR | Anti-Gram negative | 32 | 6 | 34% |
| 280 | ocellatin-LB1 (frog, amphibians, animals; XXA, UCLL1c; Ocellatin-LB2) | skin secretion, *Leptodactylus labyrinthicus,* South America | GVVDILKGAAKDIAGHLASKVM | Anti-Gram negative | 22 | 2 | 54% |
| 281 | Distinctin-Like-Peptide-PH (DLP-PH; frog, amphibians, animals; UCLL1a ) | Skin Secretion, *Phyllomedusa hypochondrialis,* South America | NLVSALIEGRKYLKNVLKKLNRLKEKNKAKNSKENN | Anti-Gram negative, Antifungal, Anticancer | 36 | 8 | 30% |
| 282 | Hg-CATH (cathelicidin; rats, rodents, mammals, animals; Lys-rich; UCLL1) | naked mole rat, *Heterocephalus glaber* | RRFRRTVGLSKFFRKARKKLGKGLQKIKNVLRKYLPRPQYAYA | Anti-Gram negative | 43 | 16 | 32% |
| 283 | Oxysterlin 2 (UCSS1a; insects, arthropods, invertebrates, animals) | dung beetle, Oxysternon conspicillatum | GSKRWRKFEKKVKKALEDAKEKLQEERVQKIVEHTKEALPVIKAVATVVGVVGRR | Anti-Gram negative | 55 | 9 | 38% |
| 284 | BmGlv1 (Gloverin, insects, arthropods, invertebrates, animals, UCLL1a) | the silkworm *Bombyx mori* | QVSMPPGYAEKYPITSQFSRSVRHPRDIHDFVTWDREMGGGKVFGTLGESDQGLFGKGGYNREFFNDDRGKLTGQAYGTRVLGPGGDSTSYGGRLDWANENAKAAIDLNRQIGGSAGIEASASGVWDLGKNTHLSAGGVVSKEFGHRRPDVGLQAQITHEW | Anti-Gram negative | 161 | 4 | 29% |
| 285 | BmGlv2 (Gloverin, insects, arthropods, invertebrates, animals, UCLL1a) | the silkworm *Bombyx mor*i | EVYGPSDYAEDYSISGQSSRRHPRDVTWDKQMGGGKVFGTLGQNDDGLFGKAGYNKEIFNDDRGKLTGQAYGTRVLGPGGDSTNYGGRLDWANKNAQATIDLNRQIGGRSGMTASGSGVWDLDKNTHFSAGGMVSKEFGHKRPDVGLQAEIRHDW | Anti-Gram negative | 155 | 3 | 26% |
| 286 | BmGlv3 (Gloverin, insects, arthropods, invertebrates, animals, UCLL1a) | the silkworm *Bombyx mori* | EVYRSSDYEKEYPIRGLFSKRHPRDVTWDTRMGGGKVFGTLGQNDDGLFGKAGYNREIFNDDRGQLTGQAYGTRVLGPGGDSTNYGGRLDWANKNAQAAIDINRQIGGRSGMTASGSGVWDLDKNTHISAGGMVSKEFGHRRPDVGLQAEIRHEW | Anti-Gram negative | 155 | 5 | 27% |
| 287 | BmGlv4 (Gloverin, insects, arthropods, invertebrates, animals, UCLL1a) | the silkworm *Bombyx mori* | EVYSEYEEGYPISGQFSKRHPRDVTWDKQVGGGKVFGTLGQNDDGLFGKAGYNREIFNDDRGKLTGQAYGTRVLGPAGDSTNYGGRLDWANKNAEAAIDINRQIGGRSGMTATGSGVWDLDKNTRLSAGGMISKEFGHRRPDVGVQAEFRHDW | Anti-Gram negative | 153 | 2 | 28% |
| 288 | Dermaseptin-PS3 (DPS3, XXA, UCLL1c; frog, amphibians, animals) | skin secretion, *Phyllomedusa sauvagii,* USA, North America | ALWKDILKNAGKAALNEINQIVQ | Anti-Gram negative, Antifungal | 23 | 2 | 52% |
| 289 | Lucilin (insects, arthropods, invertebrates, animals) | Lucilia eximia | GWLKKLGKKIERVGQHTRDATIQTIGVAQQAVNVAATLKG | Anti-Gram negative, Chemotactic, Anti-inflammatory | 40 | 6 | 40% |
| 290 | OvoDBbeta (chicken Ovodefensin, UCSS1a; 3S=S, birds, animals) | *Gallus gallus* | QSKKCCGRCSSRMCTKREKEEHTEDCRGSFCCLTHRKKK | Anti-Gram negative | 39 | 9 | 25% |
| 291 | gcDefb1 (grass carp beta-defensin 1; 3S=S; UCSS1a; fish, animals) | brain and liver; grass carp, Ctenopharyngodon idella | MKPQSILVLLVLAVLALHCKENEAASFPWTCASLSGVCRQGVCLPSELYFGSLGCGKGFLCCVSHFG | Anti-Gram negative, Anti-inflammatory | 67 | 3 | 53% |
| 292 | HRNR1132–1143 (Hornerin-derived Ser-rich; human; primates; mammals; animals; CIDAMP; UCLL1a; more AMPs?) | skin, *Homo sapiens* | GSGSRQSPSYGR | Anti-Gram negative | 12 | 2 | 0% |
| 293 | Arenicin-3 (Ar-3, NZ17000; UCSS1a; 1S=S, marine polychaeta, annelid, invertebrates, animals; derivatives: NZ17074) | the lugworm*, Arenicola marina* | GFCWYVCYRNGVRVCYRRCN | Anti-Gram negative | 20 | 4 | 45% |
| 294 | SPINK9-v1 (serine peptidase inhibitor, Kazal type 9, variant 1, humans; primates, mammals, animals; UCSS1a) | stratum corneum, skin, *Homo sapiens* | KQTKQMVDCSHYKKLPPGQQRFCHHMYDPICGSDGKTYKNDCFFCSKVKKTDGTLKFVHFGKC | Anti-Gram negative, Enzyme inhibitor | 63 | 11 | 30% |
| 295 | TroCCL4 (chemokine, CC family; kinocidins; fish, animals; UCSS1a) | golden pompano, *Trachinotus ovatus* | MAAPRLTLSVFVLMLAFITLSEGLRGTGPKKCCFRFFESPVQKERVLSYIKTSQRCPQPAVLLKTVAGRQLCAKPSASWVKDLISYLDAKPGEVSN | Anti-Gram negative, Chemotactic | 96 | 8 | 44% |
| 296 | PR-35 (a proline-arginine-rich peptide with 35 residues, cathelicidin, pigs, mammals; animals; Pro-rich; Arg-rich) | Sus scrofa | RPPYLPRPRPPPFFPPRLPPRIPPGFPPRFPPRFP | Anti-Gram negative | 35 | 7 | 22% |
| 297 | NEMURI (NUR; insects, arthropods, invertebrates, animals; Arg-rich) | Drosophila melanogaster | DARARRIVRAGRRRGGRRGGRRGGRRSARKS | Anti-Gram negative | 31 | 14 | 19% |
| 298 | Citrocin (Gly-rich; lactam; a class 2 lasso peptide; class 1 microcins, bacteriocins, Gram-negative bacteria, prokaryotes; XXJ; UCSB1a; BBpol) | Citrobacter pasteurii and Citrobacter braakii | GGVGKIIEYFIGGGVGRYG | Anti-Gram negative | 19 | 1 | 31% |
| 299 | Gm cecropin A (CecA, insects, arthropods, invertebrates, animals) | *Galleria mellonella* | KWKIFKKIEKAGRNIRDGIIKAGPAVSVVGEAATIYKTG | Anti-Gram negative | 39 | 6 | 41% |
| 300 | IL-26 (interleukin 26, humans; primates, mammals, animals; 3S=S, UCSS1a) | Th17 cells, *Homo sapiens* | MLVNFILRCGLLLVTLSLAIAKHKQSSFTKSCYPRGTLSQAVDALYIKAAWLKATIPEDRIKNIRLLKKKTKKQFMKNCQFQEQLLSFFMEDVFGQLQLQGCKKIRFVEDFHSLRQKLSHCISCASSAREMKSITRMKRIFYRIGNKGIYKAISELDILLSWIKKLLESSQ | Anti-Gram negative | 171 | 19 | 44% |
| 301 | PDB114 (porcine beta-defensin 114, UCSS1a; 3S=S, pigs, mammals; animals) | Sus scofa | TLVDPERCSKMYGQCRTRCYKIEKQIDICYSPSKICCIQRAFEEDLS | Anti-Gram negative | 47 | 1 | 36% |
| 302 | Cter E (cyclotides; XXC; 3S=S, UCBB1b; plants) | *Clitoria ternatea* | GIPCAESCVWIPCTVTALLGCSCKDKVCYLD | Anti-Gram negative | 31 | -1 | 54% |
| 303 | Cter G (cyclotides; XXC; 3S=S, UCBB1b; plants) | *Clitoria ternatea* | GLPCGESCVFIPCITTVVGCSCKNKVCYNN | Anti-Gram negative | 30 | 1 | 46% |
| 304 | LaIT3 (scorpion toxins, arachnids, Chelicerata, arthropods, invertebrates, animals; 3S=S, UCSS1a) | venom, Liocheles australasiae, Japan, Asia | GGILREKYFHKAADALTSNIPIPVVKDVLKSAANQMIRKIGKVQQACAFNKDLAGWCEKSCQEAEGKKGYCHGTKCKCGKPIDY | Anti-Gram negative, Insecticidal | 84 | 9 | 39% |
| 305 | LaIT2 (scorpion toxins, arachnids, Chelicerata, arthropods, invertebrates, animals; 3S=S, UCSS1a; modular design) | venom, *Liocheles australasiae*, Japan, Asia | AKKPFVQRVKNAASKAYNKLKGLAMQSQYGCPIISNMCEDHCRRKKMEGQCDLLDCVCS | Anti-Gram negative, Insecticidal | 59 | 7 | 40% |
| 306 | S100A15 (Koebnerisin; metal-binding protein, BBII; humans; primates, mammals, animals) | skin, *Homo sapiens* | MSNTQAERSIIGMIDMFHKYTGRDGKIEKPSLLTMMKENFPNFLSACDKKGIHYLATVFEKKDKNEDKKIDFSEFLSLLGDIAADYHKQSHGAAPCSGGSQ | Anti-Gram negative | 101 | 4 | 34% |
| 307 | Temporin B (temporin-1Tb; temporin-Tb; TB; Leu-rich; XXA, UCLL1c; frog, amphibians, animals; ZZP) | European common frog, *Rana temporaria* | LLPIVGNLLKSLL | Anti-Gram positive & Gram negative, Antiviral, Antiparasitic, Chemotactic, Antibiofilm | 13 | 2 | 61% |
| 308 | Indolicidin (IR13; Tet083; XXA, Trp-rich, bovine cathelicidin, cattle, ruminant, mammals; animals; BBN; BBPP/BBII; Derivatives: CP-11, MBI-549, Omiganan pentahydrochloride (formerly MBI 226; MBI-226 | bovine neutrophils, *Bos taurus* | ILPWKWPWWPWRR | Anti-Gram positive & Gram negative, Antiviral, Antifungal, Anti-HIV, Anti-MRSA, Hemolytic, Antibiofilm, Wound healing | 13 | 4 | 53% |
| 309 | SMAP-29 (SMAP29, sheep myeloid AMP-29; SMAP-28, OaMAP28, ovine cathelicidin, sheep, ruminant, mammals, animals; BBomp; BBL; derivatives: Ovispirin, OV-1, OV-2, OV-3, novispirin, novici | sheep leukocytes; *Ovis aries* | RGLRRLGRKIAHGVKKYGPTVLRIIRIAG | Anti-Gram positive & Gram negative, Antifungal, Anti-MRSA, Hemolytic, Antibiofilm | 29 | 9 | 37% |
| 310 | Pleurocidin (NRC-4, NRC-04; WF2; XXA; fish, animals, UCLL1) | the skin mucous secretions, Winter flounder, *Pleuronectes americanus* | GWGSFFKKAAHVGKHVGKAALTHYL | Anti-Gram positive & Gram negative, Antifungal, Chemotactic, Anti-MRSA, Antibiofilm | 25 | 4 | 44% |
| 311 | Protegrin 1 (Protegrin-1, PG-1, IB-200; UCSS1a; cathelicidin, pigs, mammals; animals; XXA, ZZHa, BBBm; Derivatives: Iseganan, protegrin IB 367 (IB-367); Clinical; BBL; JJsn; 2S=S) | leukocytes; Pig, *Sus scrofa* | RGGRLCYCRRRFCVCVGR | Anti-Gram positive, Antiviral, Antifungal, Anti-HIV, Anti-MRSA, Antibiofilm, | 18 | 7 | 44% |
| 312 | Nisin A (NisaplinTM, ChrisinTM, food additive E234; lantibiotic, type 1, class 1 bacteriocin, Gram-positive bacteria, prokaryotes; XXT5; XXW3; UCSS1b; ZZS; BBW; BBMm; JJsn; Variants: nisin AP) | *Streptococcus lactis,* reclassified as *Lactococcus lactis* | ITSISLCTPGCKTGALMGCNMKTATCHCSIHVSK | Anti-Gram positive, Spermicidal, Antibiofilm, Wound healing, Anticancer | 34 | 3 | 44% |
| 313 | Tachyplesin III (XXA, UCSS1a; 2S=S; Horseshoe Crab, arachnids, Chelicerata, arthropods, invertebrates, animals) | Southeast Asian, *Tachypleus gigas* | KWCFRVCYRGICYRKCR | Anti-Gram positive & Gram negative, Antibiofilm | 17 | 7 | 47% |
| 314 | Human beta defensin 3 (hBD-3, hBD3, or DEFB103, human defensin, 3S=S, UCSS1a; primates, mammals, animals; ZZHh; BBBh2o; BBW; JJsn) | skin, tonsils, oral/saliva, *Homo sapiens* | GIINTLQKYYCRVRGGRCAVLSCLPKEEQIGKCSTRGRKCCRRKK | Anti-Gram positive & Gram negative, Antiviral, Antifungal, Anti-HIV, Chemotactic, Anti-MRSA, Anti-toxin, Antibiofilm, Wound healing, Anticancer | 45 | 11 | 33% |
| 315 | LL-37 [LL37; FALL-39; cathelicidin; UCLL1; human; chimpanzee; primates, mammals, animals; XXX; XXY; XXZ; BBBh2o, BBBm; BBMm, BBPP, BBN, BBL, BBrsg, JJsn; Derivatives: many) | neutrophils, monocytes; mast cells; lymphocytes, Mesenchymal Stem Cells; islets; skin, sweat; airway surface liquid, saliva; *Homo sapiens*; Also *Pan troglodytes* | LLGDFFRKSKEKIGKEFKRIVQRIKDFLRNLVPRTES | Anti-Gram positive & Gram negative, Antiviral, Antifungal, Antiparasitic, Spermicidal, Anti-HIV, Chemotactic, Anti-MRSA, Enzyme inhibitor, Hemolytic, Antibiofilm, Wound healing, Anticancer | 37 | 6 | 35% |
| 316 | Citropin 1.1 (UCLL1c; XXA, frog, amphibians, animals) | Australian blue mountains tree frog, *Litoria citropa* | GLFDVIKKVASVIGGL | Anti-Gram positive, Antifungal, Anti-MRSA, Antibiofilm, Anticancer | 16 | 2 | 56% |
| 317 | BMAP-27 (BMAP27, bovine myeloid antimicrobial peptide 27; bovine cathelicidin, cattle, ruminant, mammals, animals; ZZHs; ZZP; UCLL1; Derivatives: BMAP-18 and BMAP-15) | Cow *Bos taurus* | GRFKRFRKKFKKLFKKLSPVIPLLHLG | Anti-Gram positive & Gram negative, Antiviral, Antifungal, Antiparasitic, Anti-HIV, Anti-MRSA, Hemolytic, Antibiofilm, Anticancer | 27 | 10 | 40% |
| 318 | BMAP-28 (BMAP28, bovine myeloid antimicrobial peptide 28; bovine cathelicidin-5, cattle, ruminant, mammals, animals; BBMm;ZZP; UCLL1; Derivatives: mBMAP-28 | Cow *Bos taurus* | GGLRSLGRKILRAWKKYGPIIVPIIRIG | Anti-Gram positive & Gram negative, Antiviral, Antifungal, Antiparasitic, Anti-MRSA, Hemolytic, Antibiofilm, Anticancer | 28 | 7 | 42% |
| 319 | Agelaia-MP (Agelaia-MP-I; insects, arthropods, invertebrates, animals; XXA) | social wasp, *Agelaia pallipes pallipes* | INWLKLGKAIIDAL | Anti-Gram positive & Gram negative, Hemolytic, Antibiofilm | 14 | 2 | 64% |
| 320 | Chicken CATH-2 (chicken cathelicidin 2; CMAP27, chicken myeloid antimicrobial peptide 27, Fowlicidin-2; chCATH-2; birds, animals; BBL; Derivatives: F2,5,12W) | *Gallus gallus* | RFGRFLRKIRRFRPKVTITIQGSARFG | Anti-Gram positive & Gram negative, Anti-MRSA, Hemolytic, Antibiofilm | 27 | 9 | 37% |
| 321 | Temporin 1OLa (XXA, Temporin-1OLa, UCLL1c; frog, amphibians, animals. More AMPs?) | Florida bog frog, *Rana okaloosae*, North America | FLPFLKSILGKIL | Anti-Gram positive, Antifungal, Anti-MRSA, Antibiofilm, Anticancer | 13 | 3 | 61% |
| 322 | NA-CATH (N. atra cathelicidin; snake, reptiles, animals) | *Naja atra* | KRFKKFFKKLKNSVKKRAKKFFKKPKVIGVTFPF | Anti-Gram positive & Gram negative, Antibiofilm | 34 | 15 | 38% |
| 323 | Temporin-PTa (XXA; frog, amphibians, animals: ZZH; UCLL1c; Derivatives: DASamP1) | *Hylarana picturata*, Asia | FFGSVLKLIPKIL | Anti-Gram positive & Gram negative, Antiviral, Anti-HIV, Anti-MRSA, Antibiofilm | 13 | 3 | 61% |
| 324 | Myxinidin (hagfish, fish, animals; XXA; UCLL1; Derivatives: Myxinidin1; Myxinidin2; Myxinidin3; WMR) | Epidermal mucus, *Myxine glutinosa L.* | GIHDILKYGKPS | Anti-Gram positive & Gram negative, Antifungal, Antibiofilm, Wound healing | 12 | 2 | 25% |
| 325 | Phylloseptin-1 (PSN-1, UCLL1c; frog, amphibians, animals; XXA) | the waxy monkey frog, *Phyllomedusa sauvagei*, South America | FLSLIPHIVSGVASIAKHF | Anti-Gram positive & Gram negative, Antifungal, Antibiofilm | 19 | 2 | 57% |
| 326 | Polybia-MP-II (Polybia-MPII; insects, arthropods, invertebrates, animals; XXA) | venom, social wasp, *Polybia paulista*; Also *Pseudopolybia vespiceps testacea* | INWLKLGKMVIDAL | Anti-Gram positive & Gram negative, Antifungal, Chemotactic, Hemolytic, Antibiofilm | 14 | 1 | 64% |
| 327 | Coprisin (defensin-like peptide; UCSS1a; 3S=S, insects, arthropods, invertebrates, animals; Derivatives: CopA3) | Dung Beetle, *Copris tripartitus.* | VTCDVLSFEAKGIAVNHSACALHCIALRKKGGSCQNGVCVCRN | Antifungal, Anti-inflammatory, Antibiofilm, Wound healing | 43 | 3 | 51% |
| 328 | Datucin (glycopeptide; plants; XXG; XXK) | *Datura stramonium*, India, Asia | TFPKCAPTRPPGPKPCDINNFKSKFWHIWRA | Antifungal, Antibiofilm | 31 | 5 | 35% |
| 329 | CCL20 (macrophage inflammatory protein-3alpha, MIP-3alpha; Liver and activation-regulated chemokine, LARC; CC family; UCSS1a; 2S=S, humans; primates, mammals, animals) | Skin, *Homo sapiens* | SNFDCCLGYTDRILHPKFIVGFTRQLANEGCDINAIIFHTKKKLSVCANPKQTWVKYIVRLLSKKVKNM | Anti-Gram positive & Gram negative, Antifungal, Antiparasitic, Chemotactic, Antibiofilm, | 69 | 8 | 43% |
| 330 | UyCT3 (OcyC1; NDBP-5.7; scorpions, arachnids, Chelicerata, arthropods, invertebrates, animals; XXA, UCLL1c) | venom, *Urodacus yaschenkoi,* Australia; also *Opisthacanthus cayaporum* | ILSAIWSGIKSLF | Anti-Gram positive & Gram negative, Antifungal, Antibiofilm, | 13 | 2 | 61% |
| 331 | TsAP-2 (NDBP-4.23, T. serrulatus antimicrobial peptide 2; scorpions, arachnids, Chelicerata, arthropods, invertebrates, animals; UCLL1a) | venom, the Brazilian yellow scorpion, *Tityus serrulatus,* also Turrilites costatus; Tityus obscurus | FLGMIPGLIGGLISAFK | Anti-Gram positive, Antifungal, Antibiofilm, Anticancer | 17 | 1 | 58% |
| 332 | Gramicidin S (Gramicidin Soviet, GS; nonribosomally synthesized peptide antibiotic; bacteria; prokaryotes; XXC; XXD2; UCBB1a; BBMm; JJsn) | *Bacillus brevis* | VKLFPVKLFP | Anti-Gram positive & Gram negative, Antifungal, Spermicidal, Hemolytic, Antibiofilm | 10 | 2 | 60% |
| 333 | Enterocin O16 (EntV; bacteriocins; Gram-positive bacteria, prokaryotes) | *Enterococcus faecalis* | LGSCVANKIKDEFFAMISISAIVKAAQKKAWKELAVTVLRFAKANGLKTNAIIVAGQLALWAVQCGLS | Anti-Gram positive, Antifungal, Antibiofilm | 68 | 6 | 58% |
| 334 | GL13K (a derivative of GL13NH2; BBL; BBMm; XXA; UCLL1c; synthetic) | a synthetic peptide derived from human Parotid secretory protein | GKIIKLKASLKLL | Anti-Gram positive & Gram negative, Anti-inflammatory, Antibiofilm | 13 | 5 | 53% |
| 335 | Holothuroidin 1 (Echinoderm; animals; UCLL1a) | sea-cucumber, *Holothuria tubulosa* | HLGHHALDHLLK | Anti-Gram positive & Gram negative, Antibiofilm | 12 | 0 | 41% |
| 336 | Holothuroidin 2 (H2, Echinoderm; animals; UCLL1a) | sea-cucumber, *Holothuria tubulosa* | ASHLGHHALDHLLK | Anti-Gram positive & Gram negative, Antibiofilm | 14 | 0 | 42% |
| 337 | Paracentrin 1 (SP1, Echinoderm, animals; UCLL1a) | the coelomocyte cytosol, the sea urchin, *Paracentrotus lividus* | EVASFDKSKLK | Anti-Gram positive & Gram negative, Antibiofilm | 11 | 1 | 36% |
| 338 | ToAP1 (scorpions, arachnids, Chelicerata, arthropods, invertebrates, animals; XXA; UCLL1c) | *Tityus obscurus* | FIGMIPGLIGGLISAFK | Antifungal, Antibiofilm | 17 | 2 | 58% |
| 339 | ToAP2 (scorpions, arachnids, Chelicerata, arthropods, invertebrates, animals; UCLL1a) | *Tityus obscurus* | FFGTLFKLGSKLIPGVMKLFSKKKER | Antifungal, Antibiofilm | 26 | 6 | 42% |
| 340 | Con10 (scorpions, arachnids, Chelicerata, arthropods, invertebrates, animals; UCLL1a) | *Opisthacanthus cayaporum* | FWSFLVKAASKILPSLIGGGDDNKSSS | Antifungal, Antibiofilm | 27 | 1 | 40% |
| 341 | TetraF2W-RK (W312; Trp-rich; synthetic; BBmM; UCLL1a; XXA) | artificial, designed based on temporin-SHf | WWWLRKIW | Anti-Gram positive & Gram negative, Antifungal, Anti-MRSA, Antibiofilm | 8 | 3 | 75% |
| 342 | H4 (synthetic, a hybrid peptide containing OP-145) | artificial, Combined BMAP-27 and OP-145 | KFKKLFKKLSPVIGKEFKRIVERIKRFLR | Anti-Gram positive & Gram negative, Anti-MRSA, Antibiofilm | 29 | 10 | 41% |
| 343 | Phylloseptin-Co (PS-Co; XXA; frog, amphibians, animals, UCLL1c) | *Phyllomedusa coelestis*, South America | FLSMIPKIAGGIASLVKNL | Anti-Gram positive, Antifungal, Antibiofilm | 19 | 3 | 57% |
| 344 | Esculentin 1-21 (Esc 1-21; synthetic; BBMm; XXA; UCLL1c) | artificial, template derived | GIFSKLAGKKIKNLLISGLKG | Anti-Gram positive & Gram negative, Antifungal, Chemotactic, Antibiofilm, Wound healing, | 21 | 6 | 42% |
| 345 | SAAP-148 (synthetic) | artificial, designed based on LL-37 | LKRVWKRVFKLLKRYWRQLKKPVR | Anti-Gram positive & Gram negative, Antibiofilm | 24 | 11 | 41% |
| 346 | Dhvar4 (synthetic) | artificial | KRLFKKLLFSLRKY | Anti-Gram positive & Gram negative, Antifungal, Antibiofilm | 9 | 3 | 66% |
| 347 | Dermaseptin-PH (Dermaseptin PH; frog, amphibians, animals; XXA; ; UCSS1c) | orange-legged leaf frog, *Pithecopus (Phyllomedusa) hypochondrialis*, South America | ALWKEVLKNAGKAALNEINNLV | Anti-Gram positive & Gram negative, Antifungal, Antibiofilm, Anticancer | 22 | 2 | 54% |
| 348 | Hyicin 4244 (XXC; sactipeptide; 3S=CA; UCSB1c; lantibiotic, class 2c bacteriocin, Gram-positive bacteria, prokaryotes) | *Staphylococcus hyicus 4244* | NKGCSACAIGAACLADGPIPDFEVAGITGTFGIAS | Anti-Gram positive, Antibiofilm | 35 | -2 | 51% |
| 349 | Moronecidin-like (seahorse, fish, animals; UCLL1a) | Tiger tail seahorse, *Hippocampus comes* | FFRNLWKGAKAAFRAGHAAWRA | Anti-Gram positive & Gram negative, Antifungal, Anti-MRSA, Antibiofilm | 22 | 6 | 59% |
| 350 | MP-C (mastoporan-C, insects, arthropods, invertebrates, animals. XXA; UCLL1c) | venom, the European Hornet, *Vespa crabro* | LNLKALLAVAKKIL | Anti-Gram positive & Gram negative, Antifungal, Anti-MRSA, Hemolytic, Antibiofilm, Anticancer | 14 | 4 | 71% |
| 351 | Nigrocin-HLM (synthetic, XXA, UCLL1c) | Motif-Targeted Peptide Design | GLLSGILGAGKKIVF | Anti-Gram positive & Gram negative, Antifungal, Anti-MRSA, Antibiofilm | 15 | 2 | 53% |
| 352 | VLL-28 (archaeocins, archaea, prokaryote; UCLL1) | *Sulfolobus islandicus* | VLLVTLTRLHQRGVIYRKWRHFSGRKYR | Anti-Gram positive & Gram negative, Antifungal, Antibiofilm, Anticancer | 28 | 10 | 35% |
| 353 | Phylloseptin-PHa (PSPHa, XXA; UCLL1c; frog, amphibians, animals) | Orange-legged Leaf Frog, *Pithecopus hypochondrialis*, South America | FLSLIPAAISAVSALANHF | Anti-Gram positive, Antifungal, Anti-MRSA, Antibiofilm, Anticancer | 19 | 2 | 68% |
| 354 | Japonicin-2LF (frog, amphibians, animals; XXU; 1S=S, UCSS1a; BBMm) | skin secretion, Fujian Large-headed Frog, Limnonectes fujianensis, China, Asia | FIVPSIFLLKKAFCIALKKC | Anti-Gram positive & Gram negative, Antifungal, Anti-MRSA, Antibiofilm | 20 | 4 | 70% |
| 355 | ZmD32 (Z. mays defensin, plants; 4S=S; UCSS1a) | Corn, *Zea mays* | RTCQSQSHRFRGPCLRRSNCANVCRTEGFPGGRCRGFRRRCFCTTHC | Anti-Gram positive & Gram negative, Antifungal, Antibiofilm | 47 | 12 | 31% |
| 356 | SA-CATH (S. annularis cathelicidin; snake, reptiles, animals; Lys-rich; UCLL1a) | *Sinonatrix annularis*, China, Asia | KFFKKLKKSVKKHVKKFFKKPKVIGVSIPF | Anti-Gram positive & Gram negative, Antifungal, Anti-inflammatory, Antibiofilm | 30 | 13 | 40% |
| 357 | Hs02 (synthetic, XXA, UCLL1c) | predicted intragenic AMP | KWAVRIIRKFIKGFIS | Anti-Gram positive & Gram negative, Antifungal, Anti-inflammatory, Antibiofilm | 16 | 6 | 56% |
| 358 | Dermaseptin-PT9 (DPT9, UCLL1a; frog, amphibians, animals) | skin secretion, *Phyllomedusa tarsius,* Purchased in Peru, South America | GLWSKIKDAAKTAGKAALGFVNEMV | Anti-Gram positive & Gram negative, Antifungal, Anti-MRSA, Antibiofilm, Anticancer | 25 | 2 | 52% |
