# Supplementary material for: The antimicrobial peptide EM86 loaded to gamma-irradiated sodium alginate/polyvinyl alcohol electrospun nanofibrous dressing treated multidrug-resistant Pseudomonas aeruginosa wound infections in BALB/c mice
Source: Front Bioeng Biotechnol. 2026 Apr 7;14:1776154. doi: 10.3389/fbioe.2026.1776154 (PMC13095823; doi:10.3389/fbioe.2026.1776154)
Supplement: Supplementary file 3 [file Table2.docx]

Supplementary Table S2. The sequences, net charge and hydrophobicity percentage of the 40 AMPs with anti-Gram negative and anti-biofilm activity

|  | **Peptide name / Class** | **Source** | **Sequence** | **Activity** | **length** | **Net charge** | **Hydrophobicity** |
| --- | --- | --- | --- | --- | --- | --- | --- |
| 1 | Colistin (Polymyxin E1 and E2; lactam, XXD; XXL; XXJ; UCSB1a; lipopeptides; nonribosomally synthesized peptide antibiotic; bacteria; BBL; BBMm, prokaryotes) | Paenibacillus polymyxa var. colistinus; Also known as *Bacillus polymyxa* | KTKKKLLKKT | Anti-Gram negative & antibiofilm | 10 | 6 | 20% |
| 2 | Polymyxin B (aerosporin; lactam, XXD; XXL; XXJ; UCSB1a; lipopeptides; nonribosomally synthesized peptide antibiotic; bacteria; BBL; BBMm, prokaryotes; Variants: Polymyxin B, a mixture) | *Bacillus aerosporus Greer* | KTKKKFLKKT | Anti-Gram negative, antibiofilm & antifungal | 10 | 6 | 20% |
| 3 | Temporin B (temporin-1Tb; temporin-Tb; TB; Leu-rich; XXA, UCLL1c; frog, amphibians, animals; ZZP) | European common frog, *Rana temporaria* | LLPIVGNLLKSLL | Anti-Gram positive & Gram negative, Antiviral, Antiparasitic, Chemotactic, Antibiofilm | 13 | 2 | 61% |
| 4 | Indolicidin (IR13; Tet083; XXA, Trp-rich, bovine cathelicidin, cattle, ruminant, mammals; animals; BBN; BBPP/BBII; Derivatives: CP-11, MBI-549, Omiganan pentahydrochloride (formerly MBI 226; MBI-226 | bovine neutrophils, *Bos taurus* | ILPWKWPWWPWRR | Anti-Gram positive & Gram negative, Antiviral, Antifungal, Anti-HIV, Anti-MRSA, Hemolytic, Antibiofilm, Wound healing | 13 | 4 | 53% |
| 5 | SMAP-29 (SMAP29, sheep myeloid AMP-29; SMAP-28, OaMAP28, ovine cathelicidin, sheep, ruminant, mammals, animals; BBomp; BBL; derivatives: Ovispirin, OV-1, OV-2, OV-3, novispirin, novici | sheep leukocytes; *Ovis aries* | RGLRRLGRKIAHGVKKYGPTVLRIIRIAG | Anti-Gram positive & Gram negative, Antifungal, Anti-MRSA, Hemolytic, Antibiofilm | 29 | 9 | 37% |
| 6 | Pleurocidin (NRC-4, NRC-04; WF2; XXA; fish, animals, UCLL1) | the skin mucous secretions, Winter flounder, *Pleuronectes americanus* | GWGSFFKKAAHVGKHVGKAALTHYL | Anti-Gram positive & Gram negative, Antifungal, Chemotactic, Anti-MRSA, Antibiofilm | 25 | 4 | 44% |
| 7 | Tachyplesin III (XXA, UCSS1a; 2S=S; Horseshoe Crab, arachnids, Chelicerata, arthropods, invertebrates, animals) | Southeast Asian, *Tachypleus gigas* | KWCFRVCYRGICYRKCR | Anti-Gram positive & Gram negative, Antibiofilm | 17 | 7 | 47% |
| 8 | Human beta defensin 3 (hBD-3, hBD3, or DEFB103, human defensin, 3S=S, UCSS1a; primates, mammals, animals; ZZHh; BBBh2o; BBW; JJsn) | skin, tonsils, oral/saliva, *Homo sapiens* | GIINTLQKYYCRVRGGRCAVLSCLPKEEQIGKCSTRGRKCCRRKK | Anti-Gram positive & Gram negative, Antiviral, Antifungal, Anti-HIV, Chemotactic, Anti-MRSA, Anti-toxin, Antibiofilm, Wound healing, Anticancer | 45 | 11 | 33% |
| 9 | LL-37 [LL37; FALL-39; cathelicidin; UCLL1; human; chimpanzee; primates, mammals, animals; XXX; XXY; XXZ; BBBh2o, BBBm; BBMm, BBPP, BBN, BBL, BBrsg, JJsn; Derivatives: many) | neutrophils, monocytes; mast cells; lymphocytes, Mesenchymal Stem Cells; islets; skin, sweat; airway surface liquid, saliva; *Homo sapiens*; Also *Pan troglodytes* | LLGDFFRKSKEKIGKEFKRIVQRIKDFLRNLVPRTES | Anti-Gram positive & Gram negative, Antiviral, Antifungal, Antiparasitic, Spermicidal, Anti-HIV, Chemotactic, Anti-MRSA, Enzyme inhibitor, Hemolytic, Antibiofilm, Wound healing, Anticancer | 37 | 6 | 35% |
| 10 | BMAP-27 (BMAP27, bovine myeloid antimicrobial peptide 27; bovine cathelicidin, cattle, ruminant, mammals, animals; ZZHs; ZZP; UCLL1; Derivatives: BMAP-18 and BMAP-15) | Cow *Bos taurus* | GRFKRFRKKFKKLFKKLSPVIPLLHLG | Anti-Gram positive & Gram negative, Antiviral, Antifungal, Antiparasitic, Anti-HIV, Anti-MRSA, Hemolytic, Antibiofilm, Anticancer | 27 | 10 | 40% |
| 11 | BMAP-28 (BMAP28, bovine myeloid antimicrobial peptide 28; bovine cathelicidin-5, cattle, ruminant, mammals, animals; BBMm;ZZP; UCLL1; Derivatives: mBMAP-28 | Cow *Bos taurus* | GGLRSLGRKILRAWKKYGPIIVPIIRIG | Anti-Gram positive & Gram negative, Antiviral, Antifungal, Antiparasitic, Anti-MRSA, Hemolytic, Antibiofilm, Anticancer | 28 | 7 | 42% |
| 12 | Agelaia-MP (Agelaia-MP-I; insects, arthropods, invertebrates, animals; XXA) | social wasp, *Agelaia pallipes pallipes* | INWLKLGKAIIDAL | Anti-Gram positive & Gram negative, Hemolytic, Antibiofilm | 14 | 2 | 64% |
| 13 | Chicken CATH-2 (chicken cathelicidin 2; CMAP27, chicken myeloid antimicrobial peptide 27, Fowlicidin-2; chCATH-2; birds, animals; BBL; Derivatives: F2,5,12W) | *Gallus gallus* | RFGRFLRKIRRFRPKVTITIQGSARFG | Anti-Gram positive & Gram negative, Anti-MRSA, Hemolytic, Antibiofilm | 27 | 9 | 37% |
| 14 | NA-CATH (N. atra cathelicidin; snake, reptiles, animals) | *Naja atra* | KRFKKFFKKLKNSVKKRAKKFFKKPKVIGVTFPF | Anti-Gram positive & Gram negative, Antibiofilm | 34 | 15 | 38% |
| 15 | Temporin-PTa (XXA; frog, amphibians, animals: ZZH; UCLL1c; Derivatives: DASamP1) | *Hylarana picturata*, Asia | FFGSVLKLIPKIL | Anti-Gram positive & Gram negative, Antiviral, Anti-HIV, Anti-MRSA, Antibiofilm | 13 | 3 | 61% |
| 16 | Myxinidin (hagfish, fish, animals; XXA; UCLL1; Derivatives: Myxinidin1; Myxinidin2; Myxinidin3; WMR) | Epidermal mucus, *Myxine glutinosa L.* | GIHDILKYGKPS | Anti-Gram positive & Gram negative, Antifungal, Antibiofilm, Wound healing | 12 | 2 | 25% |
| 17 | Phylloseptin-1 (PSN-1, UCLL1c; frog, amphibians, animals; XXA) | the waxy monkey frog, *Phyllomedusa sauvagei*, South America | FLSLIPHIVSGVASIAKHF | Anti-Gram positive & Gram negative, Antifungal, Antibiofilm | 19 | 2 | 57% |
| 18 | Polybia-MP-II (Polybia-MPII; insects, arthropods, invertebrates, animals; XXA) | venom, social wasp, *Polybia paulista*; Also *Pseudopolybia vespiceps testacea* | INWLKLGKMVIDAL | Anti-Gram positive & Gram negative, Antifungal, Chemotactic, Hemolytic, Antibiofilm | 14 | 1 | 64% |
| 19 | CCL20 (macrophage inflammatory protein-3alpha, MIP-3alpha; Liver and activation-regulated chemokine, LARC; CC family; UCSS1a; 2S=S, humans; primates, mammals, animals) | Skin, *Homo sapiens* | SNFDCCLGYTDRILHPKFIVGFTRQLANEGCDINAIIFHTKKKLSVCANPKQTWVKYIVRLLSKKVKNM | Anti-Gram positive & Gram negative, Antifungal, Antiparasitic, Chemotactic, Antibiofilm, | 69 | 8 | 43% |
| 20 | UyCT3 (OcyC1; NDBP-5.7; scorpions, arachnids, Chelicerata, arthropods, invertebrates, animals; XXA, UCLL1c) | venom, *Urodacus yaschenkoi,* Australia; also *Opisthacanthus cayaporum* | ILSAIWSGIKSLF | Anti-Gram positive & Gram negative, Antifungal, Antibiofilm, | 13 | 2 | 61% |
| 21 | Gramicidin S (Gramicidin Soviet, GS; nonribosomally synthesized peptide antibiotic; bacteria; prokaryotes; XXC; XXD2; UCBB1a; BBMm; JJsn) | *Bacillus brevis* | VKLFPVKLFP | Anti-Gram positive & Gram negative, Antifungal, Spermicidal, Hemolytic, Antibiofilm | 10 | 2 | 60% |
| 22 | GL13K (a derivative of GL13NH2; BBL; BBMm; XXA; UCLL1c; synthetic) | a synthetic peptide derived from human Parotid secretory protein | GKIIKLKASLKLL | Anti-Gram positive & Gram negative, Anti-inflammatory, Antibiofilm | 13 | 5 | 53% |
| 23 | Holothuroidin 1 (Echinoderm; animals; UCLL1a) | sea-cucumber, *Holothuria tubulosa* | HLGHHALDHLLK | Anti-Gram positive & Gram negative, Antibiofilm | 12 | 0 | 41% |
| 24 | Holothuroidin 2 (H2, Echinoderm; animals; UCLL1a) | sea-cucumber, *Holothuria tubulosa* | ASHLGHHALDHLLK | Anti-Gram positive & Gram negative, Antibiofilm | 14 | 0 | 42% |
| 25 | Paracentrin 1 (SP1, Echinoderm, animals; UCLL1a) | the coelomocyte cytosol, the sea urchin, *Paracentrotus lividus* | EVASFDKSKLK | Anti-Gram positive & Gram negative, Antibiofilm | 11 | 1 | 36% |
| 26 | TetraF2W-RK (W312; Trp-rich; synthetic; BBmM; UCLL1a; XXA) | artificial, designed based on temporin-SHf | WWWLRKIW | Anti-Gram positive & Gram negative, Antifungal, Anti-MRSA, Antibiofilm | 8 | 3 | 75% |
| 27 | H4 (synthetic, a hybrid peptide containing OP-145) | artificial, Combined BMAP-27 and OP-145 | KFKKLFKKLSPVIGKEFKRIVERIKRFLR | Anti-Gram positive & Gram negative, Anti-MRSA, Antibiofilm | 29 | 10 | 41% |
| 28 | Esculentin 1-21 (Esc 1-21; synthetic; BBMm; XXA; UCLL1c) | artificial, template derived | GIFSKLAGKKIKNLLISGLKG | Anti-Gram positive & Gram negative, Antifungal, Chemotactic, Antibiofilm, Wound healing, | 21 | 6 | 42% |
| 29 | SAAP-148 (synthetic) | artificial, designed based on LL-37 | LKRVWKRVFKLLKRYWRQLKKPVR | Anti-Gram positive & Gram negative, Antibiofilm | 24 | 11 | 41% |
| 30 | Dhvar4 (synthetic) | artificial | KRLFKKLLFSLRKY | Anti-Gram positive & Gram negative, Antifungal, Antibiofilm | 9 | 3 | 66% |
| 31 | Dermaseptin-PH (Dermaseptin PH; frog, amphibians, animals; XXA; ; UCSS1c) | orange-legged leaf frog, *Pithecopus (Phyllomedusa) hypochondrialis*, South America | ALWKEVLKNAGKAALNEINNLV | Anti-Gram positive & Gram negative, Antifungal, Antibiofilm, Anticancer | 22 | 2 | 54% |
| 32 | Moronecidin-like (seahorse, fish, animals; UCLL1a) | Tiger tail seahorse, *Hippocampus comes* | FFRNLWKGAKAAFRAGHAAWRA | Anti-Gram positive & Gram negative, Antifungal, Anti-MRSA, Antibiofilm | 22 | 6 | 59% |
| 33 | MP-C (mastoporan-C, insects, arthropods, invertebrates, animals. XXA; UCLL1c) | venom, the European Hornet, *Vespa crabro* | LNLKALLAVAKKIL | Anti-Gram positive & Gram negative, Antifungal, Anti-MRSA, Hemolytic, Antibiofilm, Anticancer | 14 | 4 | 71% |
| 34 | Nigrocin-HLM (synthetic, XXA, UCLL1c) | Motif-Targeted Peptide Design | GLLSGILGAGKKIVF | Anti-Gram positive & Gram negative, Antifungal, Anti-MRSA, Antibiofilm | 15 | 2 | 53% |
| 35 | VLL-28 (archaeocins, archaea, prokaryote; UCLL1) | *Sulfolobus islandicus* | VLLVTLTRLHQRGVIYRKWRHFSGRKYR | Anti-Gram positive & Gram negative, Antifungal, Antibiofilm, Anticancer | 28 | 10 | 35% |
| 36 | Japonicin-2LF (frog, amphibians, animals; XXU; 1S=S, UCSS1a; BBMm) | skin secretion, Fujian Large-headed Frog, Limnonectes fujianensis, China, Asia | FIVPSIFLLKKAFCIALKKC | Anti-Gram positive & Gram negative, Antifungal, Anti-MRSA, Antibiofilm | 20 | 4 | 70% |
| 37 | ZmD32 (Z. mays defensin, plants; 4S=S; UCSS1a) | Corn, *Zea mays* | RTCQSQSHRFRGPCLRRSNCANVCRTEGFPGGRCRGFRRRCFCTTHC | Anti-Gram positive & Gram negative, Antifungal, Antibiofilm | 47 | 12 | 31% |
| 38 | SA-CATH (S. annularis cathelicidin; snake, reptiles, animals; Lys-rich; UCLL1a) | *Sinonatrix annularis*, China, Asia | KFFKKLKKSVKKHVKKFFKKPKVIGVSIPF | Anti-Gram positive & Gram negative, Antifungal, Anti-inflammatory, Antibiofilm | 30 | 13 | 40% |
| 39 | Hs02 (synthetic, XXA, UCLL1c) | predicted intragenic AMP | KWAVRIIRKFIKGFIS | Anti-Gram positive & Gram negative, Antifungal, Anti-inflammatory, Antibiofilm | 16 | 6 | 56% |
| 40 | Dermaseptin-PT9 (DPT9, UCLL1a; frog, amphibians, animals) | skin secretion, *Phyllomedusa tarsius,* Purchased in Peru, South America | GLWSKIKDAAKTAGKAALGFVNEMV | Anti-Gram positive & Gram negative, Antifungal, Anti-MRSA, Antibiofilm, Anticancer | 25 | 2 | 52% |
