# Supplementary material for: The antimicrobial peptide EM86 loaded to gamma-irradiated sodium alginate/polyvinyl alcohol electrospun nanofibrous dressing treated multidrug-resistant Pseudomonas aeruginosa wound infections in BALB/c mice
Source: Front Bioeng Biotechnol. 2026 Apr 7;14:1776154. doi: 10.3389/fbioe.2026.1776154 (PMC13095823; doi:10.3389/fbioe.2026.1776154)
Supplement: Supplementary file 4 [file Table5.docx]

Supplementary Table S5. MIC and MBC of freshly prepared solutions of EM02, EM85, EM86, and colistin against Gram-negative bacterial isolates determined by the broth microdilution method and Vitek-2 MIC colistin results

| **Strain** | **MIC (µg/mL)** | | | | **MBC (µg/mL)** | | | | **Vitek-2 MIC results of colistin (µg/mL)** |
| --- | --- | --- | --- | --- | --- | --- | --- | --- | --- |
|  | **EM02** | **EM85** | **EM86** | **Colistin** | **EM02** | **EM85** | **EM86** | **Colistin** |  |
| *Pseudomonas aeruginosa* SM016 | 81 ± 60 | 84 ± 54 | 4 ± 2 | 2 | 97 ± 62 | 104 ± 48 | 8 ± 2 | 2 | ≥16 |
| *Pseudomonas aeruginosa* SM012 | >128 | >128 | 32 | 2 | >128 | >128 | 64 | 3 ± 1 | ≥16 |
| *Pseudomonas aeruginosa* SM014 | >128 | >128 | 16 | 2 | >128 | >128 | 32 | 7 ± 6 | ≤0.5 |
| *Acinetobacter baumanni complex* SM008 | 4 | >128 | 16 | 4 | 16 | >128 | 16 | 4 | 4 |
| *Klebsiella pneumonia* SM022 | 16 | 16 | 16 | 8 | > 32 | 16 | 32 | 32 | - |
