# Supplementary material for: The antimicrobial peptide EM86 loaded to gamma-irradiated sodium alginate/polyvinyl alcohol electrospun nanofibrous dressing treated multidrug-resistant Pseudomonas aeruginosa wound infections in BALB/c mice
Source: Front Bioeng Biotechnol. 2026 Apr 7;14:1776154. doi: 10.3389/fbioe.2026.1776154 (PMC13095823; doi:10.3389/fbioe.2026.1776154)
Supplement: Supplementary file 5 [file Table3.docx]

Supplementary Table S3. The sequences, net charge and hydrophobicity percentage of the 21 AMPs with wound healing activity

|  | **Peptide name / Class** | **Source** | **Sequence** | **Activity** | **Length** | **Net charge** | **Hydrophobicity** |
| --- | --- | --- | --- | --- | --- | --- | --- |
| 1 | **Myxinidin** (hagfish, fish, animals; XXA; UCLL1; Derivatives: Myxinidin1; Myxinidin2; Myxinidin3; WMR) | Epidermal mucus, Myxine glutinosa L. | GIHDILKYGKPS | Anti-Gram+ & Gram-, Antifungal, Antibiofilm, Wound healing, | 12 | 2 | 25% |
| 2 | **Temporin A** (temporin-1Ta; temporin-Ta; TA; Leu-rich; XXA; UCLL1c; frog, amphibians, animals; ZZP) | European common frog, Rana temporaria | FLPLIGRVLSGIL | Anti-Gram+ & Gram-, Antiviral, Antiparasitic, Chemotactic, Wound healing, | 13 | 2 | 61% |
| 3 | **Indolicidin** (IR13; Tet083; XXA, Trp-rich, bovine cathelicidin, cattle, ruminant, mammals; animals; BBN; BBPP/BBII; Derivatives: CP-11, MBI-549, Omiganan pentahydrochloride (formerly MBI 226; MBI-226 | bovine neutrophils, Bos taurus | ILPWKWPWWPWRR | Anti-Gram+ & Gram-, Antiviral, Antifungal, Anti-HIV, Anti-MRSA, Hemolytic, Antibiofilm, Wound healing, | 13 | 4 | 53% |
| 4 | **BmKn2** (scorpions, arachnids, Chelicerata, arthropods, invertebrates, animals; XXA, UCLL1c; derivatives: Kn2-7) | venom, Buthus martensii Karsch | FIGAIARLLSKIF | Anti-Gram+ & Gram-, Antiviral, Anti-HIV, Anti-MRSA, Wound healing, | 13 | 3 | 69% |
| 5 | **K11** (synthetic, hybrid peptide, UCLL1c) | artificial, combined melittin, cecropin A1 and magainin 2 fragments | KWKSFIKKLTKKFLHSAKKF | Anti-Gram+ & Gram-, Anti-MRSA, Wound healing | 20 | 9 | 40% |
| 6 | **Epinecidin-1** (Epi-1; fish, animals; XXA; BBMm) | orange-spotted grouper, Epinephelus coioides | GFIFHIIKGLFHAGKMIHGLV | Anti-Gram+ & Gram-, Antiviral, Antifungal, Anti-MRSA, Anti-inflammatory, Wound healing, Anticancer | 21 | 3 | 57% |
| 7 | **Esculentin 1-21** (Esc 1-21; synthetic; BBMm; XXA; UCLL1c) | artificial, template derived | GIFSKLAGKKIKNLLISGLKG | Anti-Gram+ & Gram-, Antifungal, Chemotactic, Antibiofilm, Wound healing | 21 | 6 | 42% |
| 8 | **Magainin 2** (Magainin II, PGS; Hebrew word for "shield"; UCLL1; frog, amphibians, animals. Derivatives: MSI-78, = pexiganan, pexiganan acetate, or cytolex, loxilex; clinical trials; MSI-594; MSI-99. | skin; Stomach, African clawed frog, Xenopus laevis, Africa | GIGKFLHSAKKFGKAFVGEIMNS | Anti-Gram+ & Gram-, Antiviral, Antifungal, Antiparasitic, Insecticidal, Spermicidal, Antimalarial, Wound healing, Anticancer | 23 | 3 | 43% |
| 9 | **TP3** (Tilapia piscidin 3; His-rich; fish, animals; inactive: TP1, TP2, and TP5) | Nile Tilapia, Oreochromis niloticus | FIHHIIGGLFSVGKHIHSLIHGH | Anti-Gram+ & Gram-, Anti-MRSA, Hemolytic, Wound healing | 23 | 7 | 43% |
| 10 | **TP4** (Tilapia piscidin 4; Oreoch-2; MSP-4; fish, animals; BBL) | Gills, Nile Tilapia, Oreochromis niloticus | FIHHIIGGLFSAGKAIHRLIRRRRR | Anti-Gram+ & Gram-, Antifungal, Hemolytic, Wound healing, Anticancer | 25 | 7 | 44% |
| 11 | **Human neutrophil peptide-1** (HNP-1, HNP1, alpha Defensin, UCSS1a; lectin; primates, mammals, animals; XXX; ZZHh, BBS; BBL; BBW; 3S=S) | neutrophils; natural killer cells, monocytes; saliva; Homo sapiens | ACYCRIPACIAGERRYGTCIYQGRLWAFCC | Anti-Gram+ & Gram-, Antiviral, Antifungal, Antiparasitic, Anti-HIV, Chemotactic, Anti-MRSA, Anti-toxin, Enzyme inhibitor, Wound healing, Anticancer | 30 | 3 | 53% |
| 12 | **Human defensin 5** (HD-5, HD5, DEFA5; intestinal, alpha Defensin; UCSS1a; primates, mammals, animals; BBBH2O; 3S=S; BBL) | Paneth cells, intestine, Homo sapiens | ATCYCRTGRCATRESLSGVCEISGRLYRLCCR | Anti-Gram+ & Gram-, Antiviral, Antifungal, Anti-toxin, Wound healing | 32 | 4 | 40% |
| 13 | **Brevinin-2Ta** (frog, amphibians, animals; UCSS1a; 1S=S; XXU) | Pelophylax kl. esculentus, Europe | GILDTLKNLAKTAGKGILKSLVNTASCKLSGQC | Anti-Gram+ & Gram-, Antifungal, Wound healing | 33 | 4 | 42% |
| 14 | **Nisin A** (NisaplinTM, ChrisinTM, food additive E234; lantibiotic, type 1, class 1 bacteriocin, Gram-positive bacteria, prokaryotes; XXT5; XXW3; UCSS1b; ZZS; BBW; BBMm; JJsn; Variants: nisin AP) | Streptococcus lactis, reclassified as Lactococcus lactis | ITSISLCTPGCKTGALMGCNMKTATCHCSIHVSK | Anti-Gram+, Spermicidal, Antibiofilm, Wound healing, Anticancer | 34 | 3 | 44% |
| 15 | **LL-37** [LL37; FALL-39; cathelicidin; UCLL1; human; chimpanzee; primates, mammals, animals; XXX; XXY; XXZ; BBBh2o, BBBm; BBMm, BBPP, BBN, BBL, BBrsg, JJsn; Derivatives: many) | neutrophils, monocytes; mast cells; lymphocytes, Mesenchymal Stem Cells; islets; skin, sweat; airway surface liquid, saliva; Homo sapiens; Also Pan troglodytes | LLGDFFRKSKEKIGKEFKRIVQRIKDFLRNLVPRTES | Anti-Gram+ & Gram-, Antiviral, Antifungal, Antiparasitic, Spermicidal, Anti-HIV, Chemotactic, Anti-MRSA, Enzyme inhibitor, Hemolytic, Antibiofilm, Wound healing, Anticancer | 37 | 6 | 35% |
| 16 | **PR-39** (PR39, a proline-arginine-rich peptide with 39 residues, XXA, cathelicidin, pigs, mammals; animals; Pro-rich; Arg-rich; BBN) | Pig neutrophils; , Sus scrofa | RRRPRPPYLPRPRPPPFFPPRLPPRIPPGFPPRFPPRFP | Anti-Gram+ & Gram-, Wound healing, Anticancer | 39 | 11 | 20% |
| 17 | **Lucifensin** (Lucilia sericata Meigen defensin, UCSS1a; insects, arthropods, invertebrates, animals; 3S=S; BBW) | gut, salivary glands, fat body, haemolymph; blowfly; maggots; Lucilia sericata | ATCDLLSGTGVKHSACAAHCLLRGNRGGYCNGRAICVCRN | Anti-Gram+, Wound healing | 40 | 4 | 45% |
| 18 | **Human beta defensin 2** (hBD-2; hBD2; UCSS1a; human, primates, mammals, animals; ZZHh; 3S=S; JJsn) | skin, lung, trachea epithelia, and uterus, oral (saliva); Homo sapiens | GIGDPVTCLKSGAICHPVFCPRRYKQIGTCGLPGTKCCKKP | Anti-Gram+ & Gram-, Antiviral, Antifungal, Anti-HIV, Chemotactic, Anti-toxin, Channel inhibitors, Wound healing, | 41 | 7 | 36% |
| 19 | **Myticin C** (Myt C; mollusca/molluscs/mollusks, invertebrates, animals; 4S=S; Variants: reduced form XXR) | the mediterranean mussel, Mytilus galloprovincialis | QEAQSVACTSYYCSKFCGSAGCSLYGCYLLHPGKICYCLHCSR | Anti-Gram+ & Gram-, Antiviral, Chemotactic, Wound healing, | 43 | 2 | 41% |
| 20 | **Coprisin** (defensin-like peptide; UCSS1a; 3S=S, insects, arthropods, invertebrates, animals; Derivatives: CopA3) | Dung Beetle, Copris tripartitus. | VTCDVLSFEAKGIAVNHSACALHCIALRKKGGSCQNGVCVCRN | Antifungal, Anti-inflammatory, Antibiofilm, Wound healing, | 43 | 3 | 51% |
| 21 | **Coprisin** (defensin-like peptide; UCSS1a; 3S=S, insects, arthropods, invertebrates, animals; Derivatives: CopA3) | skin, tonsils, oral/saliva, Homo sapiens | GIINTLQKYYCRVRGGRCAVLSCLPKEEQIGKCSTRGRKCCRRKK | Anti-Gram+ & Gram-, Antiviral, Antifungal, Anti-HIV, Chemotactic, Anti-MRSA, Anti-toxin, Antibiofilm, Wound healing, Anticancer | 45 | 11 | 33% |
