# Supplementary material for: The antimicrobial peptide EM86 loaded to gamma-irradiated sodium alginate/polyvinyl alcohol electrospun nanofibrous dressing treated multidrug-resistant Pseudomonas aeruginosa wound infections in BALB/c mice
Source: Front Bioeng Biotechnol. 2026 Apr 7;14:1776154. doi: 10.3389/fbioe.2026.1776154 (PMC13095823; doi:10.3389/fbioe.2026.1776154)
Supplement: Supplementary file 6 [file Table4.docx]

Supplementary Table S4. Sequences of the generated colistin and polymyxin analogs, predicted membrane activity, length, net charge, hydrophobicity percentage, GRAVY, Boman Index, Wimley-White whole residue hydrophobicity, core segment hydrophobicity, molecular weight, number of hydrophobic residues on the same surface, and 3D structure

|  |  |  | **Predicted membrane activity** | | | | **Length** | **Net charge** | **Hydrophobicity** | **GRAVY** | **Boman Index** | **The Wimley-White whole-residue hydrophobicity of the peptide** | **CSH according to Liu Deber** | **Molecular weight** | **Number of hydrophobic residues on the same surface** | **3D structure** |
| --- | --- | --- | --- | --- | --- | --- | --- | --- | --- | --- | --- | --- | --- | --- | --- | --- |
|  |  | **Possible predicted sequences for modified colistin (KTKKKLLKKT)** | **SVM** | **RF** | **ANN** | **DA** |  |  |  |  |  |  | **1.84** |  |  |  |
| 1 | EM01 | WWWKTKKKLLKKT | 1 | 0.9145 | AMP | 0.997 | 13 | +6 | 38% | -1.5 | 1.66 kcal/mol | -0.45 kcal/mol | 3.14 | 1774.204 | 0 | not form helix |
| 2 | **EM02** | **WWWKAKKKAAKKG** | **1** | **0.94** | **AMP** | **0.992** | **13** | **+6** | **46%** | **-1.623** | **1.53 kcal/mol** | **0.91 kcal/mol** | **1.69** | **1615.963** | **4** | **form α helix** |
| 3 | EM03 | WWWKAKKKLLKKT | 1 | 0.944 | AMP | 0.999 | 13 | +6 | 46% | -1.33 | 1.32 kcal/mol | -0.42 kcal/mol | 3.32 | 1744.178 | 4 | form α helix |
| 4 | EM04 | WWWKAKKKALKKT | 1 | 0.9235 | AMP | 0.998 | 13 | +6 | 46% | -1.49 | 1.56 kcal/mol | 0.31 kcal/mol | 2.66 | 1702.097 | 4 | form α helix |
| 5 | EM05 | WWWKAKKKAAKKT | 1 | 0.9025 | AMP | 0.998 | 13 | +6 | 46% | -1.64 | 1.8 kcal/mol | 1.04 kcal/mol | 2.01 | 1660.016 | 4 | form α helix |
| 6 | EM06 | WWWKIKKKLLKKT | 1 | 0.9415 | AMP | 1 | 13 | +6 | 46% | -1.13 | 1.08 kcal/mol | -0.9 kcal/mol | 3.9 | 1786.259 | 4 | form α helix |
| 7 | EM07 | WWWKIKKKILKKT | 1 | 0.928 | AMP | 1 | 13 | +6 | 46% | -1.076 | 1.08 kcal/mol | -0.65 kcal/mol | 3.87 | 1786.259 | 4 | form α helix |
| 8 | EM08 | WWWKIKKKIIKKT | 1 | 0.9245 | AMP | 1 | 13 | +6 | 46% | -1.023 | 1.08 kcal/mol | -0.4 kcal/mol | 3.82 | 1786.259 | 4 | form α helix |
| 9 | EM09 | WWWKIKKKIIKKG | 1 | 0.9845 | AMP | 0.998 | 13 | +6 | 46% | -1 | 0.81 kcal/mol | -0.53 kcal/mol | 3.5 | 1742.206 | 4 | form α helix |
| 10 | EM10 | WWWKAKKKAIKKT | 1 | 0.936 | AMP | 0.998 | 13 | +6 | 46% | -1.43 | 1.56 kcal/mol | 0.56 kcal/mol | 2.6 | 1702.097 | 4 | form α helix |
| 11 | EM11 | WWWKAKKKALKKT | 1 | 0.9235 | AMP | 0.998 | 13 | +6 | 46% | -1.49 | 1.56 kcal/mol | 0.31 kcal/mol | 2.66 | 1702.097 | 4 | form α helix |
| 12 | EM12 | WWWKAKKKAIKKG | 1 | 0.975 | AMP | 0.993 | 13 | +6 | 46% | -1.415 | 1.29 kcal/mol | 0.43 kcal/mol | 2.29 | 1658.044 | 4 | form α helix |
| 13 | EM13 | WWWKAKKKALKKG | 1 | 0.9665 | AMP | 0.993 | 13 | +6 | 46% | -1.46 | 1.29 kcal/mol | 0.18 kcal/mol | 2.34 | 1658.044 | 4 | form α helix |
| 14 | EM14 | WWWKAKKKIAKKT | 1 | 0.936 | AMP | 0.998 | 13 | +6 | 46% | -1.438 | 1.56 kcal/mol | 0.56 kcal/mol | 2.6 | 1702.097 | 4 | form α helix |
| 15 | EM15 | WWWKAKKKLAKKT | 1 | 0.9235 | AMP | 0.998 | 13 | +6 | 46% | -1.49 | 1.56 kcal/mol | 0.31 kcal/mol | 2.66 | 1702.097 | 4 | form α helix |
| 16 | EM16 | WWWKAKKKLIKKT | 1 | 0.946 | AMP | 0.999 | 13 | +6 | 46% | -1.284 | 1.32 kcal/mol | '-0.17 kcal/mol | 3.2 | 1744.178 | 4 | form α helix |
| 17 | EM17 | WWWKAKKKILKKT | 1 | 0.946 | AMP | 0.999 | 13 | +6 | 46% | -1.284 | 1.32 kcal/mol | -0.17 kcal/mol | 3.2 | 1744.178 | 4 | form α helix |
| 18 | EM18 | WWWKAKKKIAKKG | 1 | 0.975 | AMP | 0.993 | 13 | +6 | 46% | -1.415 | 1.29 kcal/mol | 0.43 kcal/mol | 2.29 | 1658.044 | 4 | form α helix |
| 19 | EM19 | WWWKAKKKLAKKG | 1 | 0.9665 | AMP | 0.993 | 13 | +6 | 46% | -1.469 | 1.29 kcal/mol | 0.18 kcal/mol | 2.34 | 1658.044 | 4 | form α helix |
| 20 | EM20 | WWWKAKKKLIKKG | 1 | 0.982 | AMP | 0.995 | 13 | +6 | 46% | -1.26 | 1.05 kcal/mol | -0.3 kcal/mol | 2.95 | 1700.125 | 4 | form α helix |
| 21 | EM21 | WWWKAKKKILKKG | 1 | 0.982 | AMP | 0.995 | 13 | +6 | 46% | -1.26 | 1.05 kcal/mol | -0.3 kcal/mol | 2.95 | 1700.125 | 4 | form α helix |
|  |  | **Possible predicted sequences for modified Polymyxin (KTKKKFLKKT)** |  |  |  |  |  |  |  |  |  |  | **1.9** |  |  |  |
| 22 | EM22 | WWWKTKKKFGKKG | 1 | 0.8555 | AMP | 0.999 | 13 | 6 | 30% | '-1.9 | 1.84 kcal/mol | -0.58 kcal/m | 2.17 | 1708.06 | 0 | **Not form α helix** |
| 23 | EM23 | WWWKTKKKFAKKT | 1 | 0.84 | AMP | 0.998 | 13 | 6 | 38% | -1.76 | 2.05 kcal/mol | -0.29 kcal/mol | 2.52 | 1766.14 | 0 | **Not form α helix** |
| 24 | EM24 | WWWKTKKKFIKKT | 1 | 0.9045 | AMP | 0.998 | 13 | 6 | 38% | -1.55 | 1.81 kcal/mol | -0.77 kcal/mol | 3.12 | 1808.221 | 0 | **Not form α helix** |
| 25 | EM25 | WWWKTKKKFAKKG | 1 | 0.8685 | AMP | 0.999 | 13 | 6 | 38% | -1.738 | 1.78 kcal/mol | -0.42 kcal/mol | 2.2 | 1722.087 | 0 | **Not form α helix** |
| 26 | EM26 | WWWKTKKKFIKKG | 1 | 0.937 | AMP | 0.999 | 13 | 6 | 38% | -1.53 | 1.54 kcal/mol | -0.9 kcal/mol | 2.8 | 1764.168 | 0 | **Not form α helix** |
| 27 | EM27 | WWWKTKKKAFKKT | 1 | 0.84 | AMP | 0.998 | 13 | 6 | 38% | -1.76 | 2.05 kcal/mol | -0.29 kcal/mol | 2.52 | 1766.14 | 0 | **Not form α helix** |
| 28 | EM28 | WWWKTKKKIFKKT | 1 | 0.9045 | AMP | 0.998 | 13 | 6 | 38% | -1.55 | 1.81 kcal/mol | -0.77 kcal/mol | 2.43 | 1808.221 | 0 | **Not form α helix** |
| 29 | EM29 | WWWKTKKKAFKKG | 1 | 0.8685 | AMP | 0.999 | 13 | 6 | 38% | -1.738 | 1.78 kcal/mol | -0.42 kcal/mol | 2.2 | 1722.087 | 0 | **Not form α helix** |
| 30 | EM30 | WWWKTKKKIFKKG | 1 | 0.937 | AMP | 0.999 | 13 | 6 | 38% | -1.53 | 1.54 kcal/mol | -0.9 kcal/mol | 2.095 | 1764.168 | 0 | **Not form α helix** |
| 31 | EM31 | WWWKGKKKFLKKT | 1 | 0.9355 | AMP | 0.999 | 13 | 6 | 38% | -1.58 | 1.54 kcal/mol | -1.15 kcal/mol | 2.85 | 1764.168 | 0 | **Not form α helix** |
| 32 | EM32 | WWWKGKKKFLKKG | 1 | 0.975 | AMP | 0.997 | 13 | 6 | 38% | -1.56 | 1.27 kcal/mol | -1.28 kcal/mol | 2.55 | 1720.115 | 0 | **Not form α helix** |
| 33 | EM33 | WWWKGKKKFAKKT | 1 | 0.8635 | AMP | 0.999 | 13 | 6 | 38% | -1.738 | 1.78 kcal/mol | -0.42 kcal/mol | 2.2 | 1722.087 | 0 | **Not form α helix** |
| 34 | EM34 | WWWKGKKKFIKKT | 1 | 0.933 | AMP | 0.999 | 13 | 6 | 38% | -1.53 | 1.54 kcal/mol | -0.9 kcal/mol | 2.8 | 1764.168 | 0 | **Not form α helix** |
| 35 | EM35 | WWWKGKKKFAKKG | 1 | 0.9085 | AMP | 0.997 | 13 | 6 | 38% | -1.715 | 1.51 kcal/mol | -0.55 kcal/mol | **1.88** | 1678.034 | 0 | **Not form α helix** |
| 36 | EM36 | WWWKGKKKFIKKG | 1 | 0.9755 | AMP | 0.998 | 13 | 6 | 38% | -1.507 | 1.27 kcal/mol | -1.03 kcal/mol | 2.49 | 1720.115 | 0 | **Not form α helix** |
| 37 | EM37 | WWWKGKKKLFKKT | 1 | 0.9355 | AMP | 0.999 | 13 | 6 | 38% | -1.58 | 1.54 kcal/mol | -1.15 kcal/mol | 2.85 | 1764.168 | 0 | **Not form α helix** |
| 38 | EM38 | WWWKGKKKLFKKG | 1 | 0.975 | AMP | 0.997 | 13 | 6 | 38% | -1.56 | 1.27 kcal/mol | -1.28 kcal/mol | 2.54 | 1720.115 | 0 | **Not form α helix** |
| 39 | EM39 | WWWKGKKKAFKKT | 1 | 0.8635 | AMP | 0.999 | 13 | 6 | 38% | -1.738 | 1.78 kcal/mol | -0.42 kcal/mol | 2.2 | 1722.087 | 0 | **Not form α helix** |
| 40 | EM40 | WWWKGKKKAFKKG | 1 | 0.9085 | AMP | 0.997 | 13 | 6 | 38% | -1.715 | 1.51 kcal/mol | -0.55 kcal/mol | **1.88** | 1678.034 | 0 | **Not form α helix** |
| 41 | EM41 | WWWKGKKKIFKKT | 1 | 0.933 | AMP | 0.999 | 13 | 6 | 38% | -1.53 | 1.54 kcal/mol | -0.9 kcal/mol | 2.8 | 1764.168 | 0 | **Not form α helix** |
| 42 | EM42 | WWWKGKKKIFKKG | 1 | 0.9755 | AMP | 0.998 | 13 | 6 | 38% | -1.5 | 1.27 kcal/mol | -1.03 kcal/mol | 2.49 | 1720.115 | 0 | **Not form α helix** |
| 43 | EM43 | W**WWKFKKKTLKKT** | 1 | 0.91 | AMP | 0.997 | 13 | 6 | 38% | -1.607 | 1.81 kcal/mol | -1.02 kcal/mol | 3.17 | 1808.221 | 3 | form α helix |
| 44 | EM44 | W**WWKFKKKTIKKT** | 1 | 0.9035 | AMP | 0.997 | 13 | 6 | 38% | -1.55 | 1.81 kcal/mol | -0.77 kcal/mol | 3.12 | 1808.221 | 3 | form α helix |
| 45 | EM45 | W**WWKFKKKTAKKT** | 1 | 0.836 | AMP | 0.997 | 13 | 6 | 38% | -1.76 | 2.05 kcal/mol | -0.29 kcal/mol | 2.52 | 1766.14 | 3 | form α helix |
| 46 | EM46 | W**WWKFKKKTLKKG** | 1 | 0.983 | AMP | 0.999 | 13 | 6 | 38% | -1.58 | 1.54 kcal/mol | -1.15 kcal/mol | 2.85 | 1764.168 | 3 | form α helix |
| 47 | EM47 | W**WWKFKKKTIKKG** | 1 | 0.9835 | AMP | 0.999 | 13 | 6 | 38% | -1.53 | 1.54 kcal/mol | -0.9 kcal/mol | 2.8 | 1764.168 | 3 | form α helix |
| 48 | EM48 | W**WWKFKKKTAKKG** | 1 | 0.9005 | AMP | 0.999 | 13 | 6 | 38% | -1.738 | 1.78 kcal/mol | -0.42 kcal/mol | 2.27 | 1722.087 | 3 | form α helix |
| 49 | EM49 | W**WWKFKKKLTKKT** | 1 | 0.9065 | AMP | 0.997 | 13 | 6 | 38% | -1.607 | 1.81 kcal/mol | -1.02 kcal/mol | 3.1 | 1808.221 | 4 | form α helix |
| 50 | EM50 | W**WWKFKKKITKKT** | 1 | 0.9 | AMP | 0.997 | 13 | 6 | 38% | -1.5 | 1.81 kcal/mol | -0.77 kcal/mol | 3.12 | 1808.221 | 4 | form α helix |
| 51 | EM51 | W**WWKFKKKATKKT** | 1 | 0.833 | AMP | 0.997 | 13 | 6 | 38% | -1.76 | 2.05 kcal/mol | -0.29 kcal/mol | 2.5 | 1766.14 | 4 | form α helix |
| 52 | EM52 | W**WWKFKKKLTKKG** | 1 | 0.983 | AMP | 0.999 | 13 | 6 | 38% | -1.58 | 1.54 kcal/mol | -1.15 kcal/mol | 2.85 | 1764.168 | 4 | form α helix |
| 53 | EM53 | W**WWKFKKKITKKG** | 1 | 0.9005 | AMP | 0.999 | 13 | 6 | 38% | -1.53 | 1.54 kcal/mol | -0.9 kcal/mol | 2.8 | 1764.168 | 4 | form α helix |
| 54 | EM54 | W**WWKFKKKATKKG** | 1 | 0.9005 | AMP | 0.999 | 13 | 6 | 38% | -1.73 | 1.78 kcal/mol | -0.42 kcal/mol | 2.2 | 1722.087 | 4 | form α helix |
| 55 | EM55 | W**WWKFKKKGLKKT** | 1 | 0.9355 | AMP | 0.999 | 13 | 6 | 38% | -1.584 | 1.54 kcal/mol | -1.15 kcal/mol | 2.85 | 1764.168 | 3 | form α helix |
| 56 | EM56 | W**WWKFKKKGIKKT** | 1 | 0.933 | AMP | 0.999 | 13 | 6 | 38% | -1.53 | 1.54 kcal/mol | -0.9 kcal/mol | 2.8 | 1764.168 | 3 | form α helix |
| 57 | EM57 | W**WWKFKKKGAKKT** | 1 | 0.8635 | AMP | 0.999 | 13 | 6 | 38% | -1.73 | 1.78 kcal/mol | -0.42 kcal/mol | 2.2 | 1722.087 | 3 | form α helix |
| 58 | EM58 | W**WWKFKKKGLKKG** | 1 | 0.975 | AMP | 0.997 | 13 | 6 | 38% | -1.56 | 1.27 kcal/mol | -1.28 kcal/mol | 2.54 | 1720.115 | 3 | form α helix |
| 59 | EM59 | W**WWKFKKKGIKKG** | 1 | 0.9755 | AMP | 0.998 | 13 | 6 | 38% | -1.5 | 1.27 kcal/mol | -1.03 kcal/mol | 2.49 | 1720.115 | 3 | form α helix |
| 60 | **EM60** | **WWWKFKKKGAKKG** | **1** | **0.909** | **AMP** | **0.997** | 13 | 6 | **38%** | **-1.715** | **1.51 kcal/mol** | **-0.55 kcal/mol** | **1.88** | **1678.034** | **3** | **form α helix** |
| 61 | EM61 | WWWKGKKKFGKKG | 1 | 0.9045 | AMP | 0.997 | 13 | 6 | 30% | -1.88 | 1.57 kcal/mol | -0.71 kcal/mol | **1.38** | 1664.007 | 0 | **Not form helix** |
| 62 | EM62 | WWWKGKKKFGKKT | 1 | 0.853 | AMP | 0.999 | 13 | 6 | 30% | -1.9 | 1.84 kcal/mol | -0.58 kcal/mol | **1.7** | 1708.06 | 0 | **Not form helix** |
| 63 | EM63 | WWWKGKKKGFKKT | 1 | 0.853 | AMP | 0.999 | 13 | 6 | 30% | -1.90 | 1.84 kcal/mol | -0.58 kcal/mol | **1.7** | 1708.06 | 0 | **Not form helix** |
| 64 | EM64 | WWWKGKKKGFKKG | 1 | 0.9045 | AMP | 0.997 | 13 | 6 | 30% | -1.88 | 1.57 kcal/mol | -0.71 kcal/mol | **1.38** | 1664.007 | 0 | **Not form helix** |
| 65 | EM65 | WWWKGKKKGLKKG | 1 | 0.952 | AMP | 0.994 | 13 | 6 | 30% | -1.8 | 1.42 kcal/mol | -0.14 kcal/mol | **1.35** | 1629.99 | 0 | **Not form helix** |
| 66 | EM66 | WWWKGKKKGIKKG | 1 | 0.957 | AMP | 0.994 | 13 | 6 | 30% | -1.75 | 1.42 kcal/mol | 0.11 kcal/mol | **1.3** | 1629.99 | 0 | **Not form helix** |
| 67 | EM67 | WWWKGKKKGAKKG | 1 | 0.856 | AMP | 0.993 | 13 | 6 | 30% | -1.96 | 1.66 kcal/mol | 0.59 kcal/mol | **0.69** | 1587.909 | 0 | **Not form helix** |
| 68 | EM68 | WWWKGKKKGLKKT | 1 | 0.898 | AMP | 0.999 | 13 | 6 | 30% | -1.83 | 1.69 kcal/mol | -0.01 kcal/mol | **1.67** | 1674.043 | 0 | **Not form helix** |
| 69 | EM69 | WWWKGKKKGIKKT | 1 | 0.9005 | AMP | 0.999 | 13 | 6 | 30% | -1.77 | 1.69 kcal/mol | 0.24 kcal/mol | **1.62** | 1674.043 | 0 | **Not form helix** |
| 70 | EM70 | WWWKGKKKGAKKT | 1 | 0.8245 | AMP | 0.998 | 13 | 6 | 30% | '-1.98 | 1.93 kcal/mol | 0.72 kcal/mol | **1.05** | 1631.962 | 0 | **Not form helix** |
| 71 | EM71 | WWWKGKKKLGKKG | 1 | 0.952 | AMP | 0.994 | 13 | 6 | 30% | -1.8 | 1.42 kcal/mol | -0.14 kcal/mol | **1.35** | 1629.99 | 0 | **Not form helix** |
| 72 | EM72 | WWWKGKKKLGKKT | 1 | 0.898 | AMP | 0.999 | 13 | 6 | 30% | -1.83 | 1.69 kcal/mol | -0.01 kcal/mol | **1.67** | 1674.043 | 0 | **Not form helix** |
| 73 | EM73 | WWWKGKKKIGKKG | 1 | 0.957 | AMP | 0.994 | 13 | 6 | 30% | -1.75 | 1.42 kcal/mol | 0.11 kcal/mol | **1.3** | 1629.99 | 0 | **Not form helix** |
| 74 | EM74 | WWWKGKKKIGKKT | 1 | 0.9005 | AMP | 0.999 | 13 | 6 | 30% | -1.77 | 1.69 kcal/mol | 0.24 kcal/mol | **1.62** | 1674.043 | 0 | **Not form helix** |
| 75 | EM75 | WWWKGKKKAGKKG | 1 | 0.856 | AMP | 0.993 | 13 | 6 | 30% | -1.96 | 1.66 kcal/mol | 0.59 kcal/mol | **0.69** | 1587.909 | 0 | **Not form helix** |
| 76 | EM76 | WWWKGKKKAGKKT | 1 | 0.8245 | AMP | 0.998 | 13 | 6 | 30% | -1.98 | 1.93 kcal/mol | 0.72 kcal/mol | **1.05** | 1631.962 | 0 | **Not form helix** |
| 77 | EM77 | WWWKAKKKFLKKT | 1 | 0.9395 | AMP | 0.999 | 13 | 6 | 46% | -1.415 | 1.47 kcal/mol | -0.99 kcal/mol | 3.35 | 1778.195 | 4 | form α helix |
| 78 | EM78 | WWWKAKKKFIKKT | 1 | 0.942 | AMP | 0.999 | 13 | 6 | 46% | -1.36 | 1.47 kcal/mol | -0.74 kcal/mol | 3.3 | 1778.195 | 4 . | form α helix |
| 79 | EM79 | WWWKAKKKFAKKT | 1 | 0.8785 | AMP | 0.999 | 13 | 6 | 46% | -1.56 | 1.71 kcal/mol | -0.26 kcal/mol | 2.7 | 1736.114 | 4 | form α helix |
| 80 | EM80 | WWWKAKKKFGKKT | 1 | 0.8635 | AMP | 0.999 | 13 | 6 | 38% | -1.738 | 1.78 kcal/mol | -0.42 kcal/mol | 2.2 | 1722.087 | 4 | form α helix |
| 81 | EM81 | WWWKAKKKFLKKG | 1 | 0.976 | AMP | 0.997 | 13 | 6 | 46% | -1.39 | 1.2 kcal/mol | -1.12 kcal/mol | 3.03 | 1734.142 | 4 | form α helix |
| 82 | EM82 | WWWKAKKKFIKKG | 1 | 0.978 | AMP | 0.997 | 13 | 6 | 46% | -1.33 | 1.2 kcal/mol | -0.87 kcal/mol | 2.9 | 1734.142 | 4 | form α helix |
| 83 | EM83 | WWWKAKKKFAKKG | 1 | 0.9245 | AMP | 0.996 | 13 | 6 | 46% | -1.546 | 1.44 kcal/mol | -0.39 kcal/mol | 2.38 | 1692.061 | 4 | form α helix |
| 84 | **EM84** | **WWWKAKKKFGKKG** | **1** | **0.909** | **AMP** | **0.997** | **13** | **6** | **38%** | **-1.715** | **1.51 kcal/mol** | **-0.55 kcal/mol** | **1.88** | **1678.034** | **4** | **form α helix** |
| 85 | **EM2 modified (EM86)** | **WWWKAAKKAKKKG** | **1** | **0.935** | **AMP** | **0.992** | **13** | **6** | **46%** | **-1.623** | **1.53 kcal/mol** | **0.91 kcal/mol** | **1.69** | **1615.963** | **5** | **form α helix** |
| 86 | **EM60 modified** | **WWWKFKKKAGKKG** | **1** | **0.909** | **AMP** | **0.997** | **13** | **6** | **38%** | **-1.71** | **1.51 kcal/mol** | **-0.55 kcal/mol** | **1.88** | **1678.034** | **4** | **form α helix** |

GRAVY is the grand average hydrophobicity as obtained from the APD3, it is the sum of Kyle-Doolittle hydrophobicity scale values of amino acids in the peptides’ side chain divided by their number, and it used to determine peptide solubility, a negative GRAVY value indicates peptide hydrophilicity and solubility. Boman index indicates the protein binding potential, it is the sum of the free energies of the respective side chains for transfer from cyclohexane to water, taken from Radzeka and Wolfenden, and divided by the total number of the residues of the antimicrobial peptide. Wimley-White whole residue hydrophobicity, indicates the contribution of both the peptides’ side chain and backbone to peptide hydrophobicity, a more hydrophobic peptide tends to have a more negative value, while a more hydrophilic peptide tends to have a more positive value. All are obtained from APD3. CSH is calculated by summing the hydrophobicity values of its amino acid residues, according to Liu and Deber, divided by their number excluding arginine and lysine.
