# Supplementary material for: The antimicrobial peptide EM86 loaded to gamma-irradiated sodium alginate/polyvinyl alcohol electrospun nanofibrous dressing treated multidrug-resistant Pseudomonas aeruginosa wound infections in BALB/c mice
Source: Front Bioeng Biotechnol. 2026 Apr 7;14:1776154. doi: 10.3389/fbioe.2026.1776154 (PMC13095823; doi:10.3389/fbioe.2026.1776154)
Supplement: Supplementary file 7 [file Table6.docx]

Supplementary Table S6. Electrospinning response and fiber productivity scoring of coded runs

| Coded run | Ratio SA: PVA | Q (mm/min) | V (KV) | X (cm) | Response and fiber productivity score (0-3) |
| --- | --- | --- | --- | --- | --- |
| A | 1:6.5 | 0.016 | 20 | 12.5 | 0 |
| B |  | 0.016 | 25 | 12.5 | 0 |
| C |  | 0.016 | 29 | 12.5 | 0 |
| D |  | 0.016 | 30 | 12.5 | 0 |
| E | 1:10 | 0.025 | 29 | 12.5 | 1 |
| F |  | 0.05 | 29 | 12.5 | 1 |
| G |  | 0.05 | 30 | 12.5 | 1 |
| H | 1:12.3 | 0.016 | 29 | 12.5 | 0 |
| I |  | 0.016 | 28 | 12.5 | 1 |
| J |  | 0.016 | 27 | 12.5 | 1 |
| K |  | 0.016 | 26 | 12.5 | 1 |
| L |  | 0.016 | 20 | 12.5 | 1 |
| M |  | 0.016 | 18 | 12.5 | 1 |
| N |  | 0.016 | 23 | 12.5 | 1 |
| O |  | 0.016 | 25 | 12.5 | 2 |
| P |  | 0.016 | 22 | 12.5 | 0 |
| Q |  | 0.016 | 24 | 12.5 | 1 |
| R |  | 0.018 | 29 | 12.5 | 1 |
| S |  | 0.018 | 26 | 12.5 | 1 |
| T |  | 0.018 | 22 | 12.5 | 0 |
| U |  | 0.018 | 24 | 12.5 | 1 |
| V |  | 0.018 | 23 | 12.5 | 1 |
| W |  | 0.018 | 27 | 12.5 | 0 |
| X |  | 0.018 | 25 | 12.5 | 2 |
| Y |  | 0.02 | 29 | 12.5 | 1 |
| Z |  | 0.025 | 30 | 12.5 | 1 |
| AA |  | 0.035 | 30 | 12.5 | 2 |
| AB |  | 0.035 | 29 | 12.5 | 1 |
| AC |  | 0.035 | 28 | 12.5 | 1 |
| AD |  | 0.035 | 27 | 12.5 | 1 |
| AE |  | 0.035 | 26 | 12.5 | 1 |
| AF |  | 0.045 | 30 | 12.5 | 3 |
| AG |  | 0.045 | 28 | 12.5 | 2 |
| AH |  | 0.045 | 29 | 12.5 | 2 |
| AI |  | 0.055 | 30 | 12.5 | 2 |
| AJ | 1:20 | 0.015 | 18 | 12.5 | 0 |
| AK |  | 0.02 | 30 | 12.5 | 0 |
| AL |  | 0.02 | 29 | 12.5 | 0 |
| AM |  | 0.035 | 30 | 12.5 | 3 |
| AN |  | 0.035 | 29 | 12.5 | 1 |
| AO |  | 0.045 | 30 | 12.5 | 2 |
| AP |  | 0.045 | 28 | 12.5 | 0 |
| AR |  | 0.055 | 30 | 12.5 | 2 |
| AS |  | 0.055 | 25 | 12.5 | 1 |
| AT |  | 0.055 | 26 | 12.5 | 2 |
| AU |  | 0.055 | 27 | 12.5 | 2 |
| AV |  | 0.055 | 28 | 12.5 | 2 |
| AW |  | 0.055 | 29 | 12.5 | 2 |
| AX |  | 0.065 | 30 | 12.5 | 2 |
| AY |  | 0.065 | 25 | 12.5 | 1 |
| AZ |  | 0.065 | 26 | 12.5 | 1 |
| AAA |  | 0.065 | 27 | 12.5 | 1 |
| AAB |  | 0.065 | 28 | 12.5 | 1 |
| AAC |  | 0.065 | 29 | 12.5 | 1 |
| AAD |  | 0.075 | 25 | 12.5 | 0 |
| AAE |  | 0.1 | 30 | 12.5 | 0 |

Coded runs are given scores (0-3) based on electrospinning response, and the scoring system was as follows fiber productivity, electrospraying and no fiber production (score 0), low- density fibers, and dried jets (score 1), moderate density fibers (score 2), high density fibers (score 3).
