# Supplementary material for: The antimicrobial peptide EM86 loaded to gamma-irradiated sodium alginate/polyvinyl alcohol electrospun nanofibrous dressing treated multidrug-resistant Pseudomonas aeruginosa wound infections in BALB/c mice
Source: Front Bioeng Biotechnol. 2026 Apr 7;14:1776154. doi: 10.3389/fbioe.2026.1776154 (PMC13095823; doi:10.3389/fbioe.2026.1776154)

Supplementary Figure S1: Multiple sequence alignment of the 40 AMPs with anti-Gram-negative and antibiofilm activity using Clustal omega

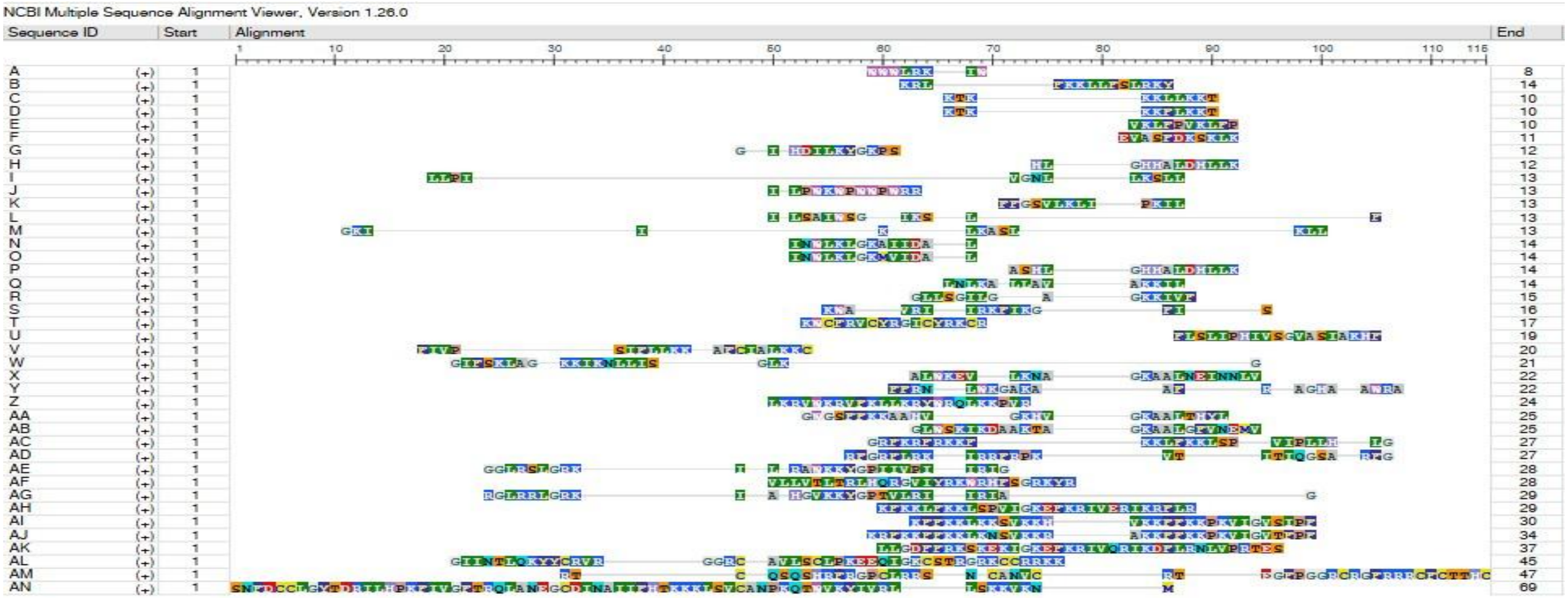

Supplementary Figure S2: Multiple sequence alignment of the 21 AMPs with wound healing activity using Clustal omega

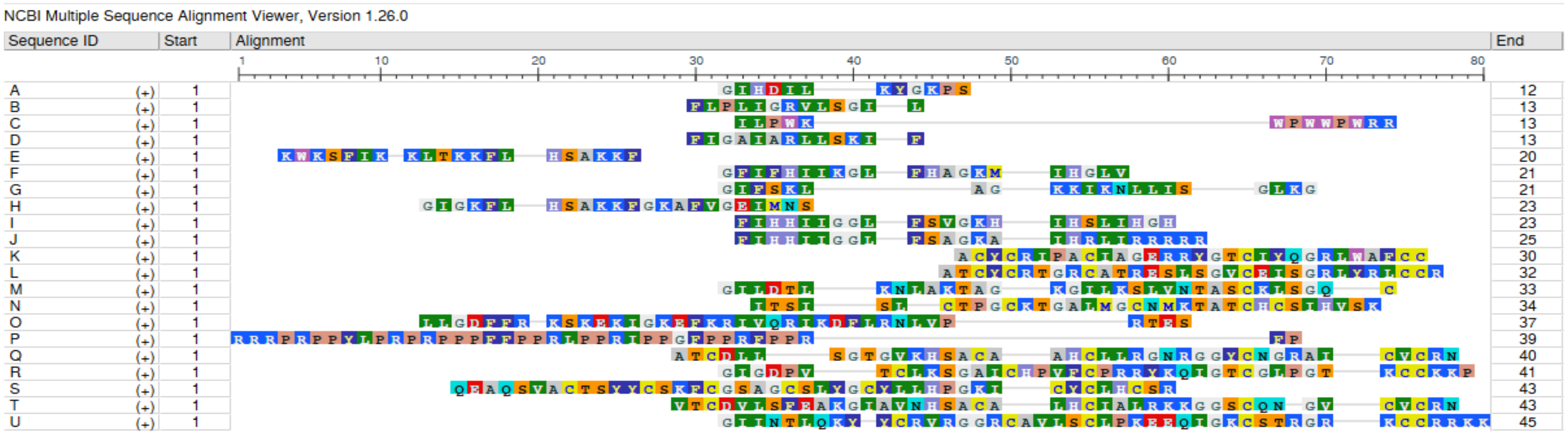

Supplement: Supplementary file 8 [file Image1.pdf]
